# Impact of the alkaline volatile trimethylamine on the physiology of *Streptomyces venezuelae*: an integrated transcriptomic and metabolomics study

**DOI:** 10.1128/spectrum.00318-25

**Published:** 2025-05-27

**Authors:** Yanping Zhu, Hanlei Zhang, Meng Liu, Xiuhua Pang

**Affiliations:** 1 The State Key Laboratory of Microbial Technology, Shandong University 12589, Qingdao, Shandong, China; 2 Suzhou Research Institute, Shandong University 12589, Suzhou, Jiangsu, China; 3 School of Municipal and Environmental Engineering, Shandong Jianzhu University 105835, Jinan, Shandong, China; University of Maryland Baltimore County, Baltimore, Maryland, USA; University of Sharjah, Sharjah, UAE

**Keywords:** *Streptomyces*, alkaline volatiles, TMA, organic acid, carbon metabolism

## Abstract

*Streptomyces* bacteria use volatile compounds for a range of functions, including communication and to kill competing species. We previously showed that the wild-type *Streptomyces venezuelae* strain ISP5230 produces the alkaline volatile trimethylamine (TMA), which can completely rescue the morphological defects of the *S. venezuelae* mutant strain MU-1, suggesting that TMA can have a significant impact on *Streptomyces* physiology. In this study, we further characterized strain MU-1 and elucidated the effects of alkaline volatiles on *S. venezuelae* by comparing the transcriptomic data of the wild-type strain ISP5230 and strain MU-1. Our RNA-sequence analysis revealed that MU-1 exhibited differentially expressed genes (DEGs) in multiple categories, including genes involved in development, carbon metabolism, antibiotic production, and transport, and these data were verified by real-time PCR analysis. Using metabolomics analysis, we next showed that organic acids accumulated in MU-1, consistent with the acidification of the growth medium by MU-1. In addition, our quantitative analysis showed much higher residual glucose content in the growth medium of MU-1, suggesting inefficient consumption of glucose by this mutant. Notably, TMA exposure restored the normal expression pattern of the DEGs in MU-1, consistent with the ability of TMA to rescue the MU-1 phenotype, including morphology, acidification of the growth medium, glucose uptake, and antibiotic production; these findings indicated a global impact of TMA on *S. venezuelae* physiology. In conclusion, our study suggests that the alkaline volatile TMA profoundly affects the physiology of *S. venezuelae* and may promote the growth of *S. venezuelae* mutants, enabling their survival in complex microbial communities.

**IMPORTANCE:**

Microbes produce a wide array of volatile compounds for which diverse biological roles have been implicated in microbial volatiles. In *Streptomyces*, the alkaline volatile trimethylamine (TMA) enables communication between cells, and in *Streptomyces venezuelae*, TMA could also rescue the defective morphology of the mutant strain MU-1, indicating a broader impact of TMA on *Streptomyces* physiology. In this study, we identified differentially expressed genes (DEGs) in MU-1 and demonstrated that TMA exposure induced normal expression of these DEGs in the mutant strain to levels comparable with those in the wild-type strain, consistent with the ability of TMA to rescue the abnormal phenotype of this mutant. Our study expands the role of TMA to a global impact on the physiology of recipient cells and adds a new understanding of the importance of volatile compounds in microbial communities.

## INTRODUCTION

*Streptomyces* is soil-dwelling gram-positive filamentous bacteria, best known as producers of bioactive compounds, including anti-infection agents and antibiotics, and for their ability to undergo cellular differentiation at late stages of development (1, 2). *Streptomyces* species have a relatively complex life cycle compared with most prokaryotic bacteria (2, 3). The life cycle of a *Streptomyces* cell starts with the germination of a spore; the germinated spore grows into the substrate to acquire nutrients, forming so-called substrate hyphae or vegetative hyphae. These vegetative hyphae then break the surface of the solid substrate and grow upward, forming aerial hyphae, which further develop into spore-bearing chains. Eventually, mature spores are released, initiating another cycle under suitable conditions (2). Therefore, *Streptomyces* species normally have three types of cells, including spores, vegetative hyphae, and aerial hyphae, at different growth stages. However, *Streptomyces venezuelae* is able to develop a newly discovered type of cell, designated as an “explorer cell,” at the “exploration stage,” which is initiated upon interaction with yeast (4). *Streptomyces* cells in the exploration stage can produce the volatile compound trimethylamine (TMA), and the *Streptomyces* explorer cells can communicate their exploratory behavior to other physically separated *Streptomyces* cells at other stages of growth using TMA (4). In addition to TMA, *Streptomyces* can produce a wide range of volatile compounds (5), including geosmin, which makes the well-known earthy odor (6). Although its biological role for the producer bacterium is not fully clear (7), there is strong evidence that geosmin can function in promoting *Streptomyces* spore dispersal through the recruitment of soil-dwelling arthropods (8). *Streptomyces* can also produce high amounts of volatile ammonium to kill a wide range of competing bacteria, including gram-positive and gram-negative species, over a long distance (9). However, the production of volatiles by microorganisms tends to be very conditional, and the process can be influenced by a multitude of factors, such as temperature, pH, and medium components (10). Production of TMA by *S. venezuelae* requires a specific growth condition, i.e., depletion of glucose in the medium (4, 11).

We previously found that the alkaline volatile TMA produced by *S. venezuelae* could rescue the defective morphology of *S. venezuelae* strain MU-1, which is a mutant strain with a bald phenotype (12), suggesting a potentially broad impact of TMA on the physiology of *S. venezuelae*. In this study, we revealed that MU-1 acidifies its growth medium and has reduced glucose consumption. We also evaluated the impact of TMA by transcriptomic and metabolomics analysis, and we found that TMA exposure could restore a normal expression pattern to genes that were differentially expressed in MU-1, likely explaining the rescue of the MU-1 phenotype by TMA exposure. Our study provides more insights into the biological role and mechanisms of action of TMA and into understanding the role of volatile compounds in the *Streptomyces* life cycle.

## RESULTS

### *S. venezuelae* mutant strain MU-1 acidifies the growth medium

**Fig 1:**
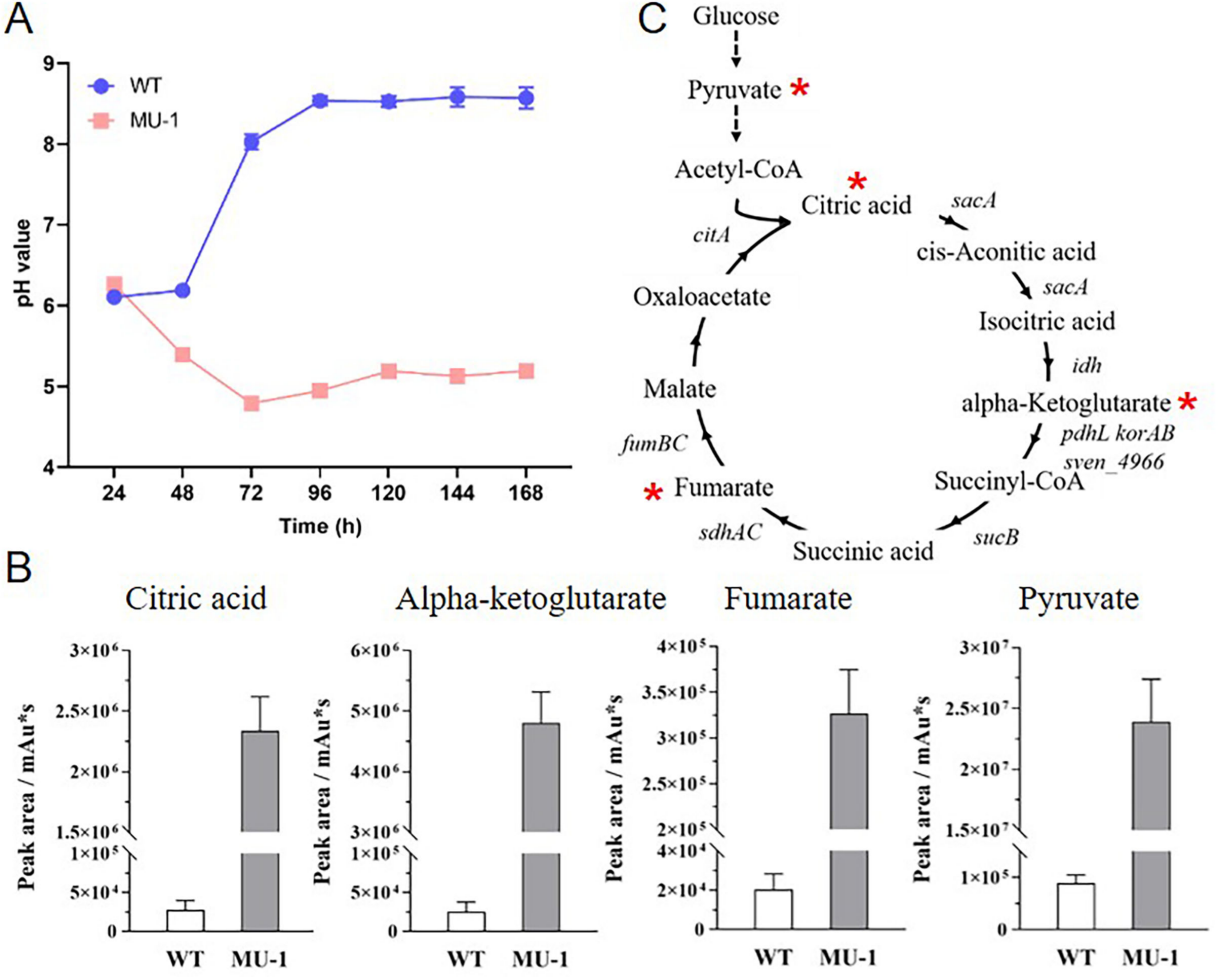
Accumulation of organic acids in the *S. venezuelae* mutant strain MU-1. (**A**) Temporal pH curves of the *S. venezuelae* wild-type strain ISP5230 (WT) and strain MU-1 grown at 30°C on solid N-Evans-CA medium. The pH value is measured for the growth medium of each strain. (**B**) Accumulation of organic acids in the growth medium of MU-1. *S. venezuelae* WT and MU-1 strains were grown at 30°C on solid N-Evans-CA medium for 96 h, and organic acids were measured by ultra-high-performance liquid chromatography with tandem mass spectrometry (UHPLC-MS/MS). Biological triplet samples were analyzed for each. The y-axis represents the value for the indicated metabolites in the corresponding strain. (**C**) Schematic representation of the tricarboxylic acid (TCA) cycle, showing the intermediates and the *S. venezuelae* genes encoding the enzymes that catalyze the steps. The organic acids that accumulate in MU-1 are marked by a red asterisk.

We previously showed that strain MU-1 is defective in the formation of aerial mycelium and spores, resulting in the bald phenotype shown in Fig. S1, and we also showed that this defective phenotype can be rescued by alkaline volatiles produced by the wild-type *S. venezuelae* strain ISP5230 (WT) (12). We also previously found that, due to the production of alkaline volatiles, including TMA, the growth medium of the WT was basic (12); additional analysis supported that observation, with the WT growth medium reaching a pH value of 8.5 at 96 h and maintaining a constant pH value afterward (Fig. 1A). In contrast, under the same growth conditions, the growth medium of strain MU-1 became acidic, reaching pH 4.7 at 72 h, and the medium maintained a level close to pH 5 even after prolonged incubation (Fig. 1A). The overall difference in pH value was over 3.5 pH points at 72 h and at time points afterward between WT and MU-1, suggesting the accumulation of acidic products, potentially organic acids, during the growth of MU-1.

### Metabolomics analysis reveals an accumulation of organic acids in MU-1

To test whether organic acids accumulated in MU-1, we performed metabolomics analysis and screened for metabolites with levels that were significantly changed between WT and MU-1 samples. Our metabolomics analysis showed that the levels of several metabolites from the tricarboxylic acid (TCA) cycle and glycolysis pathway were dramatically increased in MU-1, compared with WT (Fig. 1B and C). With TCA metabolites, the levels of citric acid, alpha-ketoglutaric acid, and fumaric acid in MU-1 were, respectively, about 6.4-fold (log_2_), 7.5-fold (log_2_), and 4.0-fold (log_2_) of that in WT. However, the metabolite with the most dramatic change was pyruvic acid of the glycolysis pathway, with a level in MU-1 of about 8.1-fold (log_2_) of that in WT (Fig. 1B). Collectively, our data revealed a remarkably high level of multiple organic acids in MU-1 samples, potentially explaining the acidification of the growth medium by MU-1.

### RNA-Seq analysis reveals a global change in gene expression in MU-1

We wanted to investigate the molecular mechanisms underlying the dramatic differences in morphology and in the accumulation of high levels of organic acids in MU-1, and we also wanted to investigate other potential gene expression differences that might not be reflected by the phenotypic changes. Therefore, we performed RNA-sequence (RNA-Seq) analysis on MU-1 using the WT strain ISP5230 as a control. Total RNA samples from ISP5230 and MU-1 were extracted from mycelia grown on N-Evans-CA medium, at two time points, 48 h and 96 h (Fig. S1). Our transcriptomic data indicated that, although thousands of genes were differentially expressed in MU-1, the fold change (log_2_) for most genes was lower than 2. Those differentially expressed genes (DEGs) with fold changes lower than 2 were not further analyzed. We also excluded DEGs with high fold changes but for which only minimal (<1) to low (<10) numbers of fragments per kilobase per million (FPKM) were detected in both strains. After these exclusions, at 48 h, 23 genes were upregulated, and 189 genes were downregulated; and at 96 h, 615 genes were upregulated, and 1,498 genes were downregulated in MU-1 compared with the parental strain ISP5230. Collectively, our RNA-Seq data indicated that more genes were downregulated than upregulated in MU-1. In general, the DEGs fell into multiple categories, including genes involved in development, carbon metabolism, antibiotic biosynthesis, and many more genes with unknown functions, indicating a global change in gene expression in MU-1.

### Differential expression of developmental genes in MU-1

To decipher the mechanisms underlying the bald phenotype of strain MU-1, we first analyzed the RNA-Seq data associated with developmental genes, including *ram* genes required for the synthesis of a surfactant protein that promotes the growth of aerial hyphae (13); the *chp* and *rdl* genes, which encode chaplet and rodlin proteins that coat the outer sheath of spores and aerial mycelium (14); the developmental regulatory *bld* genes that control the switch from vegetative hyphae to aerial hyphae; and *whi* genes that control the formation of mature spores (3). No notable difference was detected in the expression of any of these developmentally associated genes (*chp*, *rdl*, *ram*, *bld*, and *whi*) at 48 h in MU-1 (Table S1); however, all *chp* and *rdl* genes were profoundly downregulated in MU-1 at 96 h (Table S1), with changes ranging from −3.47 to −7.59 (log_2_), suggesting that much less chaplin and rodlet were produced in MU-1. In contrast, the *ram* genes, except *ramS*, appeared to be upregulated in MU-1, especially the levels for *ramR*, which were markedly higher at 96 h, reaching 3.73 (log_2_).

We next analyzed the RNA-Seq data for the *bld* and *whi* developmental regulatory genes at 96 h (Table S1). Only *bldM* (−2.77, log_2_) and *bldN* (−4.71, log_2_) were markedly downregulated in MU-1. Most *whi* genes, including *whiD*, *whiH*, *whiD*, *whiI*, and all *whiE* genes, were dramatically downregulated in MU-1 at 96 h, while *whiB* and *whiG* were only slightly downregulated in MU-1 at this time (Table S1). The generally lower expression of *chp*, *rdl*, *bld*, and *whi* genes in MU-1 is consistent with the dramatic morphological changes of this strain.

**Fig 2:**
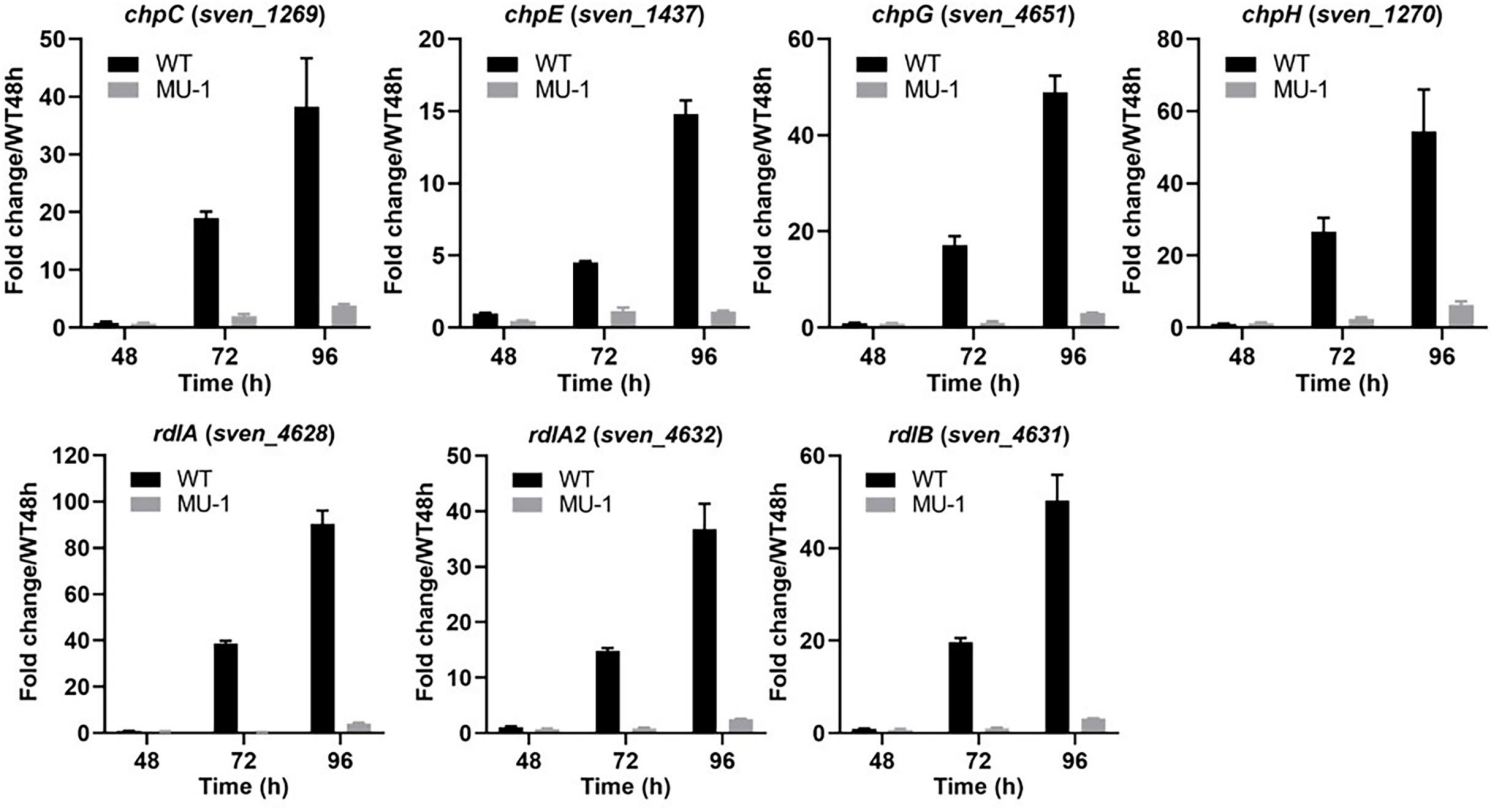
Transcriptional analysis of *chp* and *rdl* genes in strain MU-1. ISP5230 (WT) and MU-1 were grown on solid N-Evans-CA medium, and RNA samples were isolated at the indicated times. Gene expression was measured by real-time PCR, and expression of *hrdB,* encoding the major sigma factor in *Streptomyces*, was used as an internal control. The y-axis shows the fold change in the expression level of ISP5230 (black bars) and MU-1 (gray bars) over the level of each gene in ISP5230 at 48 h, at each time point, with the expression level of each gene in one set of ISP5230 samples at 48 h arbitrarily set to one. Results are the means (±SD) of triplet biological experiments.

**Fig 3:**
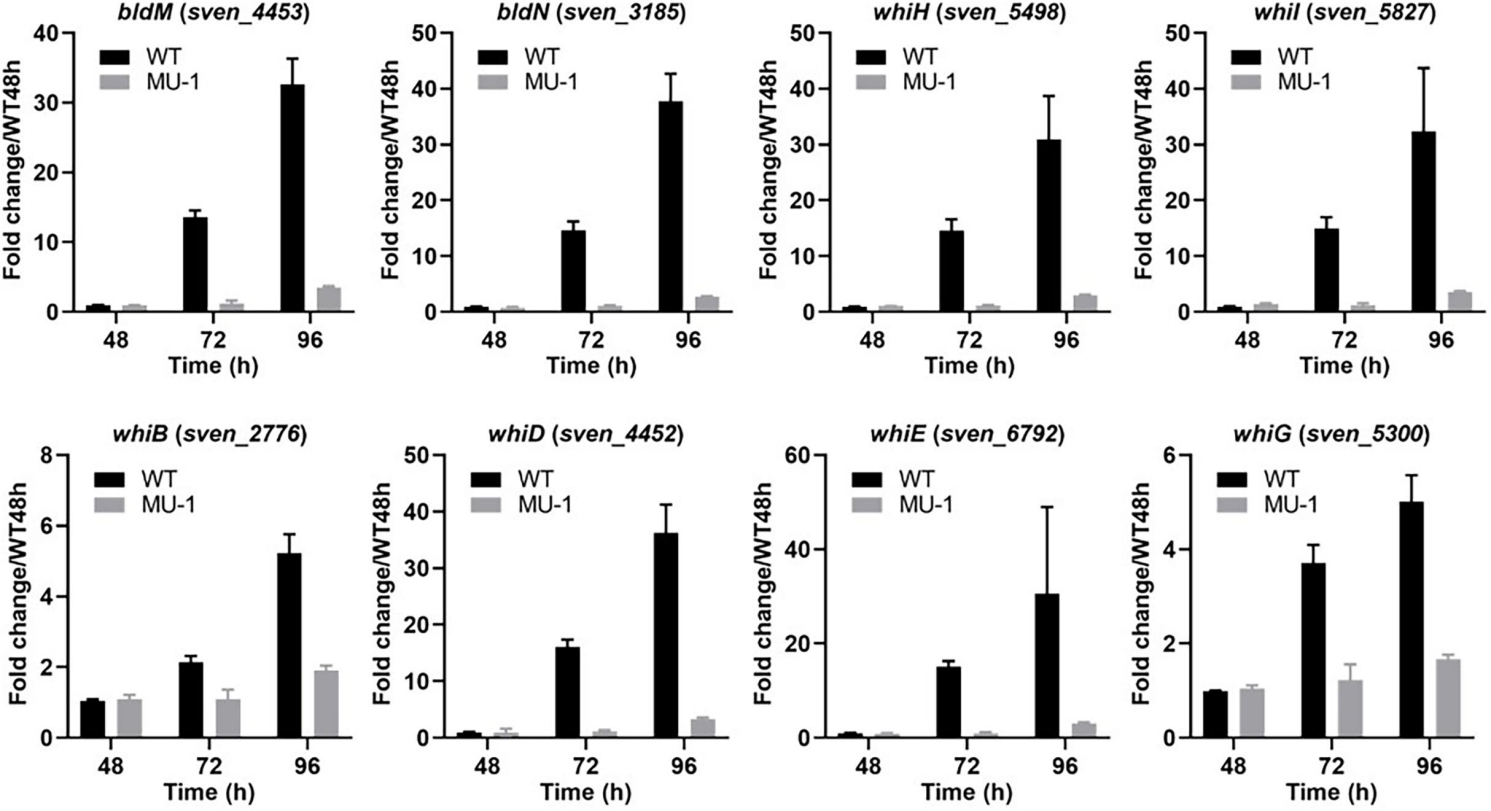
Transcriptional analysis of developmental regulatory genes in strain MU-1. ISP5230 (WT) and MU-1 RNA samples were prepared and analyzed by real-time PCR as described in the legend for Fig. 2. The y-axis shows the fold change in the expression level in ISP5230 (black bars) and MU-1 (gray bars) over the level of each gene in ISP5230 at 48 h, at each time point, with the expression level of each gene in one set of ISP5230 samples at 48 h arbitrarily set to one. Results are the means (±SD) of triplet biological experiments.

Expression of development-associated genes was also verified by real-time PCR using RNA extracted at three time points (48, 72, and 96 h) from both the WT strain ISP5230 and strain MU-1. Our transcriptional data showed that the four tested *chp* genes (*chpC*, *chpE*, *chpG*, and *chpH*) were developmentally regulated in ISP5230, demonstrating a temporal increase in expression and reaching the highest expression level at 96 h, ranging from a 14.8 ± 0.54 fold change for *chpE* to a 54.3 ± 6.7 fold change for *chpH*; however, this increase in expression was mostly abrogated in MU-1 (Fig. 2). A similar temporal expression pattern was observed for the three *rdl* genes, with a moderate expression level at 72 h and a peak level at 96 h, in ISP5230, whereas expression of these *rdl* genes was only minimal in MU-1 (Fig. 2), in agreement with the RNA-Seq analysis. The expression profiles of the developmental regulatory genes *bldM*, *bldN*, *whiB*, *whiD*, *whiE*, *whiG*, *whiH*, and *whiI* were similar to those of the *chp* and *rdl* genes in both ISP5230 and MU-1, although with some variation in the expression levels (Fig. 3), in agreement with the RNA-Seq analysis. Collectively, the dramatically reduced expression of development-associated genes in MU-1 was supported by real-time PCR results and RNA-Seq analysis and is consistent with the bald phenotype of MU-1.

### Differential expression of carbon metabolism genes in MU-1

To investigate the mechanisms underlying the accumulation of organic acids by strain MU-1, we analyzed the RNA-Seq data associated with carbon metabolism genes, including genes that are involved in the TCA cycle and glycolysis pathway, which produce multiple organic acid intermediates. Our RNA-Seq data indicated that the expression of most genes involved in the TCA cycle was not changed in MU-1 at 48 h, with only a few genes downregulated slightly at this time (Table S2); however, at 96 h, several TCA genes, including two *citA* genes, *fumC*, *sucB*, and the *sven_6835–6837* operon encoding succinate dehydrogenase (Fig. 1C), were expressed at markedly higher levels in MU-1 than in WT (Table S2), potentially explaining the high level of citric acid and fumarate in MU-1. For genes involved in the glycolysis pathway, only *sven_7329*, which encodes a fructose-bisphosphate aldolase, had an expression level above the cutoff value at 48 h, while expression of the rest of the pathway genes was either not detected or below the cutoff value at this time (Table S3). At 96 h, genes in the operon *sven_3052–54*, responsible for the uptake of fructose, and *sven_7344*, encoding a putative glyceraldehyde-3-phosphate dehydrogenase, were profoundly upregulated in MU-1, while *sven_0459*, encoding another putative glyceraldehyde-3-phosphate dehydrogenase, was markedly downregulated in MU-1, and other genes were upregulated only slightly in MU-1 (Table S3).

**Fig 4:**
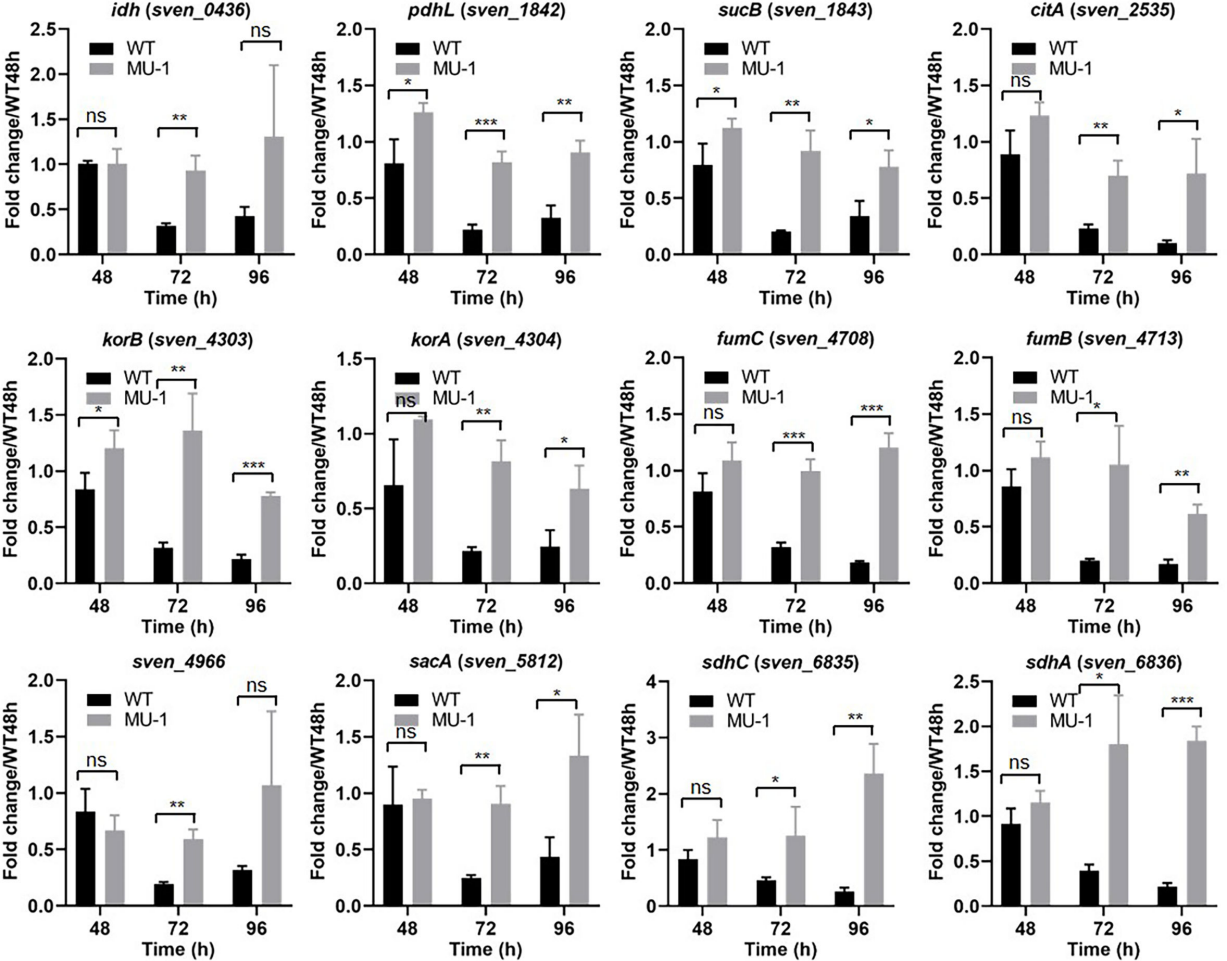
Transcriptional analysis of genes in the TCA cycle in strain MU-1. ISP5230 (WT) and MU-1 RNA samples were prepared and analyzed by real-time PCR as described in the legend for Fig. 2. The y-axis shows the fold change in the expression level in ISP5230 (black bars) and MU-1 (gray bars) over the level of each gene in ISP5230 at 48 h, at each time point, with the expression level of each gene in one set of ISP5230 samples at 48 h arbitrarily set to one. Results are the means (±SD) of triplet biological experiments. Statistical analysis was performed using Student’s *t*-test; *, *P* < 0.05, **, *P* < 0.01, and ***, *P* < 0.001.

Expression of carbon metabolism genes was also investigated by real-time PCR. Our transcriptional analysis demonstrated that the expression levels of all tested genes in the TCA cycle were comparable between MU-1 and the WT strain at 48 h (Fig. 4), which is consistent with the RNA-Seq analysis; however, the expression of these genes was generally higher in MU-1 than in WT at 72 h and 96 h (Fig. 4), consistent with the accumulation of TCA cycle-associated organic acids in MU-1. Because the overall expression levels and fold changes for the TCA genes were generally low, statistical analyses were conducted, which verified that most of the differences between WT and MU-1 at 72 h and 96 h were statistically significant (Fig. 4).

According to their expression profiles, genes of the glycolysis pathway were divided into three groups (Fig. S2): group 1 includes *gap2* and *eno*, which had expression levels much higher in WT than in MU-1 at 96 h; group 2 genes (*sven_1571*, *sven_1575*, and *sven_5075*) had expression levels much higher in MU-1 than in WT at 96 h; and group 3 genes (*sven_7329* and *sven_7344*) had markedly higher expression in MU-1 only at 48 h, while the differences at the two later time points were negligible (Fig. S2).

### Differential expression of pyruvate metabolism genes in MU-1

**Fig 5:**
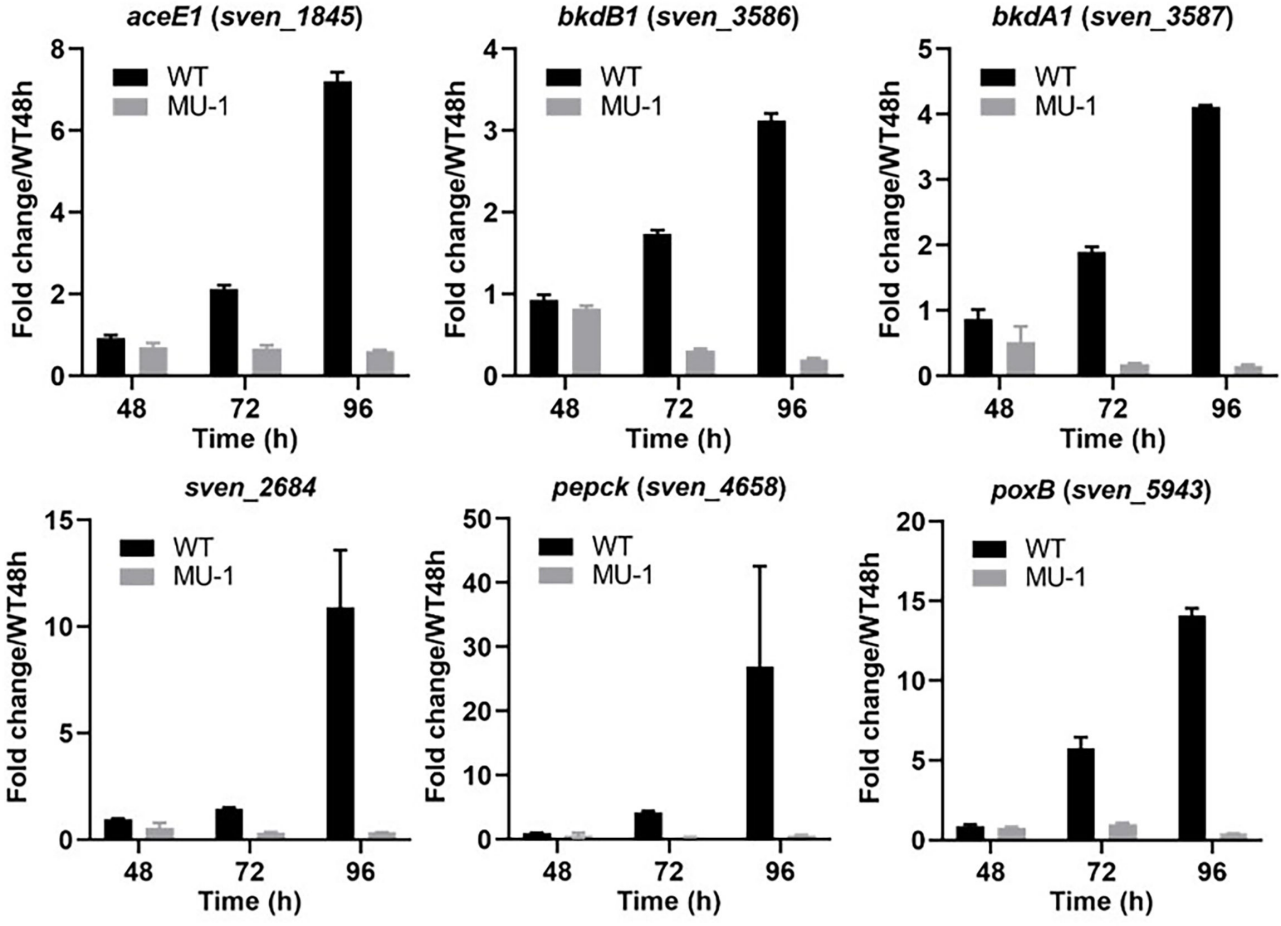
Transcriptional analysis of genes involved in pyruvate metabolism in strain MU-1. ISP5230 (WT) and MU-1 RNA samples were prepared and analyzed by real-time PCR as described in the legend for Fig. 2. The y-axis shows the fold change in the expression level in ISP5230 (black bars) and MU-1 (gray bars) over the level of each gene in ISP5230 at 48 h, at each time point, with the expression level of each gene in one set of ISP5230 samples at 48 h arbitrarily set to one. Results are the means (±SD) of triplet biological experiments.

We noticed that pyruvate was the most highly accumulated organic acid in MU-1 (Fig. 1B). Additionally, the expression level of *sven_5075*, encoding an enzyme (pyruvate kinase) involved in the biosynthesis of pyruvate, was elevated in MU-1 at 96 h in our RNA-Seq and real-time PCR analyses, potentially explaining the high level of pyruvate in MU-1. To further investigate the mechanism underlying pyruvate accumulation, we analyzed genes encoding enzymes that further process pyruvate. Our RNA-Seq data indicated that MU-1 had markedly downregulated expression of *aceE1*, *bkdA1*/*B1*, and *poxB*, each encoding a pyruvate dehydrogenase responsible for converting pyruvate into acetyl-CoA, and *sven_2684* and *sven_4658*, encoding enzymes for the turnover of pyruvate into other molecules (Table S4). Real-time PCR analysis showed that these genes were expressed only minimally in MU-1, contrasting with high expression levels in WT (Fig. 5), suggesting much less turnover of pyruvate in MU-1. Collectively, our data indicated higher expression of pyruvate biosynthetic genes and markedly lower expression of pyruvate metabolic genes in MU-1 than in WT, explaining the dramatic level of pyruvate accumulation in MU-1.

### Differential expression of antibiotic biosynthetic genes in MU-1

*S. venezuelae* produces two signature antibiotics, chloramphenicol (CHL) and jadomycin (JAD). To investigate the potential changes in antibiotic production in MU-1, we first analyzed the RNA-Seq data associated with the biosynthesis of these two antibiotics. Expression of most *cml* genes was markedly upregulated in MU-1 at 96 h but not at 48 h (Table S5). Additionally, transcription of four representative genes from the *cml* gene cluster was compared in the WT strain and MU-1 by real-time PCR analysis (Fig. S3A), and the results showed that expression of the four *cml* genes *cmlR*, *cmlI*, *cmlM*, and *cmlN* was generally higher in MU-1 than in WT at each time point (Fig. S3B), except for *cmlI* at 48 h. We therefore investigated whether the production of CHL is changed in MU-1. Our quantification analysis showed that the level of CHL for MU-1 was about twice the level found with WT at 72 h and 96 h (Fig. S3C), in agreement with the higher expression of *cml* genes in MU-1. The RNA-Seq data also indicated that expression of about half of the *jad* genes, involved in JAD biosynthesis, was upregulated in MU-1 at 96 h (Table S6); upregulation of these genes at late time points, represented by *jadw1*, *jadR2*, jadR*, and *jadY* (Fig. S4A), was verified by real-time PCR analysis (Fig. S4B). Collectively, our data indicated that several antibiotic biosynthetic genes were more highly expressed in MU-1.

Additionally, examination of RNA-Seq data further revealed that MU-1 had reduced expression of gene clusters for the biosynthesis of venezuelin (Table S7) and geosmin (Table S8) and a large gene (SVEN_7440) encoding an NRPS (15), suggesting potentially lower production of these natural products in MU-1.

### Reduced glucose consumption by MU-1

**Fig 6:**
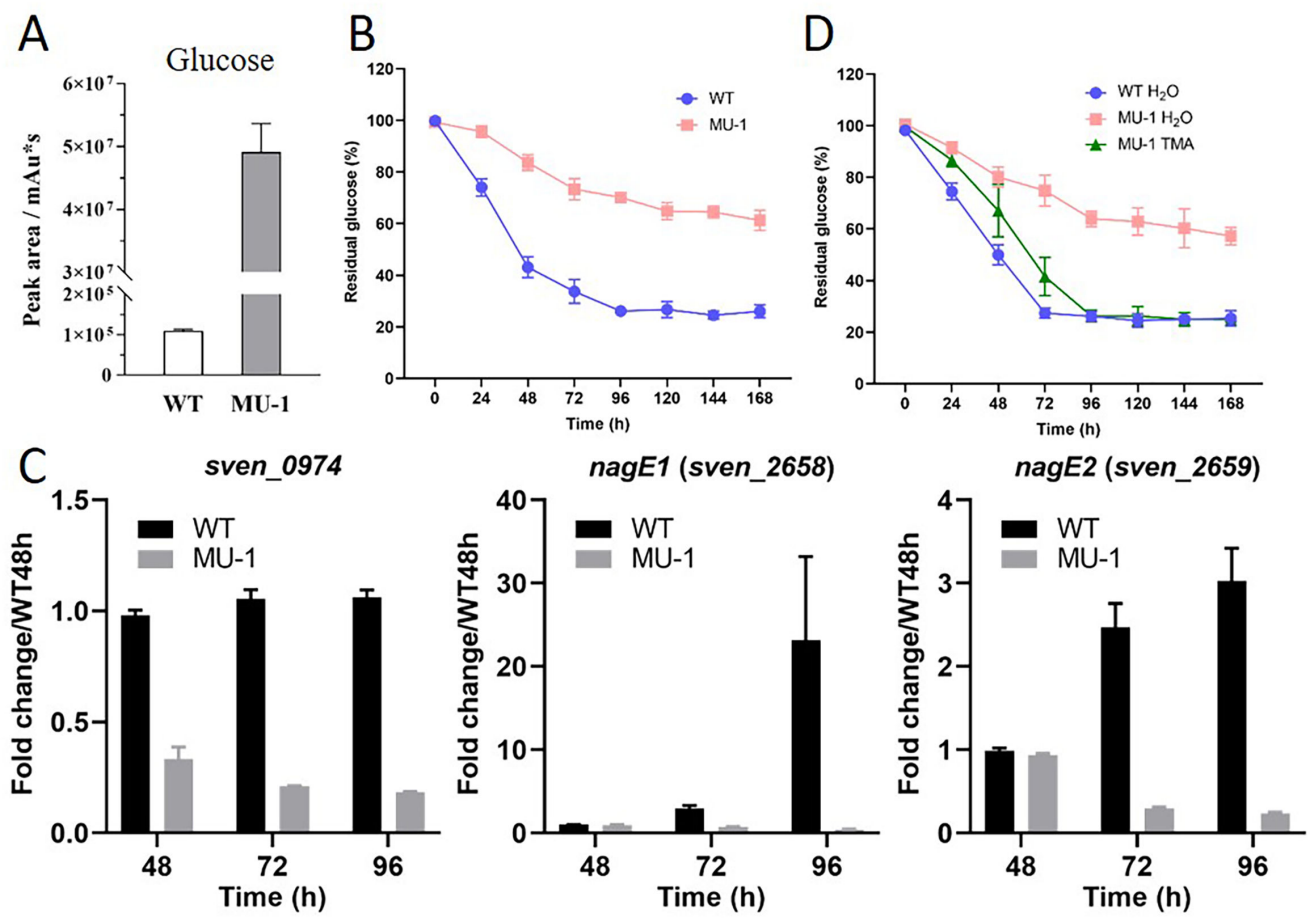
Evaluation of glucose content in the growth medium of MU-1 and expression of transporter genes. (**A**) Metabolomics analysis of glucose content in the growth medium of MU-1. *S. venezuelae* wild-type strain ISP5230 (WT) and MU-1 were grown at 30°C on solid N-Evans-CA medium for 96 h, and three samples were analyzed for each. The y-axis represents the value of glucose in the corresponding strain. (**B**) Temporal glucose content curves. ISP5230 (WT) and MU-1 were grown at 30°C on solid N-Evans-CA medium, and the residual glucose content of the medium was measured at the given time points. Results are the means (±SD) of triplet biological experiments. (**C**) Transcriptional analysis of transporter genes in strain MU-1. ISP5230 (WT) and MU-1 RNA samples were prepared and analyzed by real-time PCR as described in the legend for Fig. 2. The y-axis shows the fold change in the expression level in ISP5230 (black bars) and MU-1 (gray bars) over the level of each gene in ISP5230 at 48 h, at each time point, with the expression level of each gene in one set of ISP5230 samples at 48 h arbitrarily set to one. Results are the means (±SD) of triplet biological experiments. (**D**) Effects of TMA exposure on glucose content. Cultures of ISP5230 exposed to water (WT H_2_O) and MU-1 exposed to water (MU-1 H_2_O) or TMA (MU-1 TMA) were grown at 30°C on solid N-Evans-CA medium, and glucose content was measured as described for panel B. Results are the means (±SD) of triplet biological experiments.

Our metabolomics analysis revealed not only the accumulation of organic acids but also a high level of glucose in the growth medium of MU-1 (Fig. 6A), suggesting less consumption of glucose by MU-1. To investigate the dynamic consumption of glucose, we measured the residual glucose content in the growth medium of WT and MU-1 over 7 days (Fig. 6B). The glucose content decreased steadily from the initiation of growth (100% level) to 26.1% ± 0.3% at 96 h in WT, indicating an initial rapid consumption of glucose by WT. Glucose consumption then tapered off in WT, with the glucose content remaining fairly steady after 96 h, at about 26.7% ± 1.8% at 120 h, 24.6% ± 0.8% at 144 h, and 26.1% ± 0.4% at 168 h, suggesting exhaustion of the glucose accessible to bacteria, given that diffusion of glucose is limited in the agar. In contrast, the decrease in glucose content occurred very slowly with MU-1 (Fig. 6B), and the glucose content for MU-1 was at least 40% higher than for WT at 48 h, and times afterward, indicating that at least 40% less glucose was consumed by MU-1; this higher remaining glucose content in the growth medium of MU-1 was in agreement with the metabolomics analysis.

To investigate the mechanisms underlying the reduced glucose consumption with MU-1, we analyzed the RNA-Seq data associated with sugar transporter genes (Table S9). Our RNA-Seq data indicated that, while some unrelated transporter genes were profoundly upregulated in MU-1, three genes potentially involved in sugar transport (*sven_0974*, *sven_265*8, and *sven_2659*) were downregulated in MU1-1, especially *sven_2658* (*nagE1*), reaching a change of −6.97(log_2_), suggesting that much less sugar is transported into cells. Expression of these genes was also investigated by real-time PCR (Fig. 6C). The expression level of *sven_0974* was always higher in WT-1 than in MU-1 at 48 h (0.98 ± 0.01 vs 0.33 ± 0.03), 72 h (1.05 ± 0.02 vs 0.21 ± 0.01), and 96 h (0.98 ± 0.01 vs 0.33 ± 0.03). The expression level of *nagE1* was comparable at 48 h; however, expression was higher in WT at 72 h (2.94 ± 0.21 vs 0.76 ± 0.02), and dramatically higher at 96 h (23.5 ± 5.8 vs 0.44 ± 0.02), when compared to MU-1. Similarly, the expression level of *nagE2* was much higher in WT than in MU-1 at 48 h and 96 h. According to the transcriptional profiles, *sven_0974* was constitutively expressed at a fairly constant level in WT, implying it might encode a transporter for a critical energy supplier, for example, glucose, whereas *nagE1* and *nagE2* were developmentally regulated, with greater expression at later time points, suggesting a role in the transport of molecules that are needed at the late growth phase. Collectively, our data indicated that genes potentially involved in glucose transport were expressed at lower levels in MU-1 than in WT, suggesting reduced transport or uptake of glucose and consistent with the high level of residual glucose content in the growth medium of MU-1. Additionally, the reduced uptake of glucose suggests that MU-1 consumes less carbon and hence has reduced carbon metabolism, which potentially explains why MU-1 does not grow as robustly as the WT strain.

### TMA exposure complements the expression of developmental genes in MU-1

**Fig 7:**
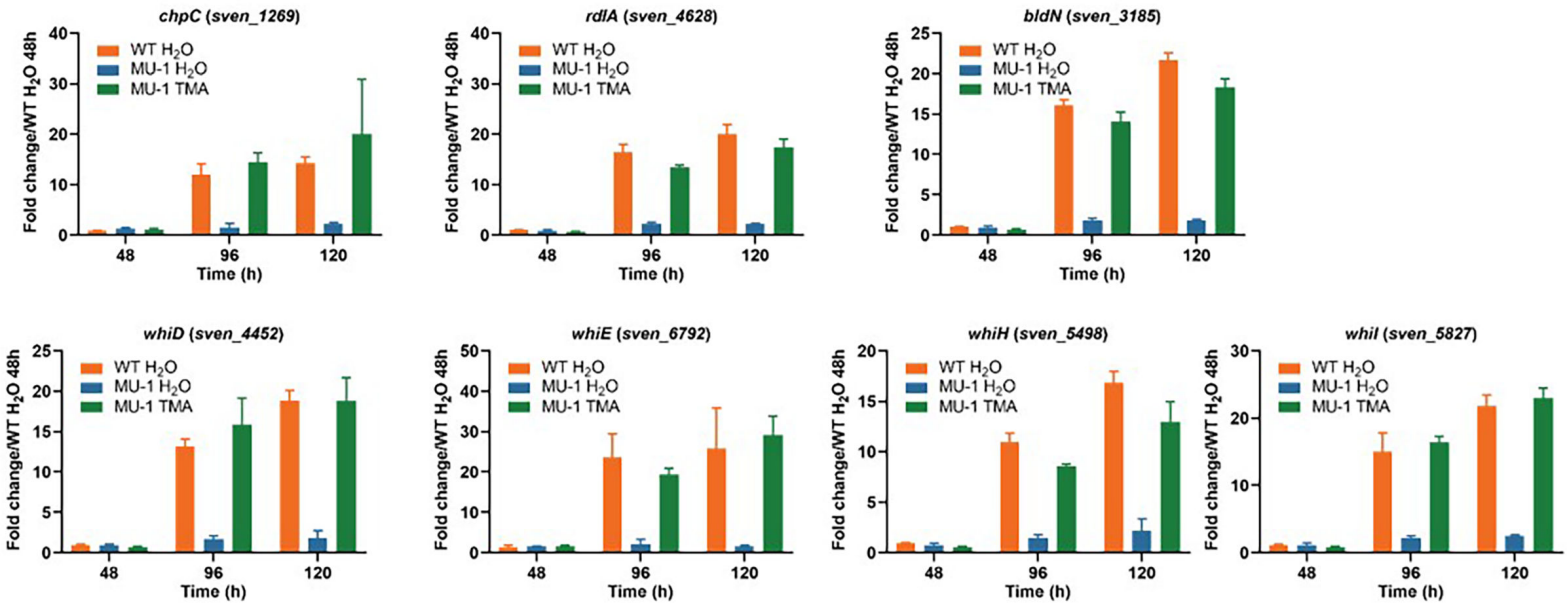
TMA exposure restores the expression of developmental genes in MU-1. Cultures of ISP5230 exposed to H_2_O (WT H_2_O) and MU-1 exposed to H_2_O (MU-1 H_2_O) or TMA (MU-1 TMA) were grown on solid N-Evans-CA medium, and RNA samples were prepared and analyzed by real-time PCR as described in the legend for Fig. 2. The y-axis shows the fold change in the expression level in WT H_2_O (orange bars), MU-1 H_2_O (blue bars), and MU-1 TMA (green bars) over the level of each gene in WT H_2_O at 48 h, at each time point, with the expression level of each gene in one set of WT H_2_O samples at 48 h arbitrarily set to one. Results are the means (±SD) of triplet biological experiments.

TMA exposure can rescue the morphological defect of MU-1, restoring the formation of aerial mycelium and spores, as demonstrated in this study (Fig. S5) and as reported previously (12). To investigate the underlying mechanism, the expression of development-associated genes was investigated by real-time PCR using RNA extracted at three time points (48 h, 96 h, and 120 h) from the mutant strain MU-1 exposed to water (MU-1 H_2_O) and TMA (MU-1 TMA), using the WT strain ISP5230 exposed to water (WT H_2_O) as a control. The expression level of *chpC* was close to a value of one for all three sample sets at 48 h when the strains were still in vegetative growth (Fig. 7). At 96 h, when the WT H_2_O control had already formed aerial mycelium (Fig. S5), the expression of *chpC* increased to a fold change of 12.1 ± 1.2; in contrast, expression remained at a basal level (1.5 ± 0.4) in MU-1 H_2_O, whereas *chpC* expression reached a level of 14.4 ± 1.1 in MU-1 TMA by 96 h. At 120 h, while *chpC* expression was still minimal in MU-1 H_2_O, the levels were comparable between WT H_2_O and MU-1 TMA. Similarly, the expression levels of *rdlA*, *bldN*, *whiD*, *whiE*, *whiH*, and *whiI* were restored in MU-1 TMA to levels comparable to those in WT H_2_O at the two late time points (Fig. 7) when MU-1 TMA forms aerial mycelium and spores (Fig. S5). Collectively, our data showed that TMA exposure induced the expression of developmental genes in MU-1 to levels comparable to those of the WT strain, consistent with the rescue of the morphological phenotype of MU-1 by TMA exposure.

### TMA exposure complements the expression of carbon metabolism genes in MU-1

**Fig 8:**
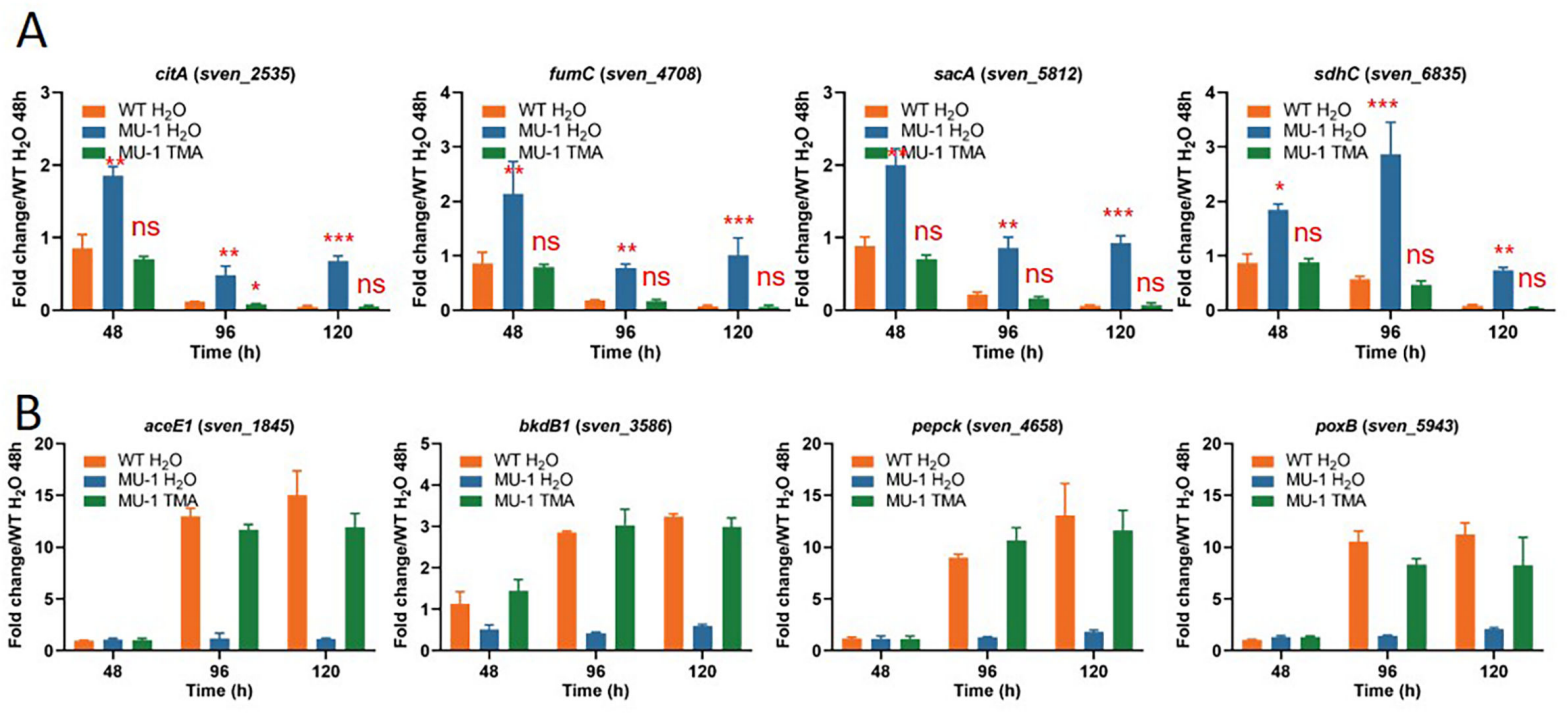
TMA exposure restores expression of carbon metabolism genes in MU-1. Transcriptional analysis of genes involved in (**A**) the TCA cycle and (**B**) pyruvate metabolism. Cultures of ISP5230 exposed to H_2_O (WT H_2_O) and MU-1 exposed to H_2_O (MU-1 H_2_O) or TMA (MU-1 TMA) were grown on solid N-Evans-CA medium, and RNA samples were prepared and analyzed by real-time PCR as described in the legend for Fig. 2. The y-axis shows the fold change in the expression level in WT H_2_O (orange bars), MU-1 H_2_O (blue bars), and MU-1 TMA (green bars) over the level of each gene in WT H_2_O at 48 h, at each time point, with the expression level of each gene in one set of WT H_2_O samples at 48 h arbitrarily set to one. Results are the means (±SD) of triplet biological experiments. (**A**) Statistical analysis was performed using Student’s *t*-test; *, *P* < 0.05, **, *P* < 0.01, and ***, *P* < 0.001.

TMA exposure not only rescued the morphological defect of MU-1 but also resulted in a rise in the pH of the growth medium of MU-1 from acidic to basic, with pH levels at 72 h and later comparable with those for the wild-type strain ISP5230 (Fig. S6); these findings suggested that the conversion of organic acids to other products was normal in MU-1 exposed to TMA. To investigate the mechanism underlying the rise in pH values, we examined the expression of genes involved in the TCA cycle and glycolysis pathway, since, as noted, these processes produce organic acid intermediates, and several of these genes were upregulated in MU-1 (Fig. 4; Fig. S2). For this analysis, we performed real-time PCR using RNA extracted at three time points (48 h, 96 h, and 120 h) with MU-1 exposed to TMA or water, as above. At 48 h, *citA* was upregulated in MU-1 H_2_O, with an expression level of about twice that in WT H_2_O (1.86 ± 0.07 vs 0.85 ± 0.11), whereas the expression level decreased to 0.71 ± 0.02 in MU-1 exposed to TMA, comparable to that in WT (Fig. 8). Due to the generally low levels of expression, the results for *citA* and other genes shown in Fig. 8A were confirmed by statistical analysis. At 96 h, by when vegetative growth had ceased and reproductive growth had already initiated, the expression level of *citA* had decreased to 0.12 ± 0.01 in WT H_2_O but remained much higher in MU-1 H_2_O (0.48 ± 0.07); in contrast, expression decreased to 0.08 ± 0.01 in MU-1 TMA, close to the value in WT H_2_O (Fig. 8A). Additionally, the generally higher expression levels of *fumC*, *sacA*, *sdhC*, *pdhL*, and *korB* in MU-1 H_2_O were restored in MU-1 TMA to levels comparable to those in WT H_2_O (Fig. 8A; Fig. S7A), indicating expression of genes involved in the TCA cycle was restored in MU-1 after TMA exposure. Similarly, the expression of genes involved in the glycolysis pathway (Fig. S7B) and in the metabolism of pyruvate (Fig. 8B; Fig. S7C) was restored in MU-1 TMA to levels comparable to those in WT H_2_O. Overall, these data indicated that TMA exposure of MU-1 restored the normal (i.e., WT) expression pattern of genes that impact the levels of organic acids, explaining the pH increase in MU-1 after TMA exposure.

### TMA exposure restores antibiotic production by MU-1

We also measured the levels of CHL produced by MU-1 exposed to TMA. Although the CHL level was higher in MU-1 H_2_O than in the WT H_2_O strain at 96 and 120 h, the levels were comparable between MU-1 TMA and WT H_2_O at these two times (Fig. S8A), indicating that TMA exposure restored normal production levels of CHL to MU-1. Our transcriptional analysis showed that the higher levels of *cmlI*, *cmlM*, and *cmlN* expression detected in MU-1 H_2_O at each time point were reduced in MU-1 TMA to levels comparable to those in WT H_2_O (Fig. S8B and C), consistent with the reduced production of CHL. In addition, normal expression of *jadR2*, jadR*, and *jadY* was restored in MU-1 TMA (Fig. S9). These data indicated that TMA exposure reduced the expression of *cml* and *jad* genes to a WT level.

### TMA exposure promotes glucose consumption by MU-1

We revealed high residual glucose content in the growth medium of MU-1 (Fig. 6A and B). Given the complementation of the morphological defects, acidic pH, and abnormally high CHL production in MU-1 by TMA exposure, we were curious if normal glucose consumption would also be restored. Notably, the temporal glucose content curve of MU-1 TMA nearly overlapped that of WT H_2_O, which had at least a 30% lower glucose content than MU-1 H_2_O did at 48 h and times afterward (Fig. 6D), indicating that TMA exposure promoted glucose consumption by MU-1. Our transcriptional analysis showed that expression of the sugar transport genes *sven_0974* and *nagE1* was restored to a level comparable with that of the wild-type strain (Fig. S10), consistent with an increased uptake and transport of glucose by MU-1 exposed to TMA and explaining the lower glucose content in the growth medium of MU-1 TMA.

We previously showed that the alkaline volatile ammonium also raises the pH of the growth medium of MU-1, as TMA did; however, the morphology of MU-1 was not rescued by ammonium treatment (12). To investigate whether ammonium exposure could restore normal glucose consumption to the mutant, we measured the glucose content in an ammonium-treated sample. However, the glucose content in the growth medium of MU-1 exposed to ammonium was comparable to that of the control MU-1 sample (Fig. S11), indicating that, unlike TMA, ammonium exposure does not promote glucose consumption.

### Sequence changes in MU-1

Genes responsible for TMA production have not yet been cloned or mapped in *Streptomyces* (4). Therefore, due to the reduced production of TMA by MU-1 (12), the genome of MU-1 was sequenced and compared with that of the WT strain ISP5230 to help identify the genes and sequence changes potentially associated with TMA production. However, multiple sequence changes were detected in MU-1 (Table S10), making it difficult to identify the specific changes responsible for the reduction in TMA production. As expected, SVEN_1780 and SVEN_1781 were missing from MU-1 (12). SVEN_1780–1781 encodes homologs of the *Streptomyces coelicolor* two-component system MacRS (16), and we deleted them from ISP5230 in our previous study, generating MU-1 (12); however, the inability of SVEN_1780–1781 to complement the phenotype of MU-1 indicated the presence of other mutations, which was supported by our sequencing results. These additional mutations included an insertion mutation in SVEN_1676, encoding a glutamate synthase. There were also two missense mutations in SVEN_1881, encoding an acyl transferase domain in a polyketide synthase, and one missense mutation in SVEN_6499 and six missense mutations in SVEN_4066, each encoding hypothetical proteins. Unexpectedly, 10-point mutations were detected in the sequence encoding 23S ribosomal RNA, and mutations were also detected in the intergenic regions for some genes. Although one or more of these mutations may be associated with TMA production, their involvement needs to be validated in future studies.

## DISCUSSION

Microbes produce a wide range of volatile compounds that are usually low-molecular-weight molecules and thus can spread easily in soil, air, and water (5, 17). Diverse biological roles, including antibacterial and antifungal activities, enhancing plant growth, raising the pH, altering antibiotic resistance profiles, protist attractant, and others, have been revealed for these microbial volatiles (5, 17–20); most often, the effect of volatiles appears to be uni-directional, manifested mainly by changes in organisms perceiving the volatiles rather than the producer itself (21). We previously demonstrated a strain-specific effect of the *Streptomyces* volatile TMA, which was able to rescue the morphological defects of an *S. venezuelae* mutant strain (MU-1), a newly discovered role for bacterial volatiles (12). In this study, by integrated transcriptomic and metabolomics analyses, we further revealed a global impact of TMA on the physiology of *S. venezuelae*, rather than the limited effect on one or two traits reported for other known volatiles (5). We showed that TMA exposure induces the expression of developmental genes to levels comparable with those of the wild-type strain, which would explain the ability of TMA to restore the normal formation of aerial mycelium and spores to MU-1. Our findings also revealed a profound effect of TMA on carbon uptake and metabolism, with consequences for the acidity of the growth medium and the robustness of strain growth. Changes in the expression of genes involved in the TCA cycle and pyruvate metabolism were associated with the accumulation of multiple organic acids in the growth medium of MU-1, an effect that could be counteracted by TMA exposure. These findings indicated that TMA impacts carbon metabolism, including glycolysis and the TCA cycle.

Similarly, TMA exposure restored normal glucose uptake to MU-1, indicating broad effects on carbon-related and energy-related processes. Since TMA also influences development, these findings support a link between normal carbon/energy metabolism and progression to the later stages of development.

Normally, an alkaline pH promotes growth, and an acidic pH represses the growth of microbes. *citA* encodes the major citrate synthase that catalyzes the synthesis of citrate from oxaloacetate and acyl-CoA, and *acoA* encodes an aconitase that catalyzes the reversible isomerization of citrate to isocitrate; mutation of either of these genes in *S. coelicolor* led to the accumulation of organic acids and a marked decrease in the pH value of the growth medium (22, 23). Additionally, the mutation of *cya*, which encodes an enzyme for the synthesis of cyclic AMP, also led to the accumulation of organic acids by *S. coelicolor* under specific growth conditions (24). Mutant strains of these genes were also developmentally blocked, forming a bald phenotype (22–24), similar to that of MU-1 grown on N-Evans-CA (12). However, the morphological defects could be rescued by the addition of a pH buffer, suggesting that an acidic pH was responsible for the repressed growth and development of these mutant strains (22–24). In contrast to those mutants, although the low pH value of the growth medium of MU-1 potentially retarded the growth of this strain, the low pH was not responsible for the bald phenotype of MU-1. MU-1 still displayed a bald phenotype after ammonium exposure, which generated an alkaline pH in the growth medium of MU-1, comparable to that of the growth medium of the WT strain (12). These findings indicate that other factors are responsible for the bald phenotype of MU-1, although the acidification of the growth media by MU-1 is likely caused by the accumulation of organic acids, as found with the *citA*, *acoA*, and *cya* mutants.

Our transcriptional analysis showed that *bldN*, which encodes sigma factor BldN, was markedly downregulated in MU-1; BldN targets include *chp*, *rdl*, and also *bldM* (25), explaining the profoundly decreased expression of these genes in MU-1, which was also consistent with the bald phenotype of this strain. Since MU-1 does not form aerial mycelium and spores, the expression levels of the developmentally regulated *whi* genes would also be expected to remain low, which was supported by our findings. Notably, we detected increased expression of *whiG* in the late developmental stages of our WT strain, a pattern that was abrogated in MU-1. The activity of the sporulation-specific sigma factor WhiG has been reported to occur primarily through post-translational regulation, including by an anti-sigma factor and 3’,5’-cyclic diguanylic acid, rather than at the expression level (26). However, strain variation and potential differences in growth conditions may potentially explain why we observed some developmental changes in *whiG* expression in our WT strain.

It was previously reported that TMA was produced by *S. venezuelae* when the growth conditions included depletion of glucose and interaction with yeast (4, 11), which presented a seeming paradox, given our finding that TMA can be produced by *S. venezuelae* grown on solid agar with glucose (12). However, our current study suggests that incomplete diffusion of glucose through a solid substrate can create a localized glucose-depleted environment that would favor TMA production, which our findings indicate occurred with the wild-type strain by 96 h under our growth conditions; by this time, MU-1 started forming aerial mycelium when co-incubated with the wild-type strain (12), consistent with the production of TMA by wild-type *S. venezuelae* and with the need for a glucose-depleted environment (4). In addition, the high level of glucose that was maintained in the growth medium of MU-1, even after prolonged incubation, may have inhibited TMA production, potentially explaining why strain MU-1 could not produce TMA.

Ammonium is another alkaline volatile produced by *S. venezuelae* (9). Although co-incubation with ammonium or TMA could raise the pH of the MU-1 growth medium, the defective morphology of MU-1 was not restored by ammonium exposure (12), suggesting that the rescue of the MU-1 morphology, and potentially other defective traits of MU-1, is TMA specific. Although our study indicated that the induction of a more normal gene expression pattern is responsible for the ability of TMA exposure to complement several of the abnormal traits of MU-1, the mechanism by which TMA, a low-molecular-weight molecule capable of penetrating the bacterial membrane, triggers a global positive effect is still not known. Although we did not examine gene expression patterns under ammonium exposure, the failure of ammonium to rescue the morphology and other traits of MU-1 suggests that ammonium exposure did not stimulate a normal pattern of gene expression, including for carbon metabolism genes, and therefore, ammonium exposure could not promote glucose consumption by MU-1, in contrast to TMA. Instead, the high level of glucose that remained in the medium during ammonium exposure likely prevented TMA production, which is required to compensate for the defects of MU-1. However, ammonium still appears to be beneficial to the producer by killing the surrounding bacteria (9), whereas the production of TMA could be beneficial to the producer by inducing an exploratory state to obtain nutrients (9), and beneficial to the receiver as shown by the global positive impact on MU-1. Additionally, given that MU-1 produced higher than normal levels of CHL, TMA production could also enhance the survival of the producer by rescuing mutants that are less fit but that might still contribute to products that benefit the TMA producer. The importance of microbial volatiles for the ecology of microorganisms has been understudied for years. However, as basic environments normally favor bacterial growth while acidic environments favor fungal growth, it is possible that *Streptomyces* species promote the richness of the bacterial community in the environment by emitting alkaline volatiles such as TMA.

## MATERIALS AND METHODS

### Culture conditions

*S. venezuelae* strain ISP5230 is used as the wild-type strain (27), and MU-1 is a derivative strain of ISP5320 defective in growth (12). N-Evans-CA agar comprises Basic Evans medium (25 mM Tris[hydroxymethyl]methyl-2-aminoethanesulphonic acid (TES), 10 mM KCl, 2 mM NaSO_4_, 2 mM citric acid, 0.25 mM CaCl_2_, 1.25 mM MgCl_2_, 1 mM NaMoO_4_, and 0.5% Evans trace elements, pH 7.2) supplemented with glucose (2.5%) as carbon source, casamino acids (1%) as nitrogen source, NaH_2_PO_4_ (2 mM) as phosphate source (28), and agar. N-Evans-CA was used for incubation of *S. venezuelae* strains, measurement of pH, determination of organic acids, RNA extraction, and metabolomics analysis.

### Co-incubation analysis and measurement of pH

Equal numbers of *Streptomyces* spores were inoculated onto N-Evans-CA media and were incubated at 30°C at different times. For all experiments, solid agar was weighed first and cut into small pieces. An equal weight of distilled water was then added, followed by vortexing for 2 h. The mixture was centrifuged to remove debris, and pH levels were measured as described (12).

### Metabolomics analysis

The frozen bacterial samples were placed in microcentrifuge tubes and resuspended with pre-chilled 80% methanol, thawed on ice, and whirled on an agitator for 30 s. After sonification for 6 min, the mixtures were centrifuged at 5,000 rpm, 4°C for 1 min. The supernatants were freeze-dried, resuspended with 10% methanol, and injected into a liquid chromatography with tandem mass spectrometry system.

Ultra-high-performance liquid chromatography with tandem mass spectrometry (UHPLC-MS/MS) analyses were performed using a Vanquish UHPLC system (Thermo Fisher, Germany) coupled with an Orbitrap Q Exactive TM HF mass spectrometer or Orbitrap Q Exactive TMHF-X mass spectrometer (Thermo Fisher, Germany). Samples were injected onto a Hypersil Gold column (100 × 2.1 mm, 1.9 µm) using a 12 min linear gradient at a flow rate of 0.2 mL/min. The eluents for the positive and negative polarity modes were eluent A (0.1% FA in Water) and eluent B (methanol). The solvent gradient was set as follows: 2% B, 1.5 min; 2%–85% B, 3 min; 85%–100% B, 10 min; 100%–2% B, 10.1 min; and 2% B, 12 min. The Q Exactive TM HF mass spectrometer was operated in positive/negative polarity mode with a spray voltage of 3.5 kV, capillary temperature of 320°C, sheath gas flow rate of 35 psi, and auxiliary (aux) gas flow rate of 10 L/min, S-lens RF level of 60, and aux gas heater temperature of 350°C.

The raw data files generated by UHPLC-MS/MS were processed using Compound Discoverer 3.3 (CD3.3, Thermo Fisher) to perform peak alignment, peak picking, and quantitation for each metabolite. The main parameters were set as follows: peak area was corrected with the first quality control (QC) evaluation, actual mass tolerance, 5 ppm; signal intensity tolerance, 30%; and minimum intensity, and other parameters recommended by the manufacturer. After that, peak intensities were normalized to the total spectral intensity. The normalized data were used to predict the molecular formula based on additive ions, molecular ion peaks, and fragment ions, and then peaks were matched with the mzCloud (https://www.mzcloud.org/), mzVault, and MassList databases to obtain the accurate qualitative and relative quantitative results. Statistical analyses were performed using the statistical software R (R version R-3.4.3), Python (Python 2.7.6 version), and CentOS (CentOS release 6.6). When data were not normally distributed, they were standardized according to the formula: sample raw quantitation value/(the sum of sample metabolite quantitation value/the sum of QC1 sample metabolite quantitation value) to obtain relative peak areas; compounds whose cutoff values of relative peak areas in QC samples were greater than 30% were removed, and finally, the metabolite identification and relative quantification results were obtained.

### RNA isolation, reverse transcription-PCR, and real-time PCR

Sequences of primers used in this study are listed in Table 1. For the RNA extractions, equal numbers (about 2 × 10^6^) of *Streptomyces* spores for wild-type and mutant strains were grown at 30°C on N-Evans-CA agar solid medium, covered with cellophane, and the mycelia were collected at multiple times. Collected mycelia were ground in liquid nitrogen and then dispensed into REZol reagent (SBSBIO). Chloroform was added to the mixture, and the mixture was then vortexed for precipitation of cellular proteins. The supernatant was transferred to RNase-free Eppendorf tubes after centrifugation for 10 min at high speed; absolute ethanol was then added, and the samples were mixed and then transferred to absorption columns. The columns were centrifuged to remove the liquid, washed twice with washing buffer, and eluted twice with Diethyl Pyrocarbonate (DEPC) water. The eluates were then collected, and crude RNA was precipitated with the addition of absolute ethanol and sodium acetate. To remove the chromosomal DNA, the crude RNA samples were treated twice with “Turbo DNA-free” DNase reagents (Ambion), and then reverse transcription was conducted as described (27). The SYBR Green *Pro Taq* HS DNA polymerase (AG Bio) was used with the manufacturer’s recommended conditions to determine the melting curve of PCR products and their specificity, and for real-time PCR assays, using a Roche LightCycler480 thermal cycler. Expression of the *hrdB* gene, which encodes the *Streptomyces* major sigma factor, was used to normalize the relative quantities of cDNA.

**TABLE 1:** Primers used in this study

Primers for real-time PCR	Sequence (5′−3′)
*hrdB* forward/reverse	CCAGATTCCGCCAACCCA/CTTCGTCACGGTCGTCCTG
*sven_0436* forward/reverse	ACGACAAGATCAAGGGCTCG/AGTCGTTCTCGCTCATGGTG
*sven_0459* forward/reverse	GGTCAGCATCCTCAAGACCC/TTGTCGGTCTTGTACTCGGC
*sven_0913* forward/reverse	TCGACATCGACATCCACACC/GGAACTCGGTCTCGCTCTCC
*sven_0915* forward/reverse	TGTTCATGTTCCTCGTCGGC/GAGCAGACGATCGATCCCAC
*sven_0924* forward/reverse	ACGAGAAATCCGAAGCCGC/GTAGGCGACCCAGCCCCAG
*sven_0925* forward/reverse	GCACGCTGCTGAACGGACT/AGGAGAAGATTCCGACCGA
*sven_0974* forward/reverse	GGTCGTCGTCTCCATGCAC/GTGTCGCCCTTCTTGACGA
*sven_1089* forward/reverse	CGAATACGCAAAGCAGCTCG/GCTCGTAGGAACCGACCAC
*sven_1269* forward/reverse	AGGCAGGGAACAAGCTATGC/AATTCATGGCCGCACCGTTC
*sven_1270* forward/reverse	ATCAAGAAGGTCGTCGCTGC/GACATTGCCCGAGAGGACAC
*sven_1437* forward/reverse	CAAGGCCGTCGAGTCGC/GTGTTGCCGGAGACGTTCAC
*sven_1571* forward/reverse	CAAGCCGATCAAGAACGTCG/ACGTTGGAGACGAAACGGAA
*sven_1575* forward/reverse	CCAAGGTCTCCGACAAGCTC/GGCCTTGAGGAAGGTGTACG
*sven_1842* forward/reverse	CGTCGCACTGATCGAGAAGA/TACTTGTGCACGGCCTTGAT
*sven_1843* forward/reverse	TCATCAACGCCCGGATCAAC/ACCGCGATACCGATGTTCTC
*sven_1845* forward/reverse	TCAACGGCCAGATCCTCAAC/CCAGCCGAACATCGAGTAGA
*sven_2535* forward/reverse	TTCGACATCGGGAAACTCCG/TCACCGTCGAGGTAGGTGAT
*sven_2658* forward/reverse	CCTCGTCTACTACACGGTGC/TTCTGGTACGTGTAACCCGC
*sven_2659* forward/reverse	TCATGGGTGTCTTCTTCGGC/GACGGTGTTGACGAACTGGT
*sven_2684* forward/reverse	GAGACCATCGATCACACCCC/CGAAGGCCGTGGAGTAGTAG
*sven_2776* forward/reverse	ACCGATCCCGAGTCCTTCTT/AGGGCGTATTCGAGGCATTC
*sven_2899* forward/reverse	AACATCCTCAACGGTGGGTC/GCCTTGAGGGTGTGGTAGAT
*sven_3052* forward/reverse	TCTGACGATCCTGTCCTCCC/GGACTCTTCCTCGGCTTCTT
*sven_3054* forward/reverse	CGACCTGACCGACATCGAC/CGCGGACGACTTCCGTTC
sven_3185 forward/reverse	CCTCACCAGTGAGACGTTCC/CTGGACTTGAAGTGGTCGGC
*sven_3586* forward/reverse	CCAGTTCGACGGTTTCGTCT/GATGACGACCGGCATCTTGA
*sven_3587* forward/reverse	GACGACGACTACGTCTTCCC/ATTCCCAGCAGATTCGTCGG
*sven_4303* forward/reverse	TGGCGAAGGAGAACATCGTC/GATCGAGTGCATCCCGTAGG
*sven_4304* forward/reverse	GCAGAAGTTCGCGAAGAAGC/CCGAGATGTTCCGGTAGGTG
*sven_4452* forward/reverse	AACTCGGCGAAAGAGGTCTG/ATGAGCTCTTCGCGTTCGTC
*sven_4453* forward/reverse	ATGACATCCGTTCTCGTCTGCG/GAGGACTTCCTCGCCGTTGG
*sven_4628* forward/reverse	TTCCATCGTCGGTCTCATCC/GAGTTCTCGGTGCACTGCTG
*sven_4631* forward/reverse	GGACATCAACGTCCTGTCCT/GATGTCGTCCAGGATGTGCG
*sven_4632* forward/reverse	CAGTCCTTCGGCAACTCGAA/AGAGCTTGTTCAGCGAGCC
*sven_4651* forward/reverse	GCAACACCGTCAACGTCATC/GTCACCCTTGTCGTGACCG
*sven_4658* forward/reverse	CTGCTCGGCAAGAAGTGCTA/GGGGTGAGCTTGAGGATCAG
*sven_4708* forward/reverse	ATGAACGACACAGACCAGGC/GAGACCGGGAAGTTCTCCAC
*sven_4713* forward/reverse	TTGCGTACTCCGATCTGCTC/AGGAAGTGCTGGATGTCGTG
*sven_4966* forward/reverse	ATGATGAAGCTCCGCGACAT/GATGTGCATGAACTCGACGC
*sven_5075* forward/reverse	CAAAATCGTCTGCACGCTGG/CGTGGCTGAAGTTGAATCGG
*sven_5300* forward/reverse	CTGATCCTGCACTACTCGCC/GATGGCGTACGTCTCGAACT
*sven_5498* forward/reverse	CTTGCGCACACCATGATGAC/GTAGGGGTAGCGGTCGAGAT
*sven_5812* forward/reverse	GAAGGAGACTGTCGTGTCGG/CTTCAGGCTGTAAGGGAGGC
*sven_5827* forward/reverse	CTACCGCCCGAACAAGCC/TGGAAGAAGCCTCGATCACG
*sven_5943* forward/reverse	GGATCCAGTACGACAACCCG/CAGGATCAGCAGATCGCACT
*sven_6792* forward/reverse	TCATCGAGTCGGAACAGGAC/TCCCAGCTGTAGAAGCACCG
*sven_6835* forward/reverse	CATGCTCGGCTATCTCGTCG/GGCCGTAGGCGTTGAACT
*sven_6836* forward/reverse	GTACGGAGATGCTCGACCTG/GTTGGAGTTCATGGCGTTCG
*sven_6837* forward/reverse	CTCGAGATGCTCGACACCC/GTTGATGACGAGGCTGCACG
*sven_7329* forward/reverse	CTCGACTTCGACCTCATCGC/GACGTTCACCTTGGTCATGC
*sven_7344* forward/reverse	CGCATCGCCATCAATGGTTT/GCTCGGTCAGGTCGTTGAC
*sven_5969* forward/reverse	ACAGTTCGTCATGTGGGACC/CGGGTGATGTCGGAGCAGA
*sven_5972* forward/reverse	CCTGCTCGCCTCGGACAT/CAGCCATTCGCCGTTGTC
*sven_6001* forward/reverse	TGGACCTGGCGGCGATTTC/ATCCTAGCATTGGCTTGACAGCCTG
*sven_6002* forward/reverse	TCGTCAGCTACGGCGTCCAG/GGGGTTCTCGAAGGTGAAGTCG

### RNA-Seq and data analysis

RNA-Seq was performed by Beijing Genomics Institute (BGI) Sequencing, as described (16, 29). The quality of total RNA was evaluated by NanoDrop 1000 (Thermo), and a cDNA library was constructed according to the manufacturer’s instructions (Illumina). rRNA in total RNA was depleted by the Ribo-zero rRNA Removal solution (Illumina), cDNA was synthesized using random primers and SuperScript II (Invitrogen), and the ends of the cDNA fragments were paired and then sequenced by HiSeq2000 (Illumina). The expression level of each gene was standardized by the number of FPKM. The cut-off value for assaying gene transcriptional activity was determined based on a 95% confidence interval for all FPKM values of each gene. The value for a specific gene was calculated as the log_2_ ratio of the expression level in FPKM of the gene in the mutant relative to that in the wild-type strain ISP5230. Fold changes of 2 were used as differential expression thresholds. The RNA-Seq data were deposited in NCBI (accession number PRJNA1196022).

### Determination of glucose content

The glucose assay was performed using the O-toluidine assay kit (Beyotime Biotechnology). Briefly, *Streptomyces* strains were inoculated onto N-Evans-CA agar covered with cellophane for designated times. The growth agar was separated from the cellophane, homogenized, and then extracted with an equal volume of ddH_2_O for 4–6 h. After centrifugation at 8,000 rpm for 10 min, the supernatants were collected, and 5 µL samples were used to react with 185 µL O-toluidine reagent at 95°C for 10 min, and then the mixture was transferred to ice-chilled water for 10 min. The absorbance was measured at 630 nm. The standard curve of glucose was prepared using 0, 5, 10, 20, 50, 100, 200, 400, 800, 1,200, 1,600, and 2,000 mg/L of glucose, and the glucose concentration of the samples was calculated according to the standard curves. The glucose concentration extracted from the blank (control) medium was set to 100%, and the glucose concentration of the media obtained at designated times was divided by the glucose concentration of the blank medium to determine the content (percentage) of remaining glucose in the medium.

### Detection of antibiotic production

CHL was detected by HPLC analysis. Briefly, equal numbers of spores were inoculated into N-Evans-CA liquid medium and harvested at indicated times. Samples were prepared and tested essentially as described (30). Known concentrations of CHL (0.01 µg/mL, 0.1 µg/mL, 1.0 µg/mL, 10 µg/mL, 100 µg/mL, and 1,000 µg/mL) were analyzed by HPLC, and the UV values were used to generate a standard curve to calibrate the level of CHL in the samples.

## Supplementary Material

Reviewer comments

## Data Availability

Genomic DNA of *S. venezuelae* was extracted by a commercial genomic DNA extraction kit (Tiangen, China) and then sequenced on the Nanopore PromethION platform (Oxford Nanopore Technologies) and Illumina NovaSeq platform by TSINGKE Technologies. The genome sequences were assembled using Unicycler (v0.4.9) and were deposited in NCBI (accession number PRJNA1252862).

## SUPPLEMENTAL MATERIAL

Supplemental material spectrum.00318-25-s0001.pdf Fig. S1 to S11; Tables S1 to S10.

OPEN PEER REVIEW reviewer-comments.pdf An accounting of the reviewer comments and feedback.

ASM does not own the copyrights to Supplemental Material that may be linked to, or accessed through, an article. The authors have granted ASM a non-exclusive, world-wide license to publish the Supplemental Material files. Please contact the corresponding author directly for reuse.
