# Supplementary material for: Impact of the alkaline volatile trimethylamine on the physiology of Streptomyces venezuelae: an integrated transcriptomic and metabolomics study
Source: Microbiol Spectr. 2025 May 27;13(7):e00318-25. doi: 10.1128/spectrum.00318-25 (PMC12211087; doi:10.1128/spectrum.00318-25)
Supplement: Supplemental material — Fig. S1 to S11; Tables S1 to S10. [file spectrum.00318-25-s0001.pdf]

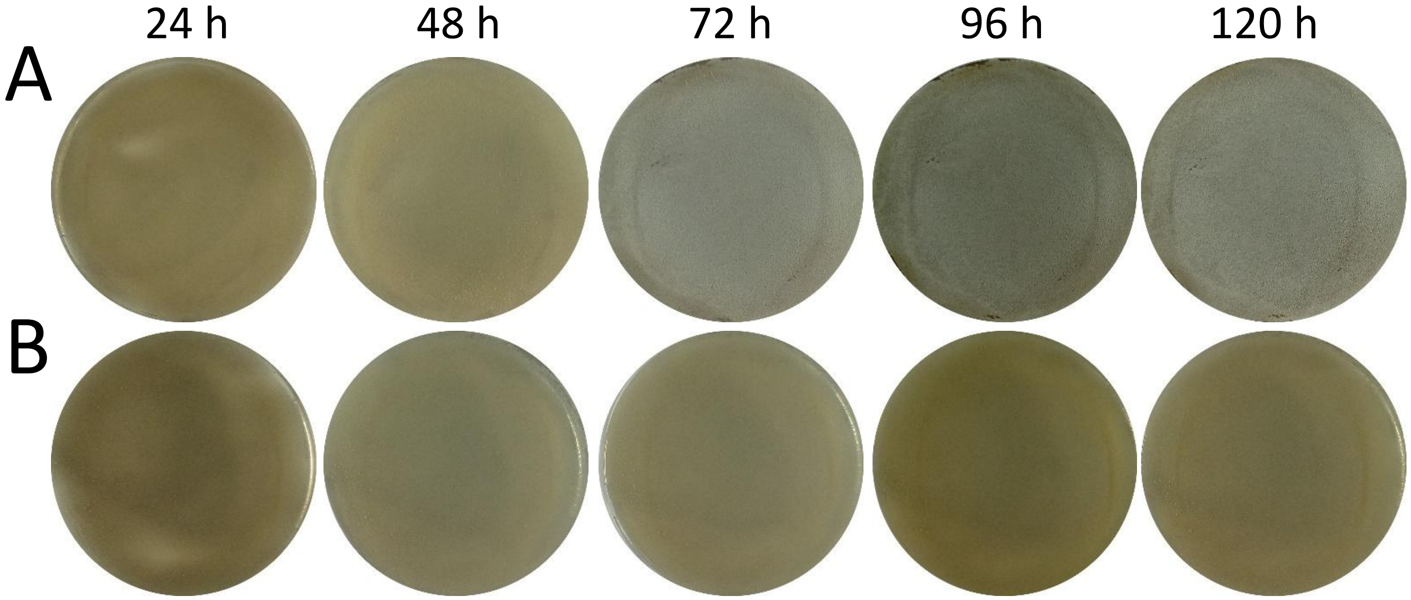

Fig. S1. Phenotypes of the *S. venezuelae* wild-type strain ISP5230 (A) and the mutant strain MU-1 (B). ISP5320 (WT) and MU-1 were grown on solid N-Evans-CA in separate plates for the indicated times before photographing. The bald phenotype of MU-1 is indicated by the lack of light greenish-grey spores at the later growth stages.

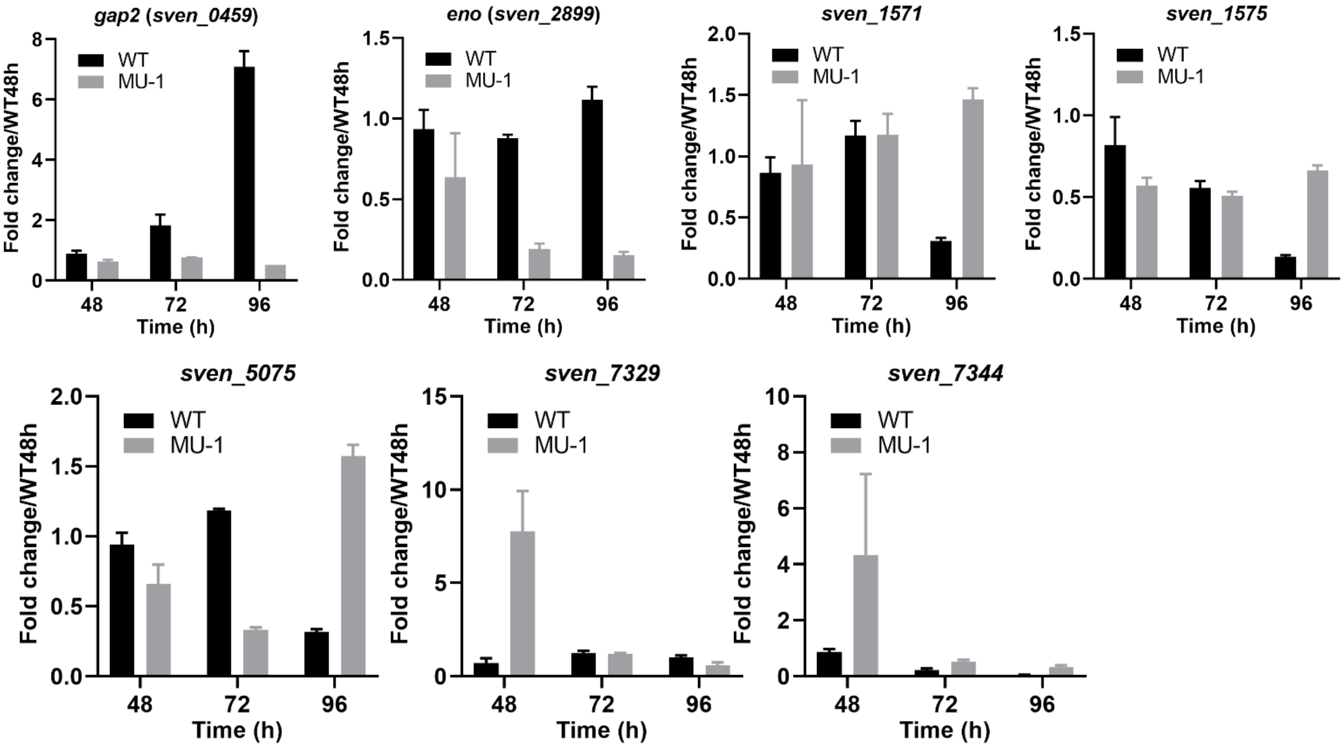

Fig. S2. Transcriptional analysis of genes involved in glycolysis in strain MU-1. ISP5230 (WT) and MU-1 were grown on solid N-Evans-CA medium, and RNA samples were isolated at the indicated times. Gene expression was measured by real-time PCR, and expression of *hrdB,* encoding the major *Streptomyces* sigma factor, was used as an internal control. The y-axis shows the fold change in expression level in ISP5230 (black bars) and MU-1 (grey bars) over the level of each gene in ISP5230 at 48 h, at each time point, with the expression level of each gene in one set of ISP5230 samples at 48 h arbitrarily set to one. Results are the means (±SD) of triplet biological experiments.

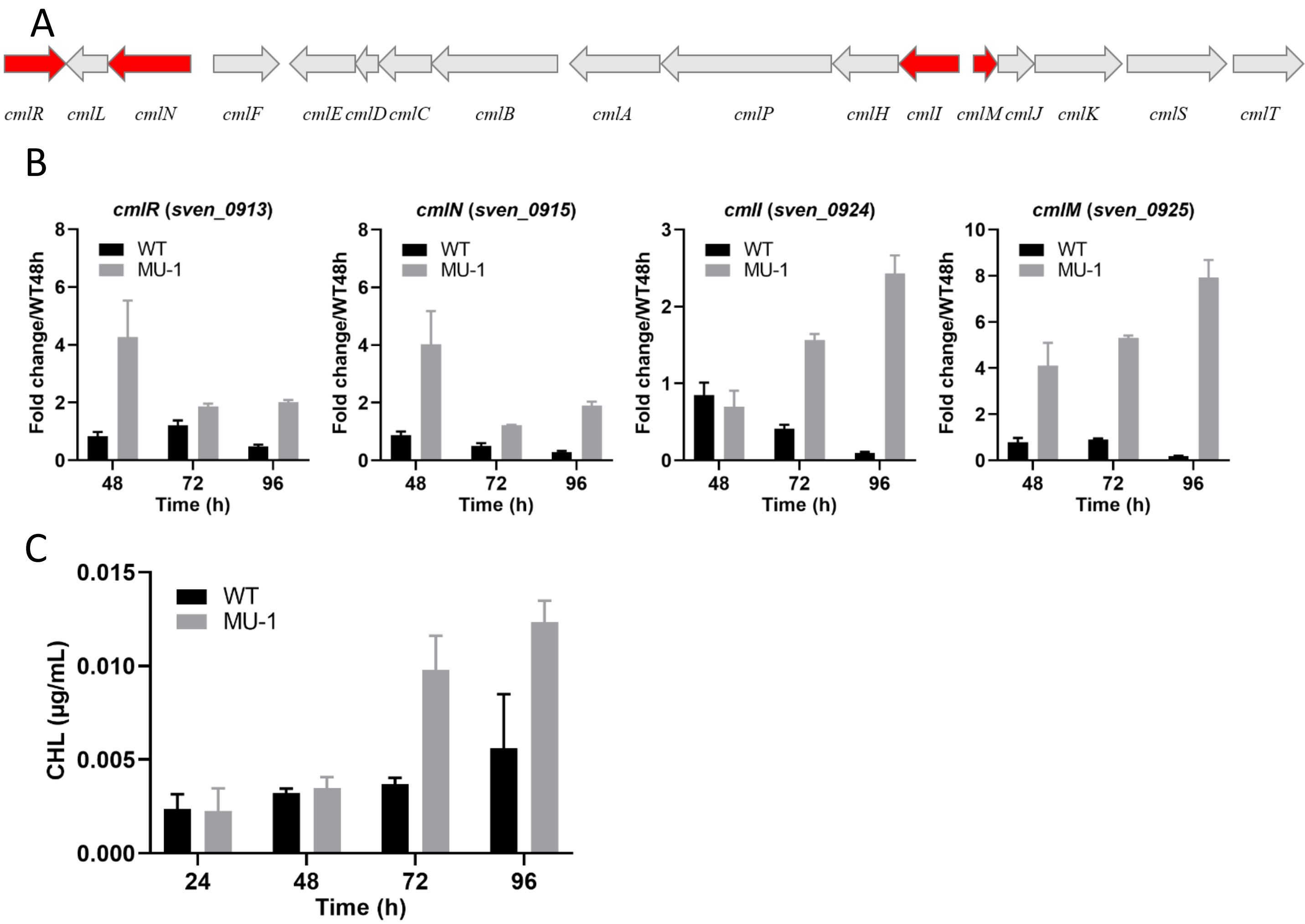

Fig. S3. Transcriptional analysis of antibiotic biosynthesis genes in strain MU-1 and production of CHL. (A) Schematic representation of the *cml* gene cluster. Genes in red were analyzed in (B). (B) Transcriptional analysis of *cml* genes. (C) Production of CHL in strain MU-1 and WT. For (B), ISP5230 (WT) and MU-1 were grown on solid N-Evans-CA medium, and RNA samples were isolated at the indicated times. Gene expression was examined as described in the legend for Figure S2. Results are the means (±SD) of triplet biological experiments.

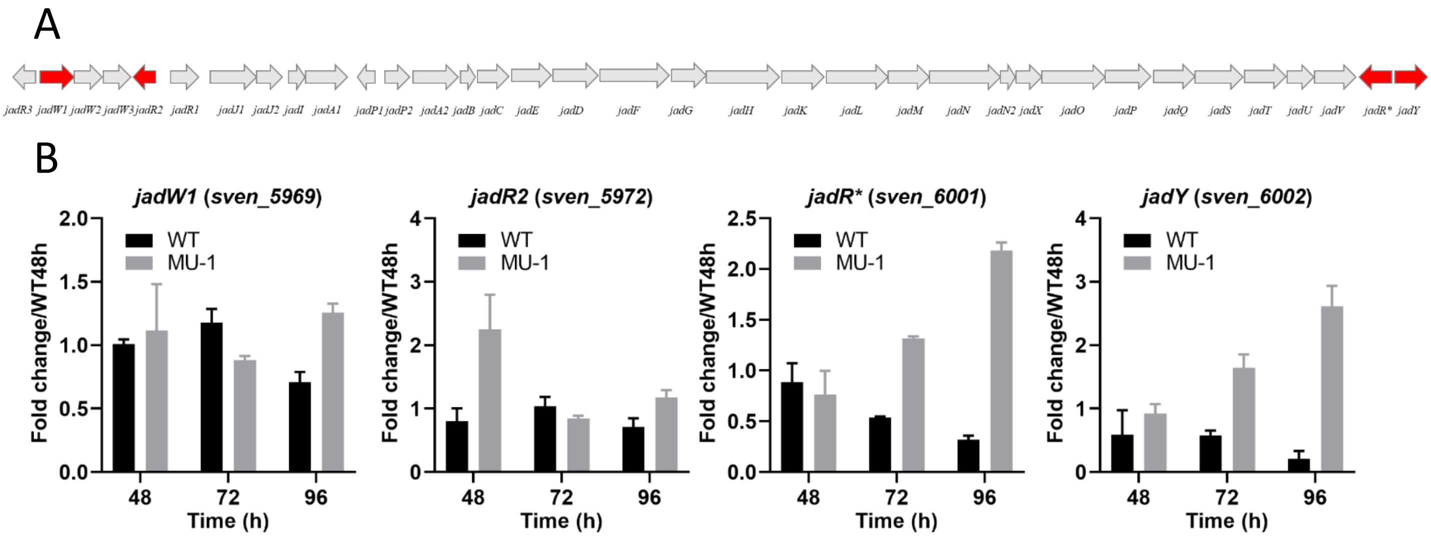

Fig. S4. Transcriptional analysis of antibiotic biosynthesis genes in strain MU-1. (A) Schematic representation of the *jad* gene cluster. Genes in red were analyzed in (B). (B) Transcriptional analysis of *jad* genes. For (B), ISP5230 (WT) and MU-1 were grown on solid N-Evans-CA medium, and RNA samples were isolated at the indicated times. Gene expression was examined as described in the legend for Figure S2. Results are the means (±SD) of triplet biological experiments.

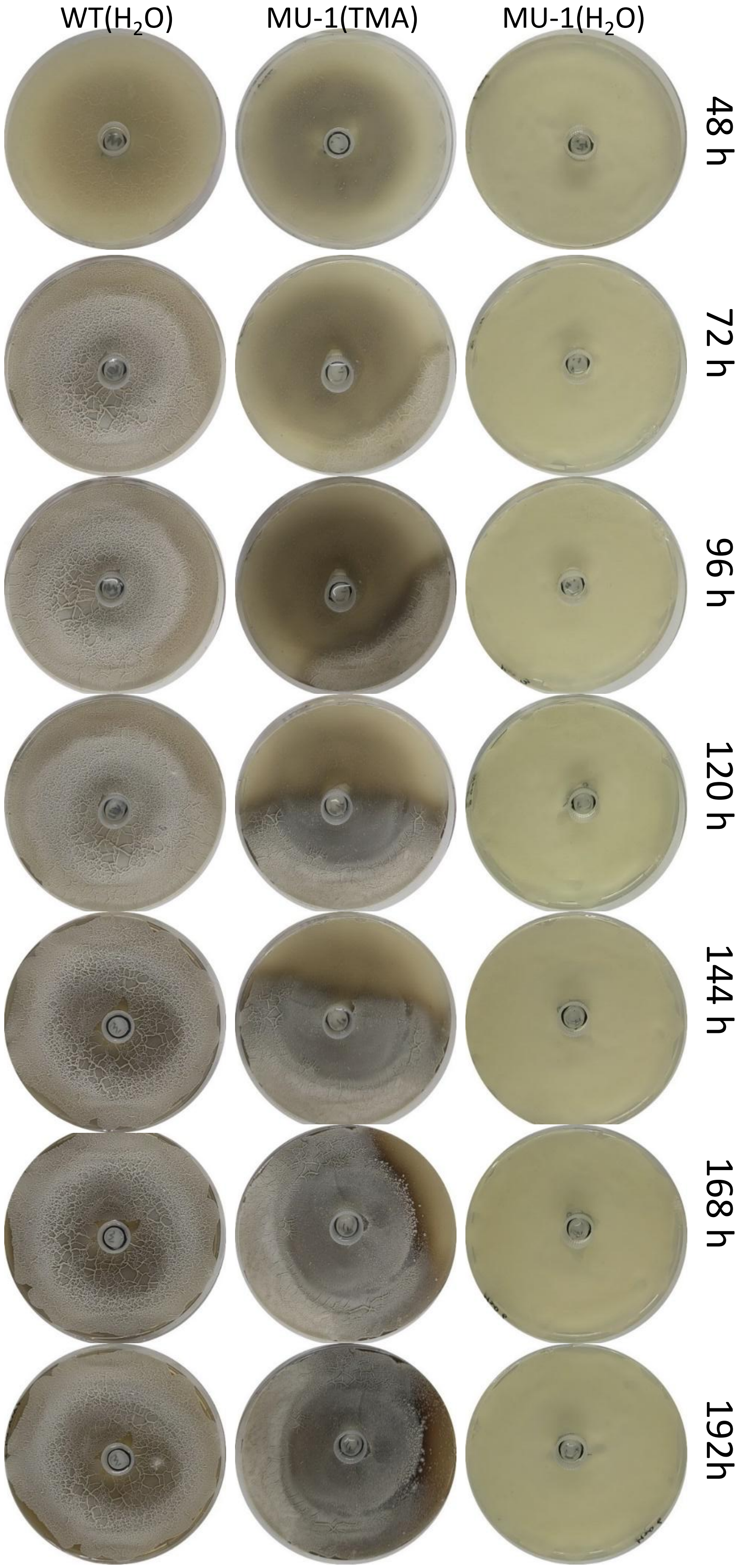

Fig. S5. Effects of TMA exposure on the growth of strain MU-1. ISP5230 (WT) and MU-1 were grown at 30° C on solid N-Evans-CA medium. Water or TMA was added to the container in the center of each plate, and plates were incubated for the indicated times.

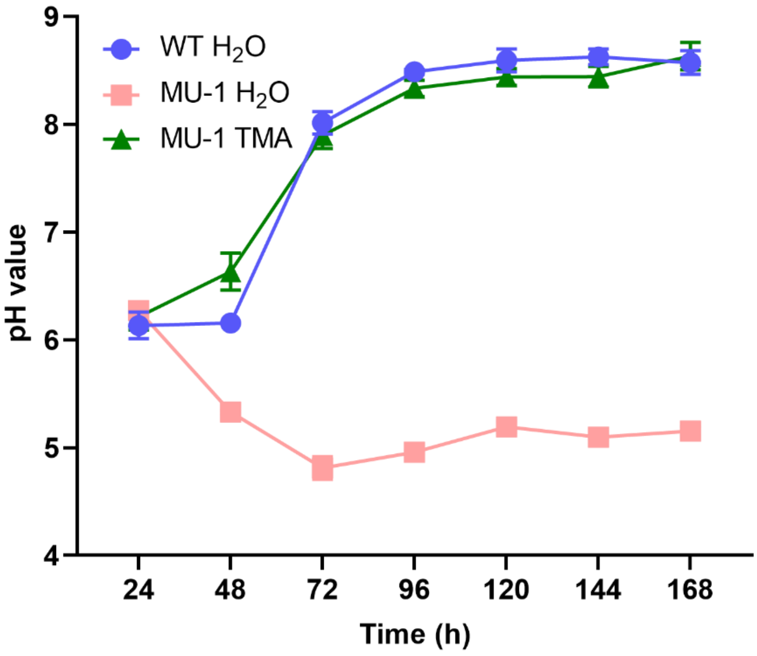

Fig. S6. Temporal pH values of the growth medium of strain MU-1 exposed to TMA. ISP5230 (WT) and MU-1 were grown at 30° C on solid N-Evans-CA medium. Water or TMA was added to the container in the center of each plate, and plates were incubated for the indicated times.

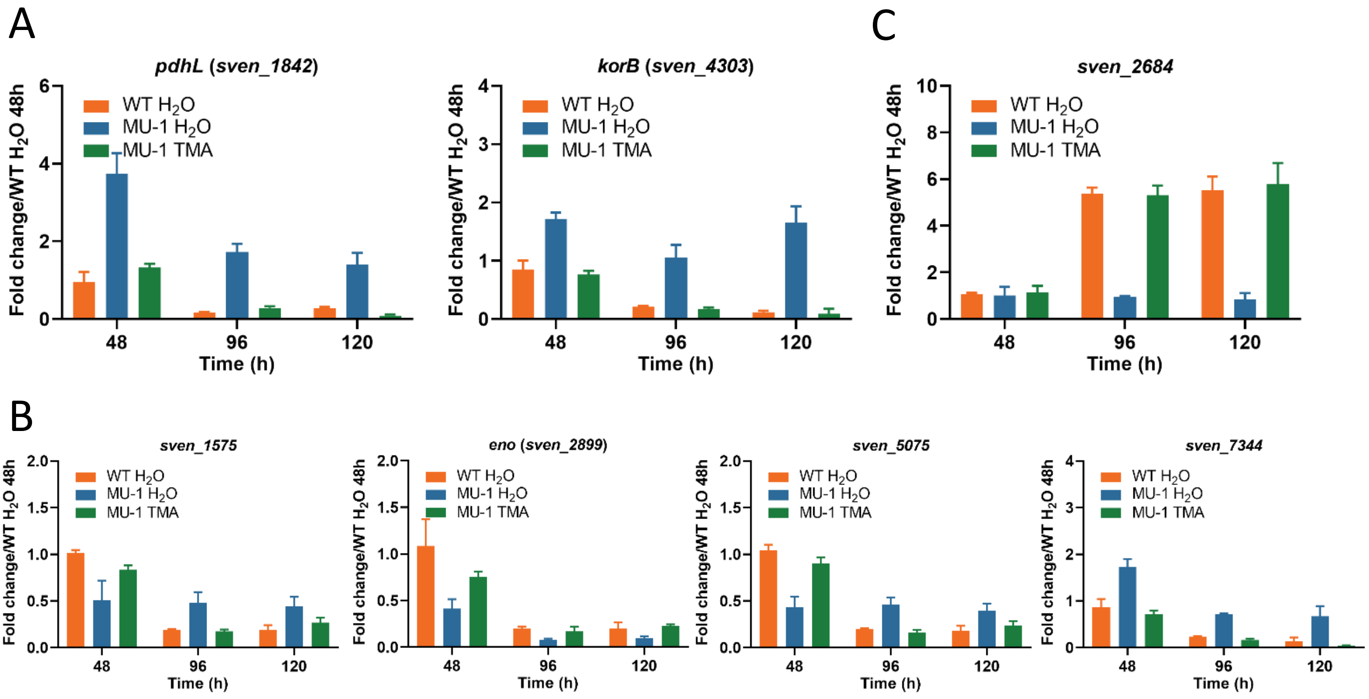

Fig. S7. Transcriptional analysis of carbon metabolism genes in strain MU-1 exposed to TMA. Transcriptional analysis of genes involved in (A) the TCA cycle, (B) glycolysis, and (C) pyruvate metabolism in strain MU-1 exposed to TMA or water and ISP5230 (WT) exposed to water. Gene expression was examined as described in the legend for Figure S2. Results are the means (±SD) of triplet biological experiments.

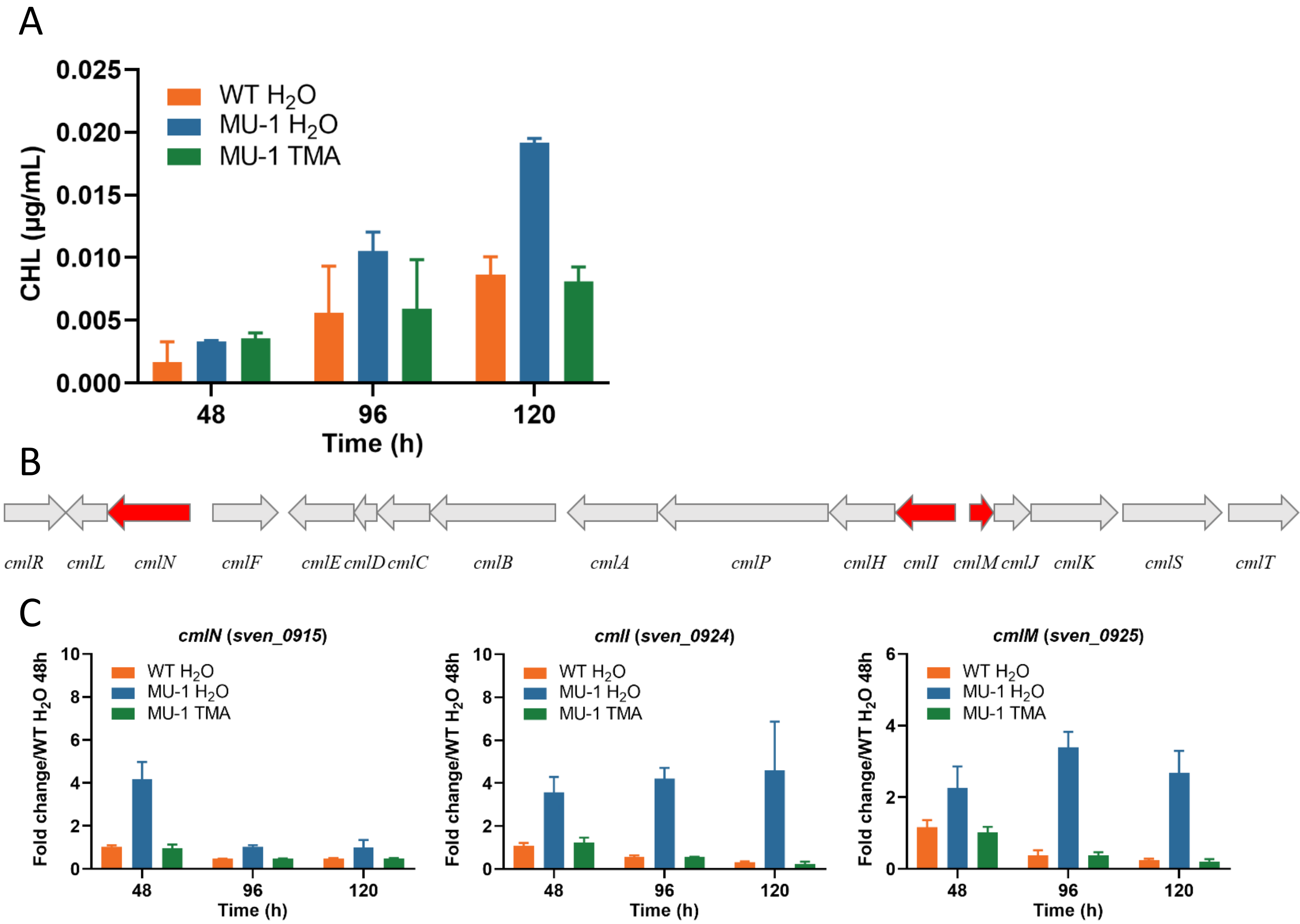

Fig. S8. TMA exposure complements the expression of antibiotic genes. (A) Production of CHL in strain MU-1 exposed to TMA. (B) Schematic representation of the *cml* gene cluster. Genes in red were analyzed in (C). (C) Transcriptional analysis of *cml jad* genes in strain MU-1 exposed to TMA or water and ISP5230 (WT) exposed to water. Gene expression was examined essentially as described in the legend for Figure S2 with the fold change determined relative to the expression level of each gene in one set of WT H_2O samples at 48 h, which is arbitrarily set to one. Results are the means (±SD) of triplet biological experiments.

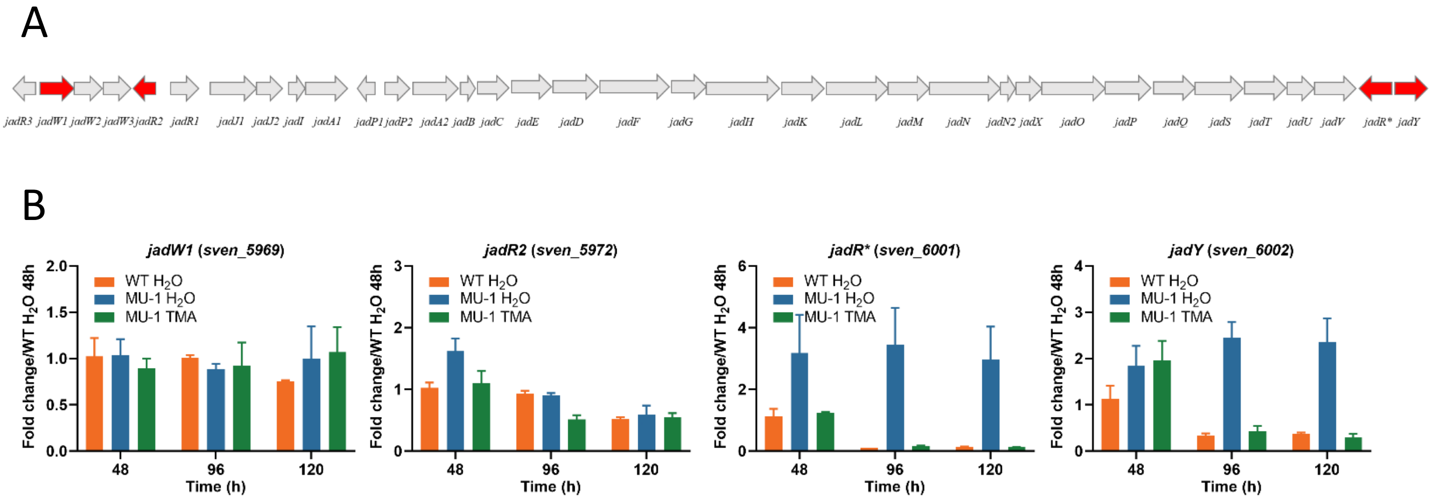

Fig. S9. TMA exposure complements the expression of antibiotic genes. (A) Schematic representation of the *jad* gene cluster. Genes in red were analyzed in (B). (B) Transcriptional analysis of *jad* genes in strain MU-1 exposed to TMA or water and ISP5230 (WT) exposed to water. Gene expression was examined essentially as described in the legend for Figure S2 with the fold change determined relative to the expression level of each gene in one set of WT H_2O samples at 48 h, which is arbitrarily set to one. Results are the means (±SD) of triplet biological experiments.

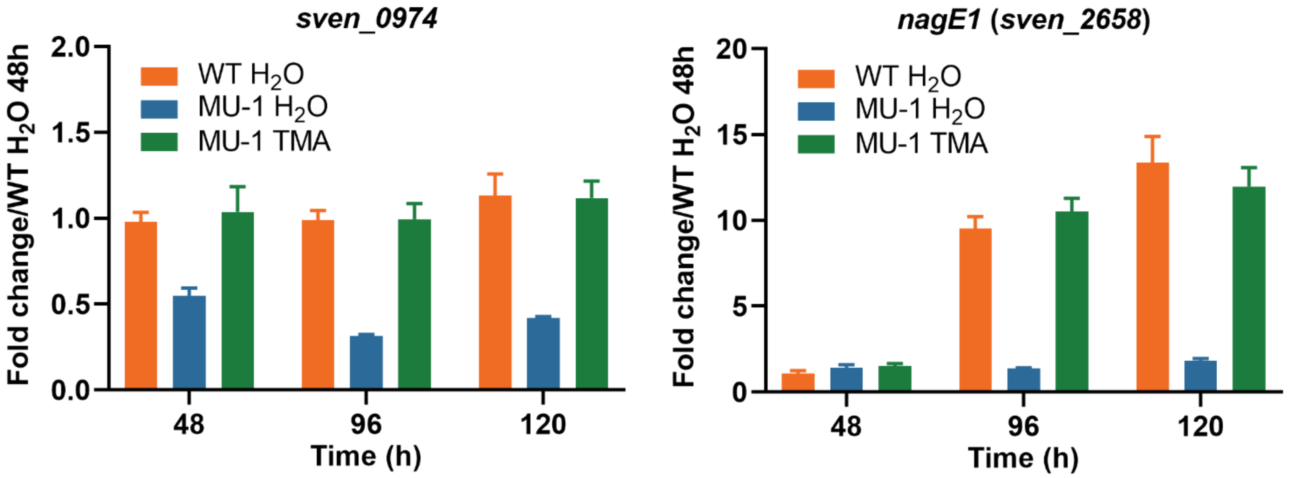

Fig. S10. Transcriptional analysis of transporter genes in strain MU-1 exposed to TMA. Gene expression was examined as described in the legend for Figure S7. Results are the means (±SD) of triplet biological experiments.

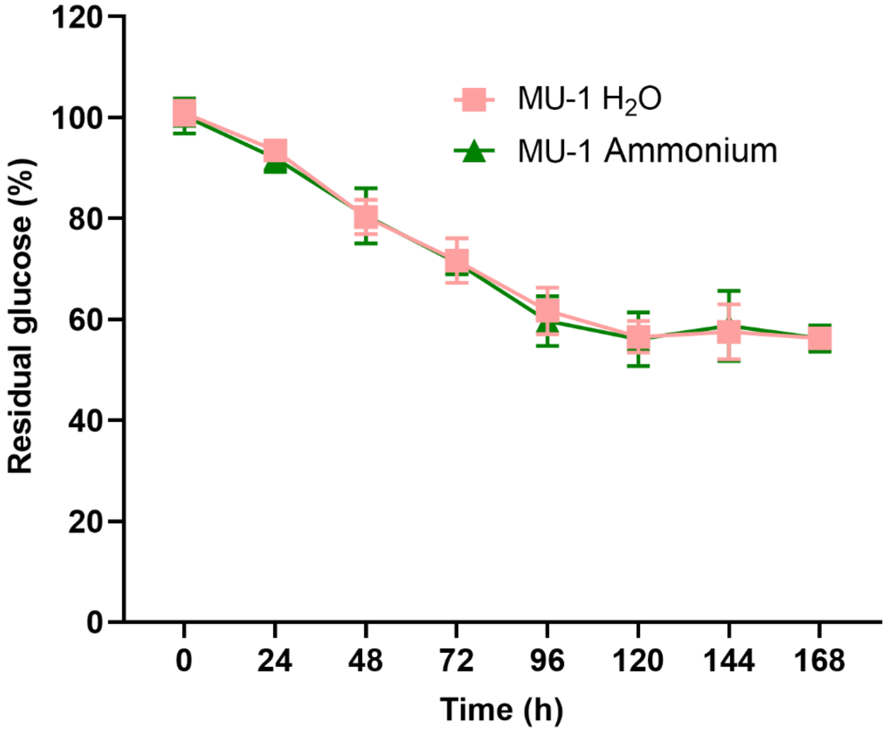

Fig. S11. Temporal glucose content curves of MU-1 exposed to ammonium. Cultures of *S. venezuelae* strain MU-1 were exposed to ammonium or water during growth at 30° C on solid N-Evans-CA medium. Results are the means (±SD) of triplet biological experiments.

Table S1 Differentially expressed developmental genes in MU-1

Gene ID	Length	Annotation and function	Fold change-48 h	Fold change-96 h	Change
SVEN_1269	930	*chpC*	ND	**-3.48** (1888.63/169.22)	Down
SVEN_1270	234	*chpH*	ND	**-4.11** (11987.44/697.04)	Down
SVEN_1437	246	*chpE*	ND	**-3.47** (3293.54/297.49)	Down
SVEN_4645	240	*chpF*	ND	**-7.92** (2722.72/11.25)	Down
SVEN_4651	276	*chpG*	ND	**-4.82** (9210.76/326.26)	Down
SVEN_4628	408	*rdlA*	ND	**-7.18** (9163.44/63.05)	Down
SVEN_4631	411	*rdlB*	ND	**-7.40** (10573.46/62.45)	Down
SVEN_4632	399	*rdlA2*	ND	**-7.59** (1039.59/5.41)	Down
SVEN_6482	2628	*ramC*	ND	2.67 (2.03/12.96)	Up
SVEN_6481	129	*ramS*	ND	ND	/
SVEN_6480	2094	*ramA*	ND	2.11 (1.61/6.98)	Up
SVEN_6479	1872	*ramB*	ND	1.55 (11.61/34.19)	Up
SVEN_6478	615	*ramR*	ND	**3.73** (13.79/183.35)	Up
SVEN_1089	501	*bldD*	ND	ND	/
SVEN_2580	996	*bldH*	ND	ND	/
SVEN_3185	786	*bldN*	ND	**-4.71** (1582.74/60.64)	Down
SVEN_3319	354	*bldG*	ND	ND	/
SVEN_3846	207	*bldC*	ND	ND	/
SVEN_4453	612	*bldM*	ND	**-2.77** (2120.93/310)	Down
SVEN_5382	324	*bldB*	ND	ND	/
SVEN_1578	984	*whiA*	ND	ND	/
SVEN_2776	264	*whiB*	ND	-1.83 (742.72/205.43)	Down
SVEN_4452	393	*whiD*	ND	**-3.98** (828.89/52.43)	Down
SVEN_5300	777	*whiG*	ND	-1.92 (186.67/49.4)	Down
SVEN_5498	888	*whiH*	ND	**-6.04** (2255.44/34.19)	Down
SVEN_5827	666	*whiI*	ND	**-5.39** (1874.31/44.62)	Down
SVEN_6792	333	*whiEVII*	ND	**-12.11** (442.19/0.1) *	Down
SVEN_6793	480	*whiEORFVI*	ND	**-12.73** (682.42/0.1) *	Down
SVEN_6794	261	*whiEORFV*	ND	**-12.09** (436.03/0.1) *	Down
SVEN_6795	1248	*whiEORFIV*	ND	**-9.43** (491.16/0.71)	Down
SVEN_6796	1269	*whiEORFIII*	ND	**-12.74** (687.13/0.1) *	Down
SVEN_6797	471	*whiEORFII*	ND	**-12.55** (599.7/0.1) *	Down
SVEN_6798	1122	*whiEORFI*	ND	**-9.38** (1062.83/1.6)	Down
SVEN_6799	1662	*whiEORFVIII*	1.21 (10.36/24.03)	**-2.35** (93.48/18.34)	Down

The values in bold indicate those expression values above the cutoff value. The value for a specific gene was calculated as the $\log_2$ ratio of the expression level in FPKM (the number of fragments per kilobase per million) of the gene in the mutant relative to that in the wild-type strain ISP5230, as determined by RNA-Seq. ND, no change detected. *, the expression level of the gene in one strain was only minimal.

Table S2 Differentially expressed genes involved in TCA cycle in MU-1

Gene ID	Length	Annotation and function	Fold change -48 h	Fold change -96 h	Change
SVEN_0436	2226	*Idh*, isocitrate dehydrogenase	ND	1.43 (224.68/607.12)	Up
SVEN_1843	1791	*sucB*, oxoglutarate dehydrogenase	ND	**2.36** (691.04/3557.13)	Up
SVEN_2535	1302	*citA*, citrate synthase	ND	**2.92** (225.42/1707.07)	Up
SVEN_4205	1101	*citA*, citrate synthase	1.73 (636.1/2113.47)	**2.62** (87.48/537.22)	Up
SVEN_4532	1755	*dhsA*, succinate dehydrogenase flavoprotein subunit	-1.77 (516.49/151.36)	-1.00 (388.22/193.88)	Down
SVEN_4533	480	succinate dehydrogenase	-1.65 (395.36/126.15)	-1.12 (296.11/136.67)	Down
SVEN_4534	381	Succinate dehydrogenase cytochrome subunit	-2.12 (469.74/108.05)	-1.45 (283.54/103.5)	Down
SVEN_4708	1410	*fumC*, fumarate hydratase	ND	**3.48** (55.5/617.59)	Up
SVEN_4713	1671	*fumB*, fumarate hydratase	ND	1.52 (190.79/548.07)	Up
SVEN_4753	1911	*sdhA2*, succinate dehydrogenase	ND	-2.52 (95.87/16.73)	Down
SVEN_4966	3801	*kgd*, alpha-ketoglutarate decarboxylase	ND	1.78 (917.11/3150.28)	Up
SVEN_5812	2733	*sacA*, aconitate hydratase	ND	1.01 (254.35/511.23)	Up
SVEN_6835	627	succinate dehydrogenase	ND	**4.34** (15.92/321.33)	Up
SVEN_6836	1947	succinate dehydrogenase	ND	**4.12** (16.36/285.92)	Up
SVEN_6837	750	succinate dehydrogenase	ND	**4.19** (27.78/506.81)	Up

The values in bold indicate those expression values above the cutoff value. The value for a specific gene was calculated as the $\log_2$ ratio of the expression level in

FPKM (the number of fragments per kilobase per million) of the gene in the mutant relative to that in the wild-type strain ISP5230, as determined by RNA-Seq. ND, no change detected.

Table S3 Differentially expressed genes involved in glycolysis in MU-1

Gene ID	Length	Annotation and function	Fold change -48 h	Fold change -96 h	Change
SVEN_0459	1386	glyceraldehyde-3-phosphate dehydrogenase	ND	**-2.53** (327.27/56.61)	Down
SVEN_1571	1671	Glucose-6-phosphate isomerase	ND	1.27 (90.89/219.33)	Up
SVEN_1575	1211	phosphoglycerate kinase	ND	1.63 (345.59/1068.31)	Up
SVEN_2899	1287	*eno*, enolase	-1.69 (1155.09/358.74)	1.42 (621.38/231.49)	Up
SVEN_3052	2010	Fructose-specific PTS system	ND	**5.59** (19.19/924.71)	Up
SVEN_3053	954	phosphofructokinase	ND	**8.10** (3.02/830.07)	Up
SVEN_3054	762	Repressor of fructose operon	ND	**7.61** (5.84/1134.57)	Up
SVEN_5075	1431	pyruvate kinase	ND	1.46 (275.07/755.39)	Up
SVEN_7329	837	fructose-bisphosphate aldolase	2.10 (6.22/26.72)	1.84 (7.87/27.68)	Up
SVEN_7344	996	glyceraldehyde-3-phosphate dehydrogenase	1.25 (14033.5/33345.5)	**7.49** (100.65/18080.45)	Up

The values in bold indicate those expression values above the cutoff value. The value for a specific gene was calculated as the $\log_2$ ratio of the expression level in FPKM (the number of fragments per kilobase per million) of the gene in the mutant relative to that in the wild-type strain ISP5230, as determined by RNA-Seq. ND, no change detected.

Table S4 Differentially expressed genes associated with pyruvate metabolism in MU-1

Gene ID	Length	Annotation and function	Fold change -48 h	Fold change -96 h	Change
SVEN_1845	2706	*aceE1*, pyruvate dehydrogenase	ND	**-3.84** (888.6/61.93)	Down
SVEN_3586	981	*bkdB1*, pyruvate dehydrogenase	ND	**-2.72** (282.23/42.65)	Down
SVEN_3587	1179	*bkdA1*, pyruvate dehydrogenase	ND	**-2.42** (288.77/53.79)	Down
SVEN_5943	1743	*poxB*, pyruvate dehydrogenase	ND	**-4.42** (308.47/14.45)	Down
SVEN_4658	1824	*pepck*, phosphoenolpyruvate carboxykinase	ND	**-6.82** (1125.05/9.97)	Down
SVEN_2684	1146	hydroxyphenylpyruvate dioxygenase	ND	**-4.19** (974.98/53.14)	Down

The values in bold indicate those expression values above the cutoff value. The value for a specific gene was calculated as the log_2 ratio of the expression level in FPKM (the number of fragments per kilobase per million) of the gene in the mutant relative to that in the wild-type strain ISP5230, as determined by RNA-Seq. ND, no change detected.

Table S5 Differentially expressed *cml* genes in MU-1

Gene ID	Length (bp)	Annotation and function	Fold change-48 h	Fold change-96 h	Change
SVEN_0913	1035	*cmlR*	ND	1.61 (17.22/52.43)	Up
SVEN_0914	720	*cmlL*	1.71 (13.78/45.11)	**3.15** (7.28/64.24)	Up
SVEN_0915	1419	*cmlN*	1.16 (21.64/48.38)	**2.93** (10.73/81.87)	Up
SVEN_0916	1212	*cmlF*	ND	ND	Up
SVEN_0917	1263	*cmlE*	1.13 (91.61/200.78)	**3.25** (48.7/461.95)	Up
SVEN_0918	312	*cmlD*	ND	**10.24** (0.1/121.17)*	Up
SVEN_0919	987	*cmlC*	ND	**7.35** (2.18/356.38)	Up
SVEN_0920	2061	*cmlB*	1.22 (77.76/181.06)	**7.54** (1.64/306.08)	Up
SVEN_0921	1599	*cmlA*	ND	**6.07** (4.72/317.73)	Up
SVEN_0922	2892	*cmlP*	ND	**8.61** (1.38/539.09)	
SVEN_0923	1179	*cmlH*	ND	**6.78** (4.77/525.05)	Up
SVEN_0924	1020	*cmlI*	ND	**7.78** (4.2/919.97)	Up
SVEN_0925	327	*cmlM*	ND	**4.61** (19.98/488.93)	Up
SVEN_0926	699	*cmlJ*	ND	**8.39** (2.12/723.8)	Up
SVEN_0927	1446	*cmlK*	ND	**7.62** (2.87/562.83)	Up
SVEN_0928	1716	*cmlS*	ND	**8.43** (2.39/823.58)	Up
SVEN_0929	981	*cmlT*	1.01 (100.72/203.42)	**8.17** (1.46/419.99)	Up

The values in bold indicate those expression values above the cutoff value. The value for a specific gene was calculated as the $\log_2$ ratio of the expression level in FPKM (the number of fragments per kilobase per million) of the gene in the mutant relative to that in the wild-type strain ISP5230, as determined by RNA-Seq. ND, no change detected. *, the expression level of the gene in one strain was only minimal.

Table S6 Differentially expressed *jad* genes in MU-1

Gene ID	Length	Annotation and function	Fold change-48 h	Fold change-96 h	Change
SVEN_5968	681	*jadR3*	-1.69 (53.27/16.47)	-1.69 (163.25/50.49)	Down
SVEN_5969	951	*jadW1*	ND	ND	/
SVEN_5970	948	*jadW2*	1.34 (13.86/35.12)	2.29 (5.32/26)	Up
SVEN_5971	765	*jadW3*	ND	1.30 (41.7/103.01)	Up
SVEN_5972	687	*jadR2*	ND	ND	/
SVEN_5973	780	*jadR1*	ND	ND	/
SVEN_5974	1181	*jadJ*	-2.12 (215.26/49.59)	ND	/
SVEN_5975	579	*jadJ2*	ND	ND	/
SVEN_5976	330	*jadI*	ND	ND	/
SVEN_5977	957	*jadA1*	ND	ND	/
SVEN_5980	540	*jadA2*	ND	ND	Up
SVEN_5981	1215	*jadB*	1.24 (28.57/67.64)	**4.04** (5.77/94.59)	Up
SVEN_5982	270	*jadC*	ND	ND	Up
SVEN_5983	795	*jadE*	ND	**3.39** (22.19/242.97)	Up
SVEN_5984	936	*jadD*	ND	1.22 (283.61/659.33)	Up
SVEN_5985	1539	*jadF*	ND	**2.46** (920.06/5075.64)	Up
SVEN_5986	678	*jadG*	ND	1.73 (551.46/1835.28)	Up
SVEN_5987	1635	*jadH*	ND	**3.33** (588.35/5919.73)	Up
SVEN_5988	921	*jadK*	ND	**3.04** (259.77/2142.24)	Up
SVEN_5989	1377	*jadL*	ND	**3.12** (163.27/1420.09)	Up
SVEN_5990	789	*jadM*	ND	**4.47** (65.5/1456.21)	Up
SVEN_5991	1575	*jadN*	ND	**2.77** (104.74/716.63)	Up
SVEN_5992	231	*jadN2*	ND	ND	/
SVEN_5993	519	*jadX*	ND	**2.96** (49.36/383.89)	Up
SVEN_5994	1416	*jadO*	ND	**2.72** (53.3/350.59)	Up
SVEN_5995	1002	*jadP*	ND	**3.65** (39.28/493.58)	Up
SVEN_5996	870	*jadQ*	ND	**3.71** (23.44/306.36)	Up
SVEN_5997	1194	*jadS*	ND	**3.57** (26.48/316.46)	Up
SVEN_5998	969	*jadT*	ND	**2.59** (20.02/120.3)	Up
SVEN_5999	609	*jadU*	ND	**3.35** (16.49/167.9)	Up
SVEN_6000	1020	*jadV*	ND	**3.59** (42.7/516.82)	Up
SVEN_6001	615	*jadR**	ND	**3.14** (47.64/420.43)	Up
SVEN_6002	612	*jadY*	ND	**4.61** (3.78/92.21)	Up

The values in bold indicate those expression values above the cutoff value. The

value for a specific gene was calculated as the log_2 ratio of the expression level in FPKM (the number of fragments per kilobase per million) of the gene in the mutant relative to that in the wild-type strain ISP5230, as determined by RNA-Seq. ND, no change detected.

Table S7 Differentially expressed genes potentially encoding venezuelin in MU-1

Gene ID	Length	Annotation and function	Fold change-48 h	Fold change-96 h	Change
SVEN_0620	2853	Membrane protein	ND	**-6.61** (436.07/4.46)	Down
SVEN_0621	156	Lantibiotic precursor	ND	**-7.49** (2147/11.93)	Down
SVEN_0622	1129	ABC transporter ATP-binding protein	ND	**-6.14** (281.22/3.98)	Down
SVEN_0623	912	Permease protein	ND	**-4.14** (88.87/5.03)	Down
SVEN_0624	1308	Xylose repressor	ND	-1.31 (42.09/16.93)	Down
SVEN_0625	1305	Secreted sugar binding protein	ND	ND	/
SVEN_0626	993	Permease protein	ND	**-3.83** (12.98/0.91)	Down
SVEN_0627	882	Permease protein	ND	ND	/
SVEN_0628	1482	Beta-galactosidase	ND	ND	/
SVEN_0629	1020	Transcriptional regulator	ND	ND	/

The values in bold indicate those expression values above the cutoff value. The value for a specific gene was calculated as the $\log_2$ ratio of the expression level in FPKM (the number of fragments per kilobase per million) of the gene in the mutant relative to that in the wild-type strain ISP5230, as determined by RNA-Seq. ND, no change detected.

Table S8 Differentially expressed genes potentially encoding geosmin in MU-1

Gene ID	Length	Annotation and function	Fold change-48 h	Fold change-96 h	Change
SVEN_0265	1470	phosphatase	ND	**-2.62** (2385.26/388.17)	Down
SVEN_0266	1494	Regulatory protein	ND	ND	Down
SVEN_0267	1128	phosphatransferase	ND	-1.54 (8601.3/2948.18)	Down
SVEN_0268	1920	amidase	ND	-1.86 (365.33/100.77)	Down
SVEN_0269	2187	Hypothetical protein	ND	**-4.25** (306.51/16.09)	Down
SVEN_0270	366	Heat shock protein	ND	ND	/
SVEN_0271	501	Hypothetical protein	ND	-1.41 (569.09/214.64)	Down
SVEN_0272	2304	Hypothetical protein	ND	1.33(223.42/562.26)	Up

The values in bold indicate those expression values above the cutoff value. The value for a specific gene was calculated as the log_2 ratio of the expression level in FPKM (the number of fragments per kilobase per million) of the gene in the mutant relative to that in the wild-type strain ISP5230, as determined by RNA-Seq. ND, no change detected.

Table S9 Differentially expressed transporter genes in MU-1

Gene ID	Length	Annotation and function	Fold change -48 h	Fold change -96 h	Change
SVEN_0701	1221	Integral membrane Transporter	1.17 (2154.11/4856.1)	**9.07** (13.78/7397.94)	Up
SVEN_0702	795	Hypothetical protein	1.07 (1404.52/2959.68)	**5.29** (124.3/4853.48)	Up
SVEN_0790	2055	ABC transporter transmembrane subunit	1.06 (573.09/1197.82)	**5.01** (28.61/918.97)	Up
SVEN_0791	1851	ABC transporter ATP-binding protein	ND	**5.76** (24.97/1349.33)	Up
SVEN_0974	450	PTS glucose transporter subunit IIA	ND	-1.31 (212.58/85.7)	Down
SVEN_2658	1299	*nagE1*, PTS sugar transporter subunit IIA	ND	**-6.97** (343.54/2.73)	Down
SVEN_2659	1275	*nagE2*, PTS sugar transporter subunit IIC	ND	-2.82 (14.79/2.09)	Down
SVEN_3713	1509	cytochrome oxidase subunit I	ND	**6.14** (145.2/10222.2)	Up
SVEN_3714	1002	cytochrome oxidase subunit II	ND	**5.70** (118.5/6154.76)	Up
SVEN_3715	3591	ABC transporter	ND	**4.82** (63.5/1798.56)	Up
SVEN_4564	1284	Transport integral membrane protein	ND	**8.31** (11.42/3621.28)	Up
SVEN_3034	603	Hypothetical protein	1.70 (172.19/559.78)	**6.80** (12.84/1430.26)	Up
SVEN_3036	219	Hypothetical protein	1.08 (9111.88/19342.5)	**5.49** (2822.02/127356.56)	Up

The values in bold indicate those expression values above the cutoff value. The

value for a specific gene was calculated as the log_2 ratio of the expression level in FPKM (the number of fragments per kilobase per million) of the gene in the mutant relative to that in the wild-type strain ISP5230, as determined by RNA-Seq. ND, no change detected.

Table S10. Sequence changes in *S. venezuelae* MU-1

Genome position	ATCC10712	ISP5230	MU-1	Gene	Gene annotation	Mutation
100492-100861	gggtcgcacc ggtcgggtcg gccccccttgg cgatgacgtc cacgctgtac gggtcgtccc ccgaacccgc cgcgtaggcc gggccgctcc ccatcgccag cgcgccggc ggcggcgcc ggccccggc cgtcgccatg ccgcggatga cggatctgcg gtccatgcctc tggtcatttctg cccccaaagt aatgatcatga gaaagacatg acatacgaa cacgaccgg ggccggaac gcaagatcga ttcccgtggg ggaacctcac atgcgtgggg ggcatctcag acccgccgct ccgtccgccg actcggccac cg tttgtccagg cccctggttc accaggcgta	ggcaccggat cggacggtgg gggccgcga cggtcgtcgc ggcc	--	Upstream of SVEN_0101	Secreted hydrolase	Deletion mutation, at position -101 upstream of the translation start site of SVEN_0101.
1871868-1871869	--	--	cga	SVEN_1676	Glutamate synthase	Insertion mutation, V370
1985675-	+	+	--	SVEN_1780	response	Deletion

1986346					regulator	mutation
1986343-1987566	+	+	--	SVEN_1781	sensor kinase	Deletion mutation
2022497	a	a	g	NA	NA	Downstream of SVEN_1815
2022551	--	--	c	NA	NA	Downstream of SVEN_1815
2022510	--	--	c	NA	NA	Downstream of SVEN_1815
2022561	--	--	c	NA	NA	Downstream of SVEN_1815
2022471	g	g	t	NA	NA	Downstream of SVEN_1815
2022506 - 2022507	--	--	cgc	NA	NA	Downstream of SVEN_1815
2092778 - 2092781	gacc	gacc	ccag	SVEN_1881	Acyl transferase domain in polyketide synthase	Missense mutations, D325P, P326A
3022623	c	c	t	SVEN_r8	23S ribosomal RNA	Downstream of SVEN_2900
3022665	t	t	c	SVEN_r8	23S ribosomal RNA	Point mutation
3022726	a	a	c	SVEN_r8	23S ribosomal RNA	Point mutation
3022728-3022730	gtc	gtc	cct	SVEN_r8	23S ribosomal	Points mutations

					RNA	
3022734	t	t	g	SVEN_r8	23S ribosomal RNA	Point mutation
3022737-3022738	gt	gt	ag	SVEN_r8	23S ribosomal RNA	Point mutations
3022746	a	a	g	SVEN_r8	23S ribosomal RNA	Point mutation
3022787	a	a	c	SVEN_r8	23S ribosomal RNA	Point mutation
3022796-3022800	cgcct	cgcct	taatg	SVEN_r8	23S ribosomal RNA	Point mutations
3022802-3022803	at	at	gc	SVEN_r8	23S ribosomal RNA	Point mutations
4403746	a	a	c	SVEN_4066	hypothetical protein	Silent mutation
4406791	c	c	t	SVEN_4066	hypothetical protein	Silent mutation
4406830	c	c	a	SVEN_4066	hypothetical protein	Silent mutation
4406832-4406833	cg	cg	aa	SVEN_4066	hypothetical protein	Missense mutation, A1710E
4406836-4406839	gtac	gtac	cctg	SVEN_4066	hypothetical protein	Missense mutation, Y1712L
4406845	g	g	c	SVEN_4066	hypothetical protein	Silent mutation
4406848	g	g	c	SVEN_4066	hypothetical protein	Missense mutation, K1715N
4406851	g	g	c	SVEN_4066	hypothetical protein	Silent mutation
4406911	t	t	c	SVEN_4066	hypothetical protein	Silent mutation
4406932	g	g	c	SVEN_4066	hypothetical protein	Silent mutation
4406935	g	g	c	SVEN_4066	hypothetical protein	Silent mutation

4406974	g	g	c	SVEN_4066	hypothetical protein	Silent mutation
4406980	g	g	c	SVEN_4066	hypothetical protein	Silent mutation
4407586	g	g	c	SVEN_4066	hypothetical protein	Missense mutation, K1961N
4407589	a	a	t	SVEN_4066	hypothetical protein	Silent mutation
4407625	t	t	g	SVEN_4066	hypothetical protein	Silent mutation
4407677-4407678	ca	ca	at	SVEN_4066	hypothetical protein	Missense mutation, Q1992M
4407680	t	t	g	SVEN_4066	hypothetical protein	Missense mutation, S1993A
4407685	g	g	c	SVEN_4066	hypothetical protein	Silent mutation
4407718	g	g	a	SVEN_4066	hypothetical protein	Silent mutation
4407730	g	g	a	SVEN_4066	hypothetical protein	Silent mutation
4603957-4603958	--	--	cgaggtcgggccaccggccgccggtggcc	NA	NA	downstream of SVEN_4253
4929017	c	c	t	SVEN_4575	RNA polymerase ECF-subfamily sigma factor	Silent mutation
5940646	-	-	c	NA	NA	downstream of SVEN_5474
6278783	g	g	--	NA	NA	downstream of SVEN_5782
6871710	acc	acc	--	SVEN_6297	Branched-chain amino acid	deletion mutation resulting in

					transport system permease protein LivM	the loss of threonine (T) at residue 384.
7101604	c	c	t	SVEN_6499	hypothetical protein	Missense mutation, T1213M.
7593311	t	t	c	SVEN_6941	hypothetical protein	Silent mutation

The genome of *S. venezuelae* ATCC 10712 (StrepDB: https://strepdb.streptomyces.org.uk/) was used to map the position of sequence mutation in MU-1. NA, not applied; +, present of the sequence; --, absent of the sequence.
