# Supplementary material for: Impact of the alkaline volatile trimethylamine on the physiology of Streptomyces venezuelae: an integrated transcriptomic and metabolomics study
Source: Microbiol Spectr. 2025 May 27;13(7):e00318-25. doi: 10.1128/spectrum.00318-25 (PMC12211087; doi:10.1128/spectrum.00318-25)
Supplement: Reviewer comments [file reviewer-comments.pdf]

Microbiology Spectrum

Impact of the alkaline volatile trimethylamine on the physiology of Streptomyces venezuelae: an integrated transcriptomic and metabolomics study

Yanping Zhu, Hanlei Zhang, Meng Liu, and Xiuhua Pang

Corresponding Author(s): Xiuhua Pang, Shandong University - Qingdao Campus

Review Timeline:

Submission Date:	January 31, 2025
Editorial Decision:	February 26, 2025
Revision Received:	April 25, 2025
Editorial Decision:	April 28, 2025
Revision Received:	April 29, 2025
Accepted:	May 1, 2025

Editor: Jeffrey Gardner

Reviewer(s): Disclosure of reviewer identity is with reference to reviewer comments included in decision letter(s). The following individuals involved in review of your submission have agreed to reveal their identity: Ban Al-Joubori (Reviewer #1)

Transaction Report:

(Note: With the exception of the correction of typographical or spelling errors that could be a source of ambiguity, letters and reports are not edited. The original formatting of letters and referee reports may not be reflected in this compilation.)

DOI: https://doi.org/10.1128/spectrum.00318-25

Re: Spectrum00318-25 (Impact of the alkaline volatile trimethylamine on the physiology of Streptomyces venezuelae: an integrated transcriptomic and metabolomics study)

Dear Dr. Xiuhua Pang:

Thank you for the privilege of reviewing your work. Below you will find my comments, instructions from the Spectrum editorial office, and the reviewer comments.

Overall, the reviewers found the results interesting, but thought that additional experiments were required, specifically re-sequencing of the the MU-1 mutant strain to identify the mutations present from the parent strain. You can find additional specific requested changes from the individual reviewers. Therefore, the manuscript is being returned to you for modification and re-submission.

Please return the manuscript within 60 days; if you cannot complete the modification within this time period, please contact me. If you do not wish to modify the manuscript and prefer to submit it to another journal, notify me immediately so that the manuscript may be formally withdrawn from consideration by Spectrum.

Revision Guidelines
To submit your modified manuscript, log into the submission site at https://spectrum.msubmit.net/cgi-bin/main.plex. Go to Author Tasks and click the appropriate manuscript title to begin. The information you entered when you first submitted the paper will be displayed; update this as necessary. Note the following requirements:

• Upload point-by-point responses to the issues raised by the reviewers in a file named "Response to Reviewers," NOT in your cover letter.
• Upload a compare copy of the manuscript (without figures) as a "Marked-Up Manuscript" file.
• Upload a clean .DOC/.DOCX version of the revised manuscript and remove the previous version.
• Each figure must be uploaded as a separate, editable, high-resolution file (TIFF or EPS preferred), and any multipanel figures must be assembled into one file.
• Any supplemental material intended for posting by ASM should be uploaded with their legends separate from the main manuscript. You can combine all supplemental material into one file (preferred) or split it into a maximum of 10 files with all associated legends included.

For complete guidelines on revision requirements, see our Submission and Review Process webpage. Submission of a paper that does not conform to guidelines may delay acceptance of your manuscript.

Data availability: ASM policy requires that data be available to the public upon online posting of the article, so please verify all links to sequence records, if present, and make sure that each number retrieves the full record of the data. If a new accession number is not linked or a link is broken, provide Spectrum production staff with the correct URL for the record. If the accession numbers for new data are not publicly accessible before the expected online posting of the article, publication may be delayed; please contact production staff (Spectrum@asmusa.org) immediately with the expected release date.

Publication Fees: For information on publication fees and which article types are subject to charges, visit our website. If your manuscript is accepted for publication and any fees apply, you will be contacted separately about payment during the production process; please follow the instructions in that e-mail. Arrangements for payment must be made before your article is published.

ASM Membership: Corresponding authors may join or renew ASM membership to obtain discounts on publication fees. Need to upgrade your membership level? Please contact Customer Service at Service@asmusa.org.

The ASM Journals program strives for constant improvement in our submission and publication process. Please tell us how we can improve your experience by taking this quick Author Survey.

Thank you for submitting your paper to Spectrum.

Sincerely,
Jeffrey Gardner
Editor
Microbiology Spectrum

Reviewer #1 (Comments for the Author):

The article provides an in-depth investigation combining transcriptomics and metabolomics, making the findings robust. The

structure of the paper is organized effectively, with a well-structured abstract, introduction, methods, results, and discussion. Data from RNA-seq, metabolomics, and real-time PCR validate the findings.
Comment # 1: Some sentences are too complex or long. Shortening them would improve readability.
Example: Lines 106-108: "In contrast, the growth medium of strain MU-1 became acidic, reaching pH 4.7 at 72 h and maintaining a level close to pH 5 even after prolonged incubation, under the same growth conditions."
Comment # 2: Redundancies, some phrases repeat unnecessarily, making the text longer than needed.
Example: The expression level of each gene at 48 h in one set of ISP5230 samples was arbitrarily set to one."
Comment # 3: Some results lack direct interpretation.
Example:"Our data indicated that genes potentially involved in glucose transport were expressed at lower levels in MU-1 than in WT, suggesting reduced transport or uptake of glucose and consistent with the high level of residual glucose content in the growth medium of MU-1." Briefly explain why reduced glucose transport is significant for bacterial physiology.
Comment #4: The authors presents changes in gene expression and metabolite levels but lacks statistical validation (p-values, fold-change cutoffs, FDR correction).
Comment # 5: More direct comparisons, Ex. In discussing gene expression changes, stating how these changes affect physiology would help.
Comment # 6: In Methods Section: How were differential expression thresholds determined?
Comment # 7: Discussion could be more concise. The discussion repeats some results rather than focusing on interpretation. Also, the paragraph on glucose diffusion (lines ~370-377) could be shortened.
Comment #8: Some differentially expressed genes (DEGs) are reported without a functional interpretation. Authors should connect DEG changes to specific metabolic pathways or regulatory mechanisms.For Ex. If a TCA cycle gene is upregulated, discuss how this affects energy metabolism; If a stress response gene is induced, explain the potential impact on bacterial adaptation.
Overall Suggestions:
1. Improve clarity by simplifying complex sentences.
2. Avoid redundant phrases and unnecessary details.
3. Make direct comparisons in the results section.
4. Tighten the discussion to focus on interpretation rather than repeating data.

Reviewer #2 (Comments for the Author):

In this manuscript, the authors present their transcriptional analyses (RNA-seq and RT-PCR analyses) of wild type S. venezuelae, and a mutant strain termed MU-1. The MU-1 strain seems to have a similar expression profile to the wild type up until the wild type strain begins to differentiate, at which point the two strains diverge, with the wild type going on to sporulate and the mutant secreting copious amounts of organic acid and failing to progress to the stage of raising aerial hyphae; correspondingly, the two strains have very different gene expression profiles during these later stages of growth. There are a number of intriguing observations made, and the manuscript would benefit from the authors working to tie these observations into the wider literature, and connect the different systems within S. venezuelae (e.g. could the changes in primary metabolic genes impact the accumulation of precursors needed for the synthesis of the two antibiotics investigated?)

In order to make sense of the phenotype of MU-1 and the effects of TMA on this strain, it is important to know what chromosomal defects are present relative to the wild type. It is tantalizing to speculate that the MU-1 strain has a mutation in a gene needed for TMA synthesis; as the TMA biosynthetic pathway has yet to be identified in S. venezuelae, this would be a major finding/advance. It is possible to (re)sequence bacterial genomes for minimal costs these days, and consequently this is a critical experiment to do.

Experimental comments:

1) For all bar graphs with error bars, please add in the associated data points.

2) For the data presented in Figure 1B, the legend indicates that triplet samples were analyzed - it would be helpful to clarify whether these are independent/biological triplicates, or technical triplicates, with the former being the preferred option. It would also be helpful to include a schematic of the TCA cycle, and indicate which intermediates appear to be accumulating in strain MU-1. This schematic could also be very useful as a complement to the data presented in Figure 4, to help understand which genes are differentially impacted.

3) Lines 148-151: The manuscript text indicates that there were no differences in expression of developmental genes at 48h in the RNA-seq data, but that the chp genes were significantly altered in expression in the wild type and mutant strains at 96 hours. But in the next paragraph, and in Figure 2, the RT-PCR experiments show there are significant differences at 48 h. Can the authors please explain this discrepancy?

4) Fig S3: It would be helpful if a schematic of the biosynthetic cluster for both chloramphenicol and jadomycin could accompany the RT-PCR data presented, with the genes being assessed being indicated. This would help the reader to understand whether the increase in transcription is cluster-wide, or if it is largely confined to specific operons within the clusters.

5) The transcriptional upregulation of two antibiotic biosynthetic gene clusters is interesting. It would be worth doing a more comprehensive analysis to determine whether other specialized metabolic biosynthetic gene clusters are also upregulated.

6) The effect of TMA on the phenotype of strain MU-1, and on its gene expression, is interesting. What effect does it have on wild type? Does it further enhance/accelerate development, and impact gene expression?

Editorial comments:

1) Line 81: There is strong evidence that geosmin can function in promoting Streptomyces spore dispersal through the recruitment of soil-dwelling arthropods (Becher et al., 2020, Nature Microbiology).

2) There needs to be more information provided relating to RNA extraction and purification (lines 456-460).

3) Figure S1: the lack of aerial hyphae/spore formation for the wild type vs MU-1 strains would likely be better presented if the strains were streaked for single colonies, as opposed to plating for a lawn. In general, the growth of the two strains does not look to be terribly robust. It may be worth growing both on MYM agar, where the wild type grows and sporulates robustly. It is worth noting as well that S. venezuelae is typically considered to make green spores, not grey spores, as is currently indicated in the legend.

4) The pattern of expression of bldN would nicely explain the chp and rdl (and bldM) expression pattern, given that they are all direct targets for the BldN sigma factor. And the lack of expression of most of the whi genes is not surprising, given that most whi genes (and most chp and rdl) genes are upregulated in the aerial hyphae - if a strain does not raise aerial hyphae, the expression of these genes is likely to remain low. One exception to this is whiG, which has typically not been seen to have developmentally regulated expression, with its control instead coming more at the level of post-translational regulation. Some commentary on this could be appropriate within the discussion.

There is also a rich history of organic acids impacting development in other model Streptomyces species (Susstrunk et al., 1998; two papers in J. Bacteriol by Viollier et al. in 2001) that would be important to discuss.

Impact of the alkaline volatile trimethylamine on the physiology of *Streptomyces venezuelae*: an integrated transcriptomic and metabolomics study

Yanping Zhu[a, b*], Hanlei Zhang[a], Meng Liu[a, c], and Xiuhua Pang[a*]

[a]The State Key Laboratory of Microbial Technology, Shandong University, Qingdao 266237, China.
[b]Suzhou Research Institute, Shandong University, Suzhou 215123, China
[c] School of Municipal and Environmental Engineering, Shandong Jianzhu University, Jinan 250101, China

[*]For correspondence, Email: pangxiuhua@sdu.edu.cn or zhuyanping1123@sdu.edu.cn

Running title: TMA impacts *Streptomyces* physiology

Key words: *Streptomyces*, alkaline volatiles, TMA, organic acid, carbon metabolism

ABSTRACT

Streptomyces bacteria use volatile compounds for a range of functions, including communication and to kill competing species. We previously showed that the wild-type *Streptomyces venezuelae* strain ISP5230 produces the alkaline volatile trimethylamine (TMA), which can completely rescue the morphological defects of the *S. venezuelae* mutant strain MU-1, suggesting that TMA can have a significant impact on *Streptomyces* physiology. In this study, we further characterized strain MU-1 and elucidated the effects of alkaline volatiles on *S. venezuelae* by comparing the transcriptomic data of the wild-type strain ISP5230 and strain MU-1. Our RNA-Seq analysis revealed that MU-1 exhibited differentially expressed genes (DEGs) in multiple categories, including genes involved in development, carbon metabolism, antibiotic production, and transport, and these data were verified by real-time PCR analysis. Using metabolomics analysis, we next showed that organic acids accumulated in MU-1, consistent with the acidification of growth medium by MU-1. In addition, our quantitative analysis showed much higher residual glucose content in the growth medium of MU-1, suggesting inefficient consumption of glucose by this mutant. Notably, TMA exposure restored the normal expression pattern of the DEGs in MU-1, consistent with the rescue of the MU-1 phenotype, including morphology, acidification of growth medium, glucose uptake, and antibiotic production, by TMA exposure and indicating a global impact of TMA on *S. venezuelae* physiology. In conclusion, our study suggests that the alkaline volatile TMA profoundly affects the physiology of *S. venezuelae* and may promote the growth of *S. venezuelae* mutants, enabling their survival in complex microbial communities.

IMPORTANCE

Microbes produce a wide array of volatile compounds for which diverse biological roles have been implicated for microbial volatiles. In *Streptomyces*, the alkaline volatile trimethylamine (TMA) enables communication between cells, and in *S. venezuelae*, TMA could also rescue the defective morphology of the mutant strain MU-1, indicating a broader impact of TMA on *Streptomyces* physiology. In this study, we identified differentially expressed genes (DEGs) in MU-1 and demonstrated that TMA exposure induced normal expression of these DEGs in the mutant strain to levels comparable with those in the wild-type strain, consistent with the ability of TMA to rescue the abnormal phenotype of this mutant. Our study expands the role of TMA to a global impact on the physiology of recipient cells and adds new understanding of the importance of volatile

compounds in microbial communities.

INTRODUCTION

Streptomyces are soil-dwelling Gram-positive filamentous bacteria, best known as producers of bioactive compounds, including anti-infection agents and antibiotics, and for their ability to undergo cellular differentiation at late stages of development (1, 2). *Streptomyces* species have a relative complex life cycle compared with most prokaryotic bacteria (2, 3). The life cycle of a *Streptomyces* cell starts with the germination of a spore; the germinated spore grows into the substrate to acquire nutrients, forming so-called substrate hyphae or vegetative hyphae; then the vegetative hyphae break the surface of the solid substrate and grow upwards, forming aerial hyphae, which further develop into spore-bearing chains, and eventually mature spores are released, initiating another cycle under suitable conditions (2). Therefore, *Streptomyces* species normally have three types of cells, including spores, vegetative hyphae, and aerial hyphae, at different growth stages. However, *S. venezuelae* is able to develop a newly discovered type of cell, designated as an “explorer cell”, at the “exploration stage”, which is initiated upon interaction with yeast (4). *Streptomyces* cells in the exploration stage can produce the volatile compound trimethylamine (TMA), and the *Streptomyces* explorer cells can communicate their exploratory behavior to other physically separated *Streptomyces* cells at other stages of growth using TMA (4). In addition to TMA, *Streptomyces* can produce a wide range of volatile compounds (5), including geosmin, which makes the well-known earthy odor (6), although its biological role for the producer bacterium is not yet clear (7). *Streptomyces* can also produce high amounts of volatile ammonium to kill a wide range of competing bacteria, including Gram-positive and Gram-negative species, over a long distance (8). However, production of volatiles by microorganisms tends to be very conditional, and the process can be influenced by a multitude of factors, such as temperature, pH, and medium components (9). Production of TMA by *S. venezuelae* requires a specific growth condition, i.e., depletion of glucose in the medium (4, 10).

We previously found that the alkaline volatile TMA produced by *S. venezuelae* could rescue the defective morphology of *S. venezuelae* strain MU-1, which is a mutant strain with a bald phenotype (11), suggesting a potentially broad impact of TMA on the physiology of *S. venezuelae*. In this study, we revealed that MU-1 acidifies its growth medium and have reduced glucose consumption and we evaluated the impact of TMA by transcriptomic and metabolomics analysis, and we found

that TMA exposure could restore a normal expression pattern to genes that were differentially expressed in MU-1, likely explaining the rescue of the MU-1 phenotype by TMA exposure. Our study provides more insights into the biological role and mechanisms of action of TMA and into understanding the role of volatile compounds in the *Streptomyces* life cycle.

RESULTS

S. venezuelae mutant strain MU-1 acidifies the growth medium

We previously showed that strain MU-1 is defective in the formation of aerial mycelium and spores, resulting in the bald phenotype shown in Fig. S1, and that this defective phenotype can be rescued by alkaline volatiles produced by the wild-type *S. venezuelae* strain ISP5230 (WT) (11). We also previously found that, due to the production of alkaline volatiles, including TMA, the growth medium of the WT was basic (11); additional analysis supported that observation, with the WT growth medium reaching a pH value of 8.5 at 96 h and maintaining a constant pH value afterwards, (Fig. 1A). In contrast, the growth medium of strain MU-1 became acidic, reaching pH 4.7 at 72 h and maintaining a level close to pH 5 even after prolonged incubation, under the same growth conditions (Fig. 1A). The overall difference in pH value was over 3.5 pH points at 72 h and time afterwards between WT and MU-1, suggesting the accumulation of acidic products, potentially organic acids, during growth of MU-1.

Metabolomics analysis reveals accumulation of organic acids in MU-1

To test whether organic acids accumulated in MU-1, we performed metabolomics analysis and screened for metabolites with levels that were significantly changed between WT and MU-1 samples. Our metabolomics analysis showed that the levels of several metabolites from the tricarboxylic acid cycle (TCA) and glycolysis pathway were dramatically increased in MU-1, compared with WT (Fig. 1B). With TCA metabolites, the levels of citric acid, alpha-ketoglutaric acid, and fumaric acid in MU-1 were, respectively, about 6.4-fold ($\log_2$), 7.5-fold ($\log_2$), and 4.0-fold ($\log_2$) of that in WT. However, the metabolite with the most dramatic change was pyruvic acid of the glycolysis pathway, with a level in MU-1 of about 8.1-fold ($\log_2$) of that in WT (Fig. 1B). Collectively, our data revealed a remarkably high level of multiple organic acids in MU-1 samples, potentially explaining the acidification of the growth medium by MU-1.

RNA-Seq analysis reveals a global change in gene expression in MU-1

To investigate the molecular mechanisms underlying the dramatic differences in morphology and in the accumulation of high levels of organic acids in MU-1, as well as to investigate other potential gene expression differences that might not be reflected by the phenotypic changes, we performed RNA-Seq analysis on MU-1 using the WT strain ISP5230 as a control. Total RNA samples from ISP5230 and MU-1 were extracted from mycelia grown on N-Evans-CA medium, at two time points, 48 h and 96 h (Fig. S1). Our transcriptomic data indicated that, although thousands of genes were differentially expressed in MU-1, the fold change (log_2) for most genes was lower than 2. Those differentially expressed genes (DEGs) with fold changes lower than 2 were not further analyzed. We also excluded those DEGs with high fold changes but for which only minimal (<1) to low (<10) numbers of FPKM (fragments per kilobase per million) were detected in both strains. After these exclusions, at 48 h, 23 genes were upregulated, and 189 genes were downregulated; and at 96 h, 615 genes were upregulated and 1498 genes were downregulated in MU-1 compared with the parental strain ISP5230. Collectively, our RNA-Seq data indicated that more genes were downregulated than upregulated in MU-1. In general, the DEGs fell into multiple categories, including genes involved in development, carbon metabolism, antibiotic biosynthesis, and many more genes with unknown function, indicating a global change in gene expression in MU-1.

Differential expression of developmental genes in MU-1

To decipher the mechanisms underlying the bald phenotype of strain MU-1, we first analyzed the RNA-Seq data associated with developmental genes, including *ram* genes required for the synthesis of a surfactant protein that promotes growth of aerial hyphae (12); the *chp* and *rdl* genes, which encode chaplet and rodlin proteins that coat the outer sheath of spores and aerial mycelium (13); the developmental regulatory *bld* genes that control the switch from vegetative hyphae to aerial hyphae; and *whi* genes that control the formation of mature spores (3). No notable difference was detected in expression of any of these developmentally-associated genes (*chp*, *rdl*, *ram*, *bld*, and *whi*) at 48 h in MU-1 (Table S1); however, all *chp* and *rdl* genes were profoundly downregulated in MU-1 at 96 h (Table S1), with changes ranging from -3.47 to -7.59 (log_2), suggesting that much less chaplin and rodlet was produced in MU-1. In contrast, the *ram* genes, except *ramS*, appeared to be upregulated in MU-1, especially the levels for *ramR*, which were markedly higher at 96 h, reaching 3.73 (log_2).

We next analyzed the RNA-Seq data for the *bld* and *whi* developmental regulatory genes at 96

h (Table S1). Only *bldM* (-2.77, $\log_2$) and *bldN* (-4.71, $\log_2$) were markedly downregulated in MU-1. Most *whi* genes, including *whiD*, *whiH*, *whiD*, *whiI*, and all *whiE* genes, were dramatically downregulated in MU-1 at 96 h, while *whiB* and *whiG* were only slightly downregulated in MU-1 at this time (Table S1). The generally lower expression of *chp*, *rdl*, *bld*, and *whi* genes in MU-1 is consistent with the dramatic morphological changes of this strain.

Expression of development-associated genes was also verified by real-time PCR using RNA extracted at three time points (48, 72, and 96 h) from both the WT strain ISP5230 and strain MU-1. The expression level of each gene at 48 h in one set of ISP5230 samples was arbitrarily set to one. Our transcriptional data showed that the four tested *chp* genes (*chpC*, *chpE*, *chpG*, and *chpH*) were developmentally regulated in the WT strain ISP5230, demonstrating a temporal increase in expression and reaching the highest expression level at 96 h, ranging from a 14.8±0.54 fold change for *chpE* to a 54.3±6.7 fold change for *chpH*; however, this increase in expression was mostly abrogated in MU-1 (Fig. 2). A similar temporal expression pattern was observed for the three *rdl* genes, with a moderate expression level at 72 h and a peak level at 96 h, in the WT strain ISP5230, whereas expression of these *rdl* genes was only minimal in MU-1 (Fig. 2), in agreement with the RNA-Seq analysis. The expression profiles of the developmental regulatory genes *bldM* and *bldN*, and *whiB*, *whiD*, *whiE*, *whiG*, *whiH*, and *whiI* were similar to those of the *chp* and *rdl* genes in both the WT strain ISP5230 and strain MU-1, although with some variation in the expression levels (Fig. 3), in agreement with the RNA-Seq analysis. Collectively, the dramatically reduced expression of development-associated genes in MU-1, as determined by real-time PCR, is in agreement with the RNA-Seq analysis, and is consistent with the bald phenotype of MU-1.

Differential expression of carbon metabolism genes in MU-1

To investigate the mechanisms underlying the accumulation of organic acids by strain MU-1, we analyzed the RNA-Seq data associated with carbon metabolism genes, including genes that are involved in the tricarboxylic acid (TCA) cycle and glycolysis pathway, which produce multiple organic acid intermediates. Our RNA-Seq data indicated that the expression of most genes involved in the TCA cycle was not changed in MU-1 at 48 h, with only a few genes downregulated slightly at this time (Table S2); however, at 96 h, several TCA genes, including two *citA* genes, *fumC*, *sucB*, and the *sven_6835-6837* operon encoding succinate dehydrogenase, were expressed at markedly higher levels in MU-1 than in WT (Table S2). For genes involved in the glycolysis pathway, only

sven_7329, which encodes a fructose-bisphosphate aldolase, had an expression level above the cutoff value at 48 h, while expression of the rest of the pathway genes was either not detected or below the cutoff value at this time (Table S3). At 96 h, genes in the operon *sven_3052-54*, responsible for the uptake of fructose, and *sven_7344*, encoding a putative glyceraldehyde-3-phophate dehydrogenase, were profoundly upregulated in MU-1, while *sven_0459*, encoding another putative glyceraldehyde-3-phophate dehydrogenase, was markedly downregulated in MU-1, and other genes were upregulated only slightly in MU-1 (Table S3).

Expression of carbon metabolism genes was also investigated by real-time PCR. Our transcriptional analysis demonstrated that the expression level of all tested genes in the TCA cycle were comparable between MU-1 and the WT strain at 48 h (Fig. 4), which is consistent with the RNA-Seq analysis; however, the expression of these genes was generally higher in MU-1 than in WT strain at 72 h and 96 h (Fig. 4), consistent with the accumulation of TCA cycle-associated organic acids in MU-1. According to their expression profiles, genes of the glycolysis pathway were divided into three groups (Fig. S2): group 1 includes *gap2* and *eno*, which had expression levels much higher in WT than in MU-1 at 96 h; group 2 genes (*sven_1571*, *sven_1575*, and *sven_5075*) had expression levels much higher in MU-1 than in WT at 96 h; and group 3 genes (*sven_7329* and *sven_7344*) had markedly higher expression in MU-1 only at 48 h, while the differences at the two later time points were negligible (Fig. S2).

Differential expression of pyruvate metabolism genes in MU-1

We noticed that pyruvate was the most highly accumulated organic acid in MU-1 (Fig. 1C). Additionally, the expression level of *sven_5075*, encoding an enzyme (pyruvate kinase) involved in the biosynthesis of pyruvate, was elevated in MU-1 at 96 h in our RNA-Seq and real-time PCR analyses, potentially explaining the high level of pyruvate in MU-1. To further investigate the mechanism underlying pyruvate accumulation, we analyzed genes encoding enzymes that further process pyruvate. Our RNA-Seq data indicated that expression of *aceE1*, *bkdA1*/*B1*, and *poxB*, each encoding a pyruvate dehydrogenase responsible for converting pyruvate into acetyl-CoA, and *sven_2684* and *sven_4658*, encoding enzymes for the turnover of pyruvate into other molecules, were markedly downregulated in MU-1 (Table S4). Real-time PCR analysis showed that these genes were expressed only minimally in MU-1, contrasting with high expression levels in WT (Fig. 5), suggesting much less turnover of pyruvate in MU-1. Collectively, our data indicated higher

expression of pyruvate biosynthetic genes and markedly lower expression of pyruvate metabolic genes in MU-1 than in WT, explaining the dramatic level of pyruvate accumulation in MU-1.

Differential expression of antibiotic biosynthetic genes in MU-1

S. venezuelae produces two signature antibiotics, chloramphenicol (CHL) and jadomycin (JAD). To investigate the potential changes in antibiotic production in MU-1, we first analyzed the RNA-Seq data associated with the biosynthesis of these two antibiotics. Expression of most *cml* genes was markedly upregulated in MU-1 at 96 h, but not at 48 h (Table S5). Additionally, transcription of four representative genes from the *cml* gene cluster was compared in the WT strain and MU-1 by real-time PCR analysis (Fig. S3), and the results showed that expression of the four *cml* genes *cmlR*, *cmlI*, *cmlM*, and *cmlN* was generally higher in MU-1 than in the WT strain at each time point (Fig. S3A), except for *cmlI* at 48 h. We therefore investigated whether production of CHL is changed in MU-1. Our quantification analysis showed that the level of CHL for MU-1 was about twice the level found with the WT strain at 72 h and 96 h (Fig. S3B), in agreement with the higher expression of *cml* genes in MU-1. The RNA-Seq data also indicated that expression of about half of the *jad* genes, involved in JAD biosynthesis, were upregulated in MU-1 at 96 h (Table S6); upregulation of these genes at late time points, represented by *jadR** and *jadY*, was verified by real-time PCR analysis (Fig. S3C). Collectively, our data indicated that several antibiotic biosynthetic genes were more highly expressed in MU-1.

Reduced glucose consumption by MU-1

Our metabolomics analysis revealed not only accumulation of organic acids but also a high level of glucose in the growth medium of MU-1 (Fig. 6A), suggesting less consumption of glucose by MU-1. To investigate the dynamic consumption of glucose, we measured the residual glucose content in the growth medium of WT and MU-1 over seven days (Fig. 6B). The glucose content decreased steadily from the initiation of growth (100% level) to 26.1±0.3% at 96 h in WT, indicating an initial rapid consumption of glucose by WT, which was followed by a tapering off in consumption, with the glucose content remaining fairly steady after 96 h, at about 26.7±1.8% at 120 h, 24.6±0.8% at 144 h, and 26.1±0.4% at 168 h, suggesting exhaustion of the glucose accessible to bacteria, given that diffusion of glucose is limited in the agar. In contrast, the decrease in glucose content occurred very slowly with MU-1 (Fig. 6B), and the glucose content for MU-1 was at least

40% higher than for WT at 48 h, and times afterwards, indicating that at least 40% less glucose was consumed by MU-1; this higher remaining glucose content in the growth medium of MU-1 was in agreement with the metabolomics analysis.

To investigate the mechanisms underlying the reduced glucose consumption with MU-1, we analyzed the RNA-Seq data associated with sugar transporter genes (Table S7). Our RNA-Seq data indicated that, while some unrelated transporter genes were profoundly upregulated in MU-1, three genes potentially involved in sugar transport (*sven_0974*, *sven_265*8, and *sven_2659*) were downregulated in MU1-1, especially *sven_2658* (*nagE1*), reaching a change of -6.97(log_2), suggesting that much less sugar is transported into cells. Expression of these genes was also investigated by real-time PCR (Fig. 6C). The expression level of *sven_0974* was always higher in WT-1 than in MU-1 at 48 h (0.98±0.01 vs 0.33±0.03), 72 h (1.05±0.02 vs 0.21±0.01), and 96 h (0.98±0.01 vs 0.33±0.03). The expression level of *nagE1* was comparable at 48 h; however, expression was higher in WT at 72 h (2.94±0.21 vs 0.76±0.02), and dramatically higher at 96 h (23.5±5.8 vs 0.44±0.02), when compared to MU-1. Similarly, the expression level of *nagE2* was much higher in WT than in MU-1 at 48 h and 96 h. According to the transcriptional profiles, *sven_0974* was constitutively expressed at a fairly constant level in WT, implying it might encode a transporter for a critical energy supplier, for example, glucose, whereas *nagE1* and *nagE2* were developmentally regulated, with greater expression at later time points, suggesting a role in the transport of molecules that are needed at the late growth phase. Collectively, our data indicated that genes potentially involved in glucose transport were expressed at lower levels in MU-1 than in WT, suggesting reduced transport or uptake of glucose and consistent with the high level of residual glucose content in the growth medium of MU-1.

TMA exposure complements expression of developmental genes in MU-1

TMA exposure can rescue the morphological defect of MU-1, restoring the formation of aerial mycelium and spores, as demonstrated in this study (Fig. S4), and as reported previously (11). To investigate the underlying mechanism, expression of development-associated genes was investigated by real-time PCR using RNA extracted at three time points (48, 96, and 120 h) from the mutant strain MU-1 exposed to water (MU-1 H_2O) and TMA (MU-1 TMA), using the WT strain ISP5230 exposed to water (WT H_2O) as a control. The expression level of each gene at 48 h in one set of WT H_2O samples was arbitrarily set to one (Fig. 7). The expression level of *chpC* was close

to a value of one for all three sample sets at 48 h, when the strains were still in vegetative growth. At 96 h, when the WT H_2O control had already formed aerial mycelium (Fig. S4), the expression of *chpC* increased to a fold change of 12.1±1.2, whereas expression remained at a basal level (1.5±0.4) in MU-1 H_2O; however, *chpC* expression reached a level of 14.4±1.1 in MU-1 TMA, and at 120 h, while *chpC* expression was still minimal in MU-1 H_2O, the levels were comparable between WT H_2O and MU-1 TMA. Similarly, the expression levels of *rdlA*, *bldN*, *whiD*, *whiE*, *whiH*, and *whiI* were restored in MU-1 TMA to levels comparable to those in WT H_2O at the two late time points (Fig. 7) when MU-1 TMA forms aerial mycelium and spores (Fig. S4). Collectively, our data showed that TMA exposure induced expression of developmental genes in MU-1 to levels comparable to those of the WT strain, consistent with the rescue of the morphological phenotype of MU-1 by TMA exposure.

TMA exposure complements expression of carbon metabolism genes in MU-1

TMA exposure not only rescued the morphological defect of MU-1, but also resulted in a rise in the pH of the growth medium of MU-1 from acidic to basic, with pH levels at 72 h and later comparable with those for the wild-type strain ISP5230 (Fig. S5), suggesting that conversion of organic acids to other products was normal in MU-1 exposed to TMA. To investigate the mechanism underlying the rise in pH values, we examined expression of genes involved in the TCA cycle and glycolysis pathway, since as noted, these processes produce organic acid intermediates, and several of these genes were upregulated in MU-1 (Fig. 4 and Fig. S2). For this analysis, we performed real-time PCR using RNA extracted at three time points (48, 96, and 120 h) with MU-1 exposed to TMA or water, as above. At 48 h, *citA* was upregulated in MU-1 H_2O, with an expression level of about twice that in WT H_2O (1.86±0.07 vs 0.85±0.11), whereas the expression level decreased to 0.71±0.02 in MU-1 exposed to TMA, comparable to that in WT (Fig. 8). At 96 h, by when vegetative growth has ceased and reproductive growth has already initiated, the expression level of *citA* had decreased to 0.12±0.01 in WT H_2O, but remained much higher in MU-1 H_2O (0.48±0.07); in contrast, expression decreased to 0.08±0.01 in MU-1 TMA, close to the value in WT H_2O (Fig. 8). Additionally, the generally higher expression levels of *fumC*, *sacA*, *sdhC*, *pdhL*, and *korB* in MU-1 H_2O were restored in MU-1 TMA to levels comparable to those in WT H_2O (Fig. 8A, Fig. S6A), indicating expression of genes involved in the TCA cycle was restored in MU-1 after TMA exposure. Similarly, expression of genes involved in the glycolysis pathway (Fig. S6B)

and in the metabolism of pyruvate (Fig. 8B, Fig. S6C) was restored in MU-1 TMA to levels comparable to those in WT H_2O. Overall, these data indicated that TMA exposure of MU-1 restored the normal (i.e., WT) expression pattern of genes that impact the levels of organic acids, explaining the pH increase in MU-1 after TMA exposure.

TMA exposure restores antibiotic production by MU-1

We also measured the levels of CHL produced by MU-1 exposed to TMA. Although the CHL level was higher in MU-1 H_2O than in the WT H_2O strain at 96 and 120 h, the levels were comparable between MU-1 TMA and WT H_2O at these two times (Fig. S7A), indicating that TMA exposure restored normal production levels of CHL to MU-1. Our transcriptional analysis showed that the higher levels of *cmlI*, *cmlM*, and *cmlN* expression detected in MU-1 H_2O at each time point were reduced in MU-1 TMA to levels comparable to those in WT H_2O (Fig. S7B), consistent with the reduced production of CHL. In addition, normal expression of *jadR** and *jadY* was restored in MU-1 TMA (Fig. S7C). These data indicated that TMA exposure reduced the expression of *cml* and *jad* genes to a WT level.

TMA exposure promotes glucose consumption by MU-1

We revealed high residual glucose content in the growth medium of MU-1 (Fig. 6A, B). Given the complementation of the morphological defects, acidic pH, and abnormally high CHL production in MU-1 by TMA exposure, we were curious if normal glucose consumption would also be restored. Notably, the temporal glucose content curve of MU-1 TMA nearly overlapped that of WT H_2O, which had at least a 30% lower glucose content than MU-1 H_2O did at 48 h and times afterwards (Fig. 6D), indicating that TMA exposure promoted glucose consumption by MU-1. Our transcriptional analysis showed that expression of the sugar transport genes *sven_0974* and *nagE1* was restored to a level comparable with that of the wild-type strain (Fig. S8), consistent with an increased uptake and transport of glucose by MU-1 exposed to TMA and explaining the lower glucose content in the growth medium of MU-1 TMA.

We previously showed that the alkaline volatile ammonium also raises the pH of the growth medium of MU-1, as TMA did; however, the morphology of MU-1 was not rescued by ammonium treatment (11). To investigate whether ammonium exposure could restore normal glucose consumption to the mutant, we measured the glucose content in an ammonium-treated sample.

However, the glucose content in the growth medium of MU-1 exposed to ammonium was comparable to that of the control MU-1 sample (Fig. S9), indicating that, unlike TMA, ammonium exposure does not promote glucose consumption.

DISCUSSION

Microbes produce a wide range of volatile compounds that are usually low-molecular-weight molecules and thus can spread easily in soil, air, and water (5, 14). Diverse biological roles, including antibacterial and antifungal activities, enhancing plant growth, raising the pH, altering antibiotic resistance profiles, protest attractant, and others, have been revealed for these microbial volatiles (5, 14-17); most often, the effect of volatiles appears to be uni-directional, manifested mainly by changes in organisms perceiving the volatiles rather than the producer itself (18). We previously demonstrated a strain-specific effect of the *Streptomyces* volatile TMA, which was able to rescue the morphological defects of an *S. venezuelae* mutant strain (MU-1), a newly discovered role for bacterial volatiles (11). In this study, by integrated transcriptomic and metabolomics analyses, we further revealed a global impact of TMA on the physiology of *S. venezuelae*, rather than the limited effect on one or two traits reported for other known volatiles (5). We showed that TMA exposure induces expression of developmental genes to levels comparable with those of the wild-type strain, explaining why TMA exposure was able to rescue the bald phenotype of MU-1, resulting in the normal formation of aerial mycelium and spores. We further revealed abundant accumulation of multiple organic acids, with pyruvate being the most highly accumulated, in the growth medium of strain MU-1, explaining our observation that the growth medium of MU-1 was acidified. We showed that genes for the biosynthesis of pyruvate were highly expressed in MU-1 whereas genes for conversion of pyruvate to other products were just minimally expressed, explaining the high accumulation of pyruvate in MU-1. In addition, the expression levels of most genes involved in the TCA cycle were elevated in MU-1, consistent with the accumulation of multiple organic acids associated with this pathway.

It was previously reported TMA was produced by *S. venezuelae* when the growth conditions included depletion of glucose and interaction with yeast (4, 10), which presented a seeming paradox, given our finding that TMA can be produced by *S. venezuelae* grown on solid agar with glucose (11). However, our current study suggests that the glucose accessible to the bacteria can be exhausted on agar medium, due to incomplete diffusion of glucose through the solid substrate,

therefore creating a localized glucose-depleted environment that would favor production of TMA by the wild-type strain by 96 h of growth under the conditions we used; by this time, the mutant strain MU-1 started forming aerial mycelium when co-incubated with the wild-type strain (11), consistent with the production of TMA by wild-type *S. venezuelae* and with the finding that TMA production needs a glucose-depleted environment (4). In addition, our data showed that a high level of glucose was always maintained in the growth medium of the mutant strain MU-1, even after prolonged incubation, suggesting that the residual glucose inhibited TMA production and potentially explaining why strain MU-1 could not produce TMA.

Ammonium is another alkaline volatile produced by *S. venezuelae* (8). Although co-incubation with ammonium or TMA could raise the pH of the MU-1 growth medium, the defective morphology of MU-1 was not restored by ammonium exposure (11), suggesting that the rescue of the MU-1 morphology, and potentially other defective traits of MU-1 as well, is TMA-specific. Although our study indicated that induction of a more normal gene expression pattern is responsible for the ability of TMA exposure to complement several of the abnormal traits of MU-1, the mechanism by which TMA, a low-molecular-weight molecule capable of penetrating the bacterial membrane, triggers a global positive effect is still not known. Although we did not examine gene expression patterns under ammonium exposure, the failure of ammonium to rescue the morphology and other traits of MU-1 suggests that ammonium exposure did not stimulate a normal pattern of gene expression, including for carbon metabolism genes, in MU-1, and therefore ammonium exposure could not promote glucose consumption by MU-1, in contrast to TMA; instead, a high level of glucose remained in the medium during ammonium exposure, and such an environment would have prevented the production of the TMA needed to compensate for the defects of MU-1. Although both TMA and ammonium are alkaline volatiles, and both were able to raise the pH of the growth medium, production of ammonium appears to be more beneficial to the producer by killing the surrounding bacteria (8), whereas production of TMA could be beneficial both to the producer, as demonstrated by inducing an exploratory state to obtain nutrients (8), as well as to the receiver as shown by the global positive impact on MU-1. Additionally, given that MU-1 produced higher than normal levels of CHL, TMA production could also enhance survival of the producer by rescuing mutants that are less fit but that might still contribute products that benefit the TMA producer. The importance of microbial volatiles for the ecology of microorganisms has been understudied for years. However, as basic environments normally favor bacterial growth while acidic environments

favor fungal growth, it is possible that *Streptomyces* species promote the richness of the bacterial community in the environment by emitting alkaline volatiles such as TMA.

MATERIALS AND METHODS

Culture conditions

Streptomyces venezuelae strain ISP5230 is used as the wild-type strain (19), MU-1 is a derivative strain of ISP5320 defective in growth (11). N-Evans-CA agar comprises Basic Evans medium (25 mM TES, 10 mM KCl, 2 mM $NaSO_4$, 2 mM citric acid, 0.25 mM $CaCl_2$, 1.25 mM $MgCl_2$, 1 mM $NaMoO_4$, and 0.5% Evans trace elements, pH 7.2) supplemented with glucose (2.5%) as carbon source, casamino acids (1%) as nitrogen source, and NaH_2PO_4 (2 mM) as phosphate source (20), and agar. N-Evans-CA was used for incubation of *S. venezuelae* strains, measurement of pH, determination of organic acids, RNA extraction, and metabolomics analysis.

Co-incubation analysis and measurement of pH

Equal numbers of *Streptomyces* spores were inoculated onto N-Evans-CA media and were incubated at 30°C at different times. For all experiments, solid agar was weighed first and cut into small pieces and then an equal weight of distilled water was added, followed by vortexing for 2 hours, centrifugation to remove the debris, and then pH levels were measured as described (11).

Metabolomics analysis

The frozen bacterial samples were placed in microcentrifuge tubes and resuspended with pre-chilled 80% methanol, thawed on ice, and whirled on an agitator for 30 s. After sonification for 6 min, the mixtures were centrifuged at 5,000 rpm, 4°C for 1 min. The supernatants were freeze-dried, resuspended with 10% methanol, and then the solutions were injected into an LC-MS/MS (liquid chromatography with tandem mass spectrometry) system.

UHPLC-MS/MS (ultra-high-performance liquid chromatography with tandem mass spectrometry) analyses were performed using a Vanquish UHPLC system (Thermo Fisher, Germany) coupled with an Orbitrap Q Exactive TM HF mass spectrometer or Orbitrap Q Exactive TMHF-X mass spectrometer (Thermo Fisher, Germany). Samples were injected onto a Hypersil Gold column (100×2.1 mm, 1.9μm) using a 12-min linear gradient at a flow rate of 0.2 mL/min. The eluents for the positive and negative polarity modes were eluent A (0.1% FA in Water) and eluent B (methanol).

The solvent gradient was set as follows: 2% B, 1.5 min; 2-85% B, 3 min; 85-100% B, 10 min; 100-2% B, 10.1 min; and 2% B, 12 min. The Q Exactive TM HF mass spectrometer was operated in positive/negative polarity mode with a spray voltage of 3.5 kV, capillary temperature of 320°C, sheath gas flow rate of 35 psi, and auxiliary (aux) gas flow rate of 10 L/min, S-lens RF level of 60, and aux gas heater temperature of 350°C.

The raw data files generated by UHPLC-MS/MS were processed using the Compound Discoverer 3.3 (CD3.3, Thermo Fisher) to perform peak alignment, peak picking, and quantitation for each metabolite. The main parameters were set as follows: peak area was corrected with the first quality control (QC) evaluation, actual mass tolerance, 5 ppm; signal intensity tolerance, 30%; and minimum intensity, and other parameters recommended by the manufacturer. After that, peak intensities were normalized to the total spectral intensity. The normalized data was used to predict the molecular formula based on additive ions, molecular ion peaks, and fragment ions, and then peaks were matched with the mzCloud (https://www.mzcloud.org/), mzVault, and MassList databases to obtain the accurate qualitative and relative quantitative results. Statistical analyses were performed using the statistical software R (R version R-3.4.3), Python (Python 2.7.6 version), and CentOS (CentOS release 6.6). When data were not normally distributed, they were standardized according to the formula: sample raw quantitation value/(the sum of sample metabolite quantitation value/the sum of QC1 sample metabolite quantitation value) to obtain relative peak areas; compounds whose cutoff values of relative peak areas in QC samples were greater than 30% were removed, and finally the metabolite identification and relative quantification results were obtained.

RNA isolation, reverse transcription-PCR (RT-PCR), and real-time PCR

For the RNA extractions, *Streptomyces* strains were grown at 30ºC on N-Evans-CA agar covered with plastic cellophane. Mycelia were collected at indicated times and then ground in liquid nitrogen before dispensing into REZol reagent (SBSBIO). To remove the chromosomal DNA, the crude RNA samples were treated twice with 'Turbo DNA-free' DNase reagents (Ambion), and then reverse transcription was conducted, as described (19). The SYBR Green *Pro Taq* HS DNA polymerase (AG Bio) was used with the manufacturer's recommended conditions to determine the melting curve of PCR products and their specificity, and for real-time PCR assays, using a Roche LightCycler480 thermal cycler. Expression of the *hrdB* gene, which encodes the *Streptomyces* major sigma factor, was used to normalize the relative quantities of cDNA.

RNA-sequence (RNA-Seq) and data analysis

RNA-Seq was performed by BGI Sequencing, as described (21, 22). The quality of total RNA was evaluated by NanoDrop 1000 (Thermo), and a cDNA library was constructed according to the manufacturer's instructions (Illumina). rRNA in total RNA was depleted by the Ribo-zero rRNA Removal solution (Illumina), cDNA was synthesized using random primers and SuperScript II (Invitrogen), and the ends of the cDNA fragments were paired and then sequenced by HiSeq2000 (Illumina). The expression level of each gene was standardized by the number of fragments per kilobase per million (FPKM). The cut-off value for assaying gene transcriptional activity was determined based on a 95% confidence interval for all FPKM values of each gene. The RNA-Seq data were deposited in NCBI (accession number PRJNA1196022).

Determination of glucose content

The glucose assay was performed using the O-toluidine assay kit (Beyotime Biotechnology). Briefly, *Streptomyces* strains were inoculated onto N-Evans-CA agar covered with cellophane for designated times. The growth agar was separated from the cellophane, homogenized, and then extracted with an equal volume of ddH_2O for 4-6 h. After centrifugation at 8000 rpm for 10 min, the supernatants were collected, and 5 μL samples were used to react with 185 μL O-toluidine reagent at 95°C for 10 min, and then the mixture were transferred to ice-chilled water for 10 min. The absorbance was measured at 630 nm. The standard curve of glucose was prepared using 0, 5, 10, 20, 50, 100, 200, 400, 800, 1200, 1600, and 2000 mg/L of glucose, and the glucose concentration of the samples was calculated according to the standard curves. The glucose concentration extracted from the blank (control) medium was set to 100%, and the glucose concentration of the media obtained at designated times was divided by the glucose concentration of the blank medium to determine the content (percentage) of remaining glucose in the medium.

Detection of antibiotic production

CHL was detected by HPLC analysis. Briefly, equal numbers of spores were inoculated into N-Evans-CA liquid medium and harvested at indicated times. Samples were prepared and tested essentially as described (23). Known concentrations of CHL (0.01 μg/mL, 0.1 μg/mL, 1.0 μg/mL, 10 μg/mL, 100 μg/mL, and 1000 μg/mL) were analyzed by HPLC, and the UV values were used to

generate a standard curve to calibrate the level of CHL in the samples.

Author contributions XP conceived of and supervised the study. YZ and HZ performed the experiments. ML and HZ prepared the figures. XP wrote the manuscript. All authors read and approved the manuscript.

Funding This work was supported by grants from the National Natural Science Foundation of China (NSF32270072 to XP and 32300046 to YZ), the Open Funding Project of State Key Laboratory of Microbial Metabolism (MMKF24-04 to XP), and the Natural Science Foundation of Jiangsu Province (BK20230247 to YZ).

Declaration of competing interest

The authors declare that they have no known competing financial interests or personal relationships that could have appeared to influence the work reported in this paper.

References

1. **Hopwood DA (ed.).** 2007. Streptomyces in Nature and Medicine. OXFORD UNIVERSITY PRESS.
2. **Chater K.** 2011. Differentiation in *Streptomyces*: the properties and programming of diverse cell-types, p. 43-86. *In* P D (ed.), *Streptomyces*: Molecular Biology and Biotechnology. Caister Academic Press.
3. **Elliot MA, Buttner, M.J., and Nodwell, J.R.** 2008. Multicellular development in *Streptomyces*, p. 419-438. *In* Whiteworth DE (ed.), Myxobacteria: Multicellularity and Differentiation American Society for Microbiology.
4. **Jones SE, Ho L, Rees CA, Hill JE, Nodwell JR, Elliot MA.** 2017. *Streptomyces* exploration is triggered by fungal interactions and volatile signals. Elife **6:**e21738.
5. **Netzker T, Shepherdson EMF, Zambri MP, Elliot MA.** 2020. Bacterial Volatile Compounds: Functions in Communication, Cooperation, and Competition. Annual Review of Microbiology, Vol 74, 2020 **74:**409-+.
6. **Gust B, Challis GL, Fowler K, Kieser T, Chater KF.** 2003. PCR-targeted *Streptomyces* gene replacement identifies a protein domain needed for biosynthesis of the sesquiterpene soil odor geosmin. Proc Natl Acad Sci U S A **100:**1541-1546.
7. **Garbeva P, Avalos M, Ulanova D, van Wezel GP, Dickschat JS.** 2023. Volatile sensation: The chemical ecology of the earthy odorant geosmin. Environmental Microbiology **25:**1565-1574.
8. **Avalos M, Garbeva P, Raaijmakers JM, van Wezel GP.** 2020. Production of ammonia as a low-cost and long-distance antibiotic strategy by *Streptomyces* species. Isme J **14:**569-583.
9. **Misztal PK, Lymperopoulou DS, Adams RI, Scott RA, Lindow SE, Bruns T, Taylor JW, Uehling J, Bonito G, Vilgalys R, Goldstein AH.** 2018. Emission Factors of Microbial Volatile Organic Compounds from Environmental Bacteria and Fungi. Environ Sci Technol **52:**8272-8282.
10. **Jones SE, Pham CA, Zambri MP, McKillip J, Carlson EE, Elliot MA.** 2019. Volatile Compounds Influence Exploration and Microbial Community Dynamics by Altering Iron Availability. Mbio **10:**e00171-00119.

11. **Zhu Y, Zeng Y, Liu M, Lu T, Pang X.** 2024. Rescue of morphological defects in *Streptomyes venezuelae* by the alkaline volatile compound trimethylamine. Microbiol Spectr **12**.

12. **Kodani S, Hudson ME, Durrant MC, Buttner MJ, Nodwell JR, Willey JM.** 2004. The SapB morphogen is a lantibiotic-like peptide derived from the product of the developmental gene ramS in *Streptomyces coelicolor*. Proc Natl Acad Sci U S A **101:**11448-11453.

13. **Claessen D, Stokroos I, Deelstra HJ, Penninga NA, Bormann C, Salas JA, Dijkhuizen L, Wosten HA.** 2004. The formation of the rodlet layer of streptomycetes is the result of the interplay between rodlins and chaplins. Mol Microbiol **53:**433-443.

14. **Weisskopf L, Schulz S, Garbeva P.** 2021. Microbial volatile organic compounds in intra-kingdom and inter-kingdom interactions. Nature Reviews Microbiology **19:**391-404.

15. **Audrain B, Farag MA, Ryu CM, Ghigo JM.** 2015. Role of bacterial volatile compounds in bacterial biology. Fems Microbiology Reviews **39:**222-233.

16. **Audrain B, Létoffé S, Ghigo JM.** 2015. Airborne Bacterial Interactions: Functions Out of Thin Air? Frontiers in microbiology **6:**1476.

17. **Schmidt R, Cordovez V, de Boer W, Raaijmakers J, Garbeva P.** 2015. Volatile affairs in microbial interactions. Isme J **9:**2329-2335.

18. **Camarena-Pozos DA, Flores-Núñez VM, López MG, Partida-Martínez LP.** 2021. Fungal volatiles emitted by members of the microbiome of desert plants are diverse and capable of promoting plant growth. Environmental Microbiology **23:**2215-2229.

19. **Zhang P, Wu L, Zhu Y, Liu M, Wang Y, Cao G, Chen XL, Tao M, Pang X.** 2017. Deletion of MtrA inhibits cellular development of *Streptomyces coelicolor* and alters expression of developmental regulatory genes. Frontiers in microbiology **8:**2013.

20. **Fink D, Weissschuh N, Reuther J, Wohlleben W, Engels A.** 2002. Two transcriptional regulators GlnR and GlnRII are involved in regulation of nitrogen metabolism in *Streptomyces coelicolor* A3(2). Mol Microbiol **46:**331-347.

21. **Liu M, Zhang P, Zhu Y, Lu T, Wang Y, Cao G, Shi M, Chen XL, Tao M, Pang X.** 2019. Novel two-component system MacRS is a pleiotropic regulator that controls multiple morphogenic membrane protein genes in *Streptomyces coelicolor*. Appl Environ Microbiol **85:**e02178-02118.

22. **Zhu Y, Wang X, Zhang J, Ni X, Zhang X, Tao M, Pang X.** 2022. The regulatory gene *wblA* is a target of the orphan response regulator OrrA in *Streptomyces coelicolor*. Environmental Microbiology **24:**3081-3096.

23. **Som NF, Heine D, Holmes NA, Munnoch JT, Chandra G, Seipke RF, Hoskisson PA, Wilkinson B, Hutchings MI.** 2017. The Conserved Actinobacterial Two-Component System MtrAB Coordinates Chloramphenicol Production with Sporulation in *Streptomyces venezuelae* NRRL B-65442. Frontiers in microbiology **8:**1145.

Figure captions

Fig. 1. Accumulation of organic acids in the *S. venezuelae* mutant strain MU-1. (A) Temporal pH curves of the *S. venezuelae* wild-type strain ISP5230 (WT) and strain MU-1 grown at 30°C on solid N-Evans-CA medium. The pH value is measured for the growth medium of each strain. (B) Accumulation of organic acids in the growth medium of MU-1. *S. venezuelae* WT and MU-1 strains were grown at 30°C on solid N-Evans-CA medium for 96 h, and organic acids were measured by UHPLC-MS/MS. Triplet samples were analyzed for each. The y-axis represents the value for the

indicated metabolites in the corresponding strain.

Fig. 2. Transcriptional analysis of *chp* and *rdl* genes in strain MU-1. ISP5230 (WT) and MU-1 were grown on solid N-Evans-CA medium, and RNA samples were isolated at the indicated times. Gene expression was measured by real-time PCR, and expression of *hrdB*, encoding the major sigma factor in *Streptomyces*, was used as an internal control. The y-axis shows the fold change in expression level of ISP5230 (black bars) and MU-1 (grey bars) over the level of each gene in ISP5230 at 48 h, at each time point, with the expression level of each gene in one set of ISP5230 samples at 48 h arbitrarily set to one. Results are the means (±SD) of triplet biological experiments.

Fig. 3. Transcriptional analysis of developmental regulatory genes in strain MU-1. ISP5230 (WT) and MU-1 RNA samples were prepared and analyzed by real-time PCR as described in the legend for Figure 2. The y-axis shows the fold change in expression level in ISP5230 (black bars) and MU-1 (grey bars) over the level of each gene in ISP5230 at 48 h, at each time point, with the expression level of each gene in one set of ISP5230 samples at 48 h arbitrarily set to one. Results are the means (±SD) of triplet biological experiments.

Fig. 4. Transcriptional analysis of genes in the TCA cycle in strain MU-1. ISP5230 (WT) and MU-1 RNA samples were prepared and analyzed by real-time PCR as described in the legend for Figure 2. The y-axis shows the fold change in expression level in ISP5230 (black bars) and MU-1 (grey bars) over the level of each gene in ISP5230 at 48 h, at each time point, with the expression level of each gene in one set of ISP5230 samples at 48 h arbitrarily set to one.. Results are the means (±SD) of triplet biological experiments.

Fig. 5. Transcriptional analysis of genes involved in pyruvate metabolism in strain MU-1. ISP5230 (WT) and MU-1 RNA samples were prepared and analyzed by real-time PCR as described in the legend for Figure 2. The y-axis shows the fold change in expression level in ISP5230 (black bars) and MU-1 (grey bars) over the level of each gene in ISP5230 at 48 h, at each time point, with the expression level of each gene in one set of ISP5230 samples at 48 h arbitrarily set to one. Results are the means (±SD) of triplet biological experiments.

Fig. 6. Evaluation of glucose content in the growth medium of MU-1 and expression of transporter genes. (A) Metabolomics analysis of glucose content in the growth medium of MU-1. *S. venezuelae* wild-type strain ISP5230 (WT) and MU-1 were grown at 30°C on solid N-Evans-CA medium for 96 h, and three samples were analyzed for each. The y-axis represents the value of glucose in the corresponding strain. (B) Temporal glucose content curves. ISP5230 (WT) and MU-1 were grown at 30°C on solid N-Evans-CA medium, and the residual glucose content of the medium was measured at the given time points. Results are the means (±SD) of triplet biological experiments. (C) Transcriptional analysis of transporter genes in strain MU-1. ISP5230 (WT) and MU-1 RNA samples were prepared and analyzed by real-time PCR as described in the legend for Figure 2. The y-axis shows the fold change in expression level in ISP5230 (black bars) and MU-1 (grey bars) over the level of each gene in ISP5230 at 48 h, at each time point, with the expression level of each gene in one set of ISP5230 samples at 48 h arbitrarily set to one. Results are the means (±SD) of triplet biological experiments. (D) Effects of TMA exposure on glucose content. Cultures of ISP5230 exposed to water (WT H_2O) and MU-1 exposed to water (MU-1 H_2O) or TMA (MU-1 TMA) were grown at 30°C on solid N-Evans-CA medium, and glucose content was measured as described for panel B. Results are the means (±SD) of triplet biological experiments.

Fig. 7. TMA exposure restores the expression of developmental genes in MU-1. Cultures of ISP5230 exposed to H_2O (WT H_2O) and MU-1 exposed to H_2O (MU-1 H_2O) or TMA (MU-1 TMA) were grown on solid N-Evans-CA medium, and RNA samples were prepared and analyzed by real-time PCR as described in the legend for Figure 2. The y-axis shows the fold change in expression level in WT H_2O (orange bars), MU-1 H_2O (blue bars), and MU-1 TMA (green bars) over the level of each gene in WT H_2O at 48 h, at each time point, with the expression level of each gene in one set of WT H_2O samples at 48 h arbitrarily set to one. Results are the means (±SD) of triplet biological experiments.

Fig. 8. TMA exposure restores expression of carbon metabolism genes in MU-1. Transcriptional analysis of genes involved in (A) the TCA cycle and (B) pyruvate metabolism. Cultures of ISP5230 exposed to H_2O (WT H_2O) and MU-1 exposed to H_2O (MU-1 H_2O) or TMA (MU-1 TMA) were grown on solid N-Evans-CA medium, and RNA samples were prepared and analyzed by real-time

PCR as described in the legend for Figure 2. The y-axis shows the fold change in expression level in WT H_2O (orange bars), MU-1 H_2O (blue bars), and MU-1 TMA (green bars) over the level of each gene in WT H_2O at 48 h, at each time point, with the expression level of each gene in one set of WT H_2O samples at 48 h arbitrarily set to one. Results are the means (±SD) of triplet biological experiments.

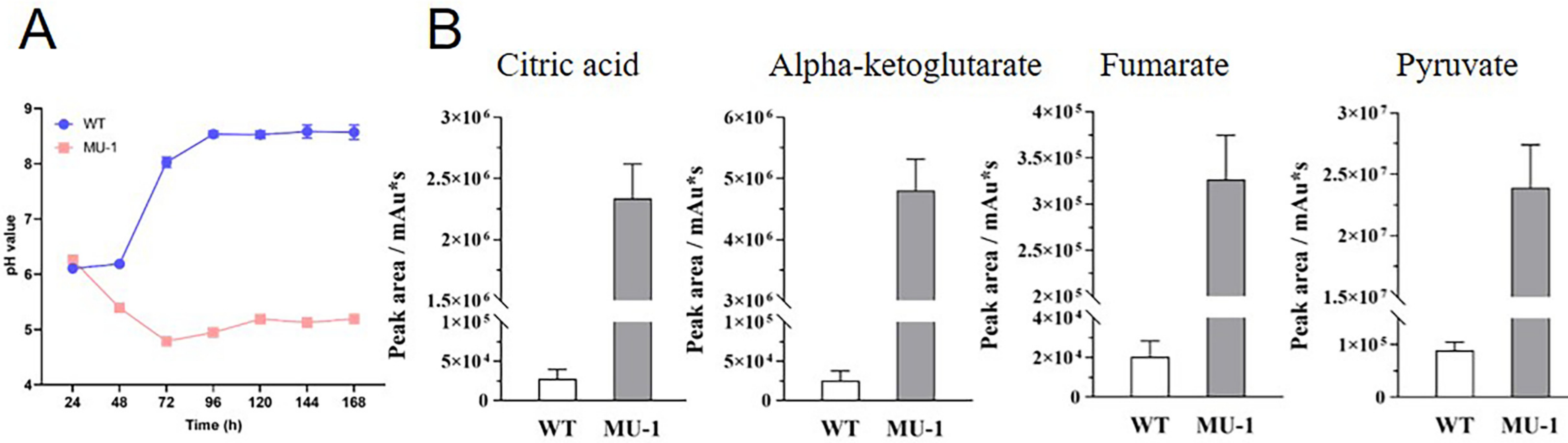
A
B
WT
MU-1
pH value
Time (h)
24
48
72
96
120
144
168
Citric acid
Alpha-ketoglutarate
Fumarate
Pyruvate
Peak area / mAu*s
WT
MU-1

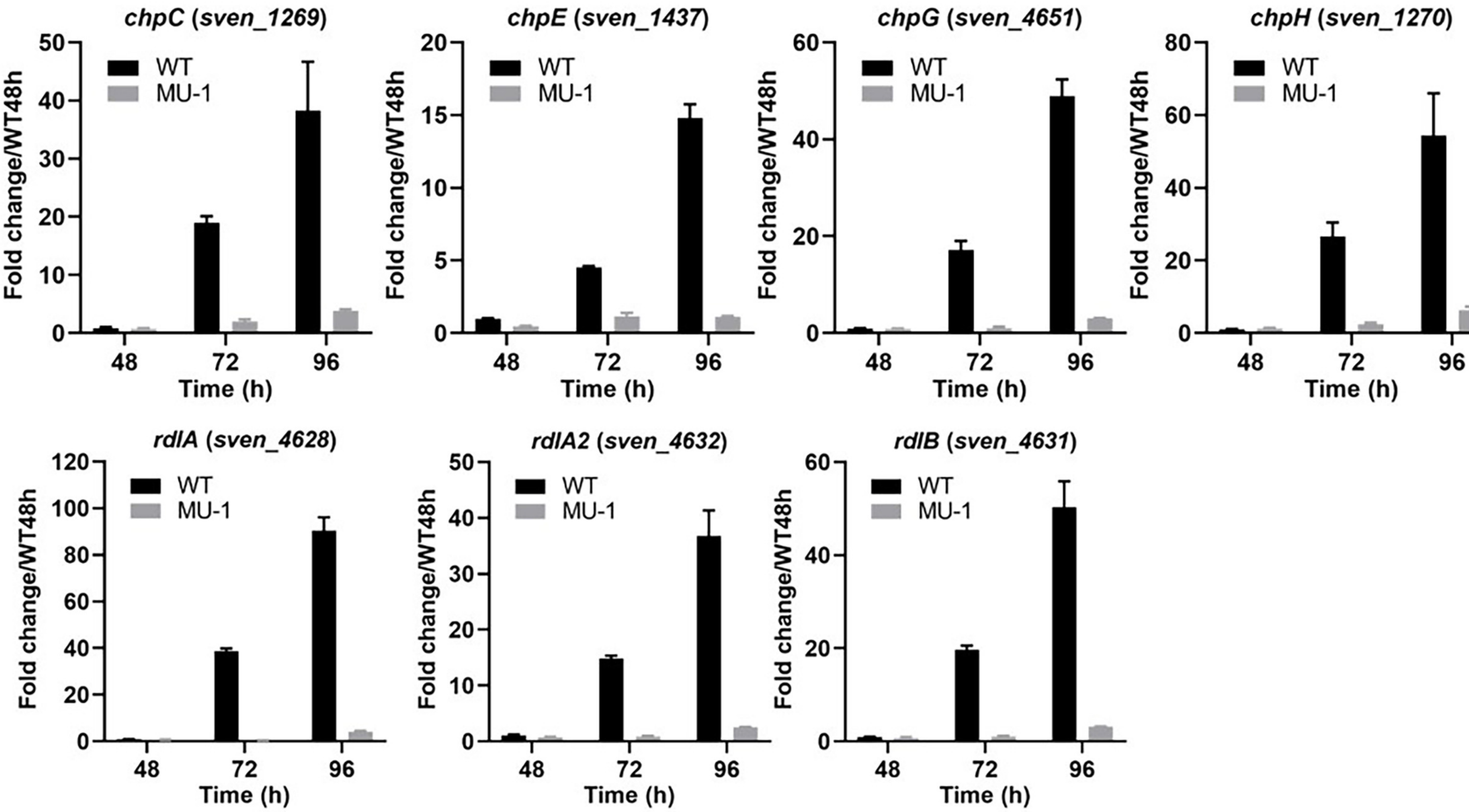

chpC (sven_1269)
chpE (sven_1437)
chpG (sven_4651)
chpH (sven_1270)
rdlA (sven_4628)
rdlA2 (sven_4632)
rdlB (sven_4631)
WT
MU-1
Fold change/WT48h
Time (h)
48
72
96

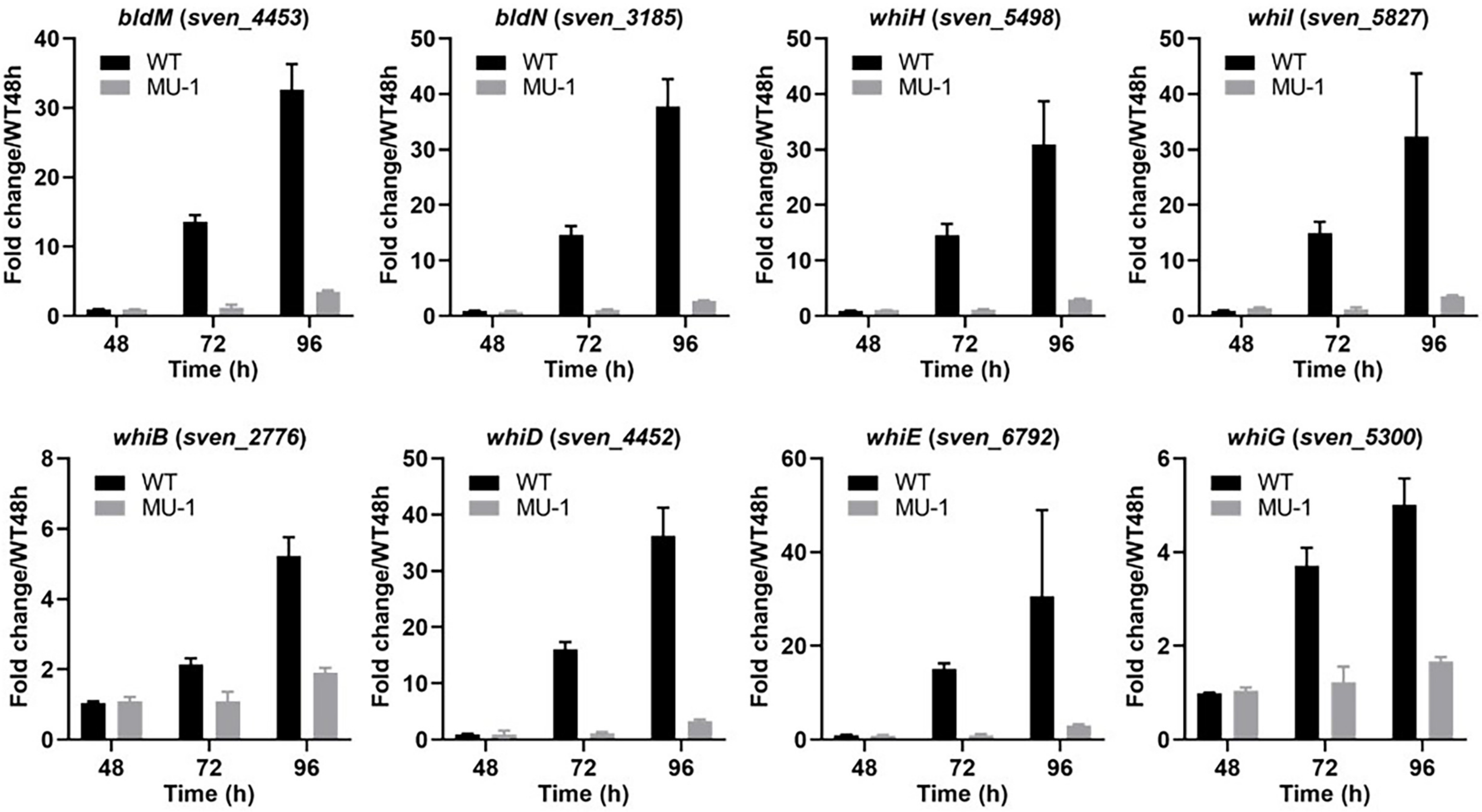

bldM (sven_4453)
bldN (sven_3185)
whiH (sven_5498)
whiI (sven_5827)
whiB (sven_2776)
whiD (sven_4452)
whiE (sven_6792)
whiG (sven_5300)
WT
MU-1
Fold change/WT48h
Time (h)
48
72
96

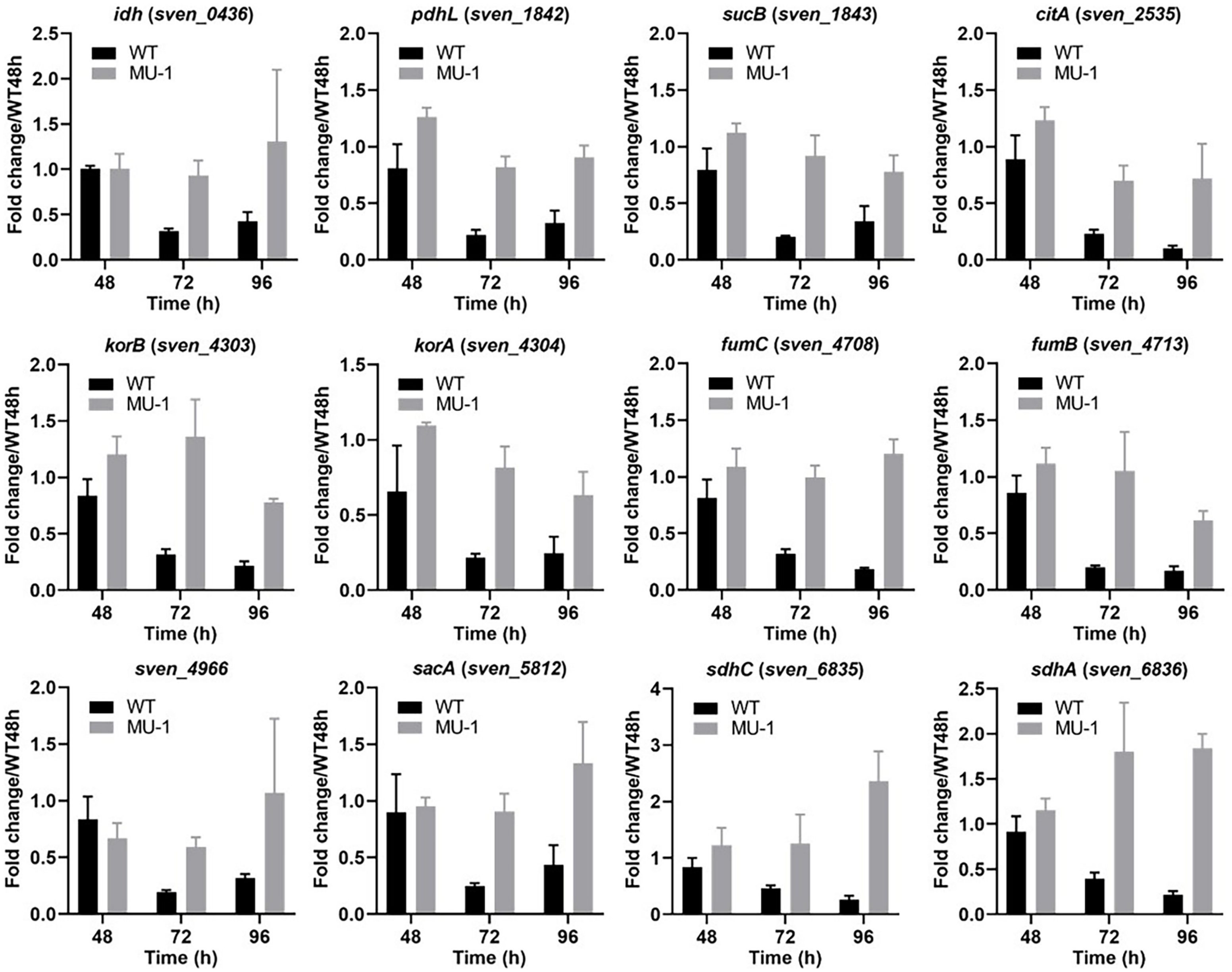
idh (sven_0436)
pdhL (sven_1842)
sucB (sven_1843)
citA (sven_2535)
korB (sven_4303)
korA (sven_4304)
fumC (sven_4708)
fumB (sven_4713)
sven_4966
sacA (sven_5812)
sdhC (sven_6835)
sdhA (sven_6836)
WT
MU-1
Fold change/WT48h
Time (h)
48
72
96

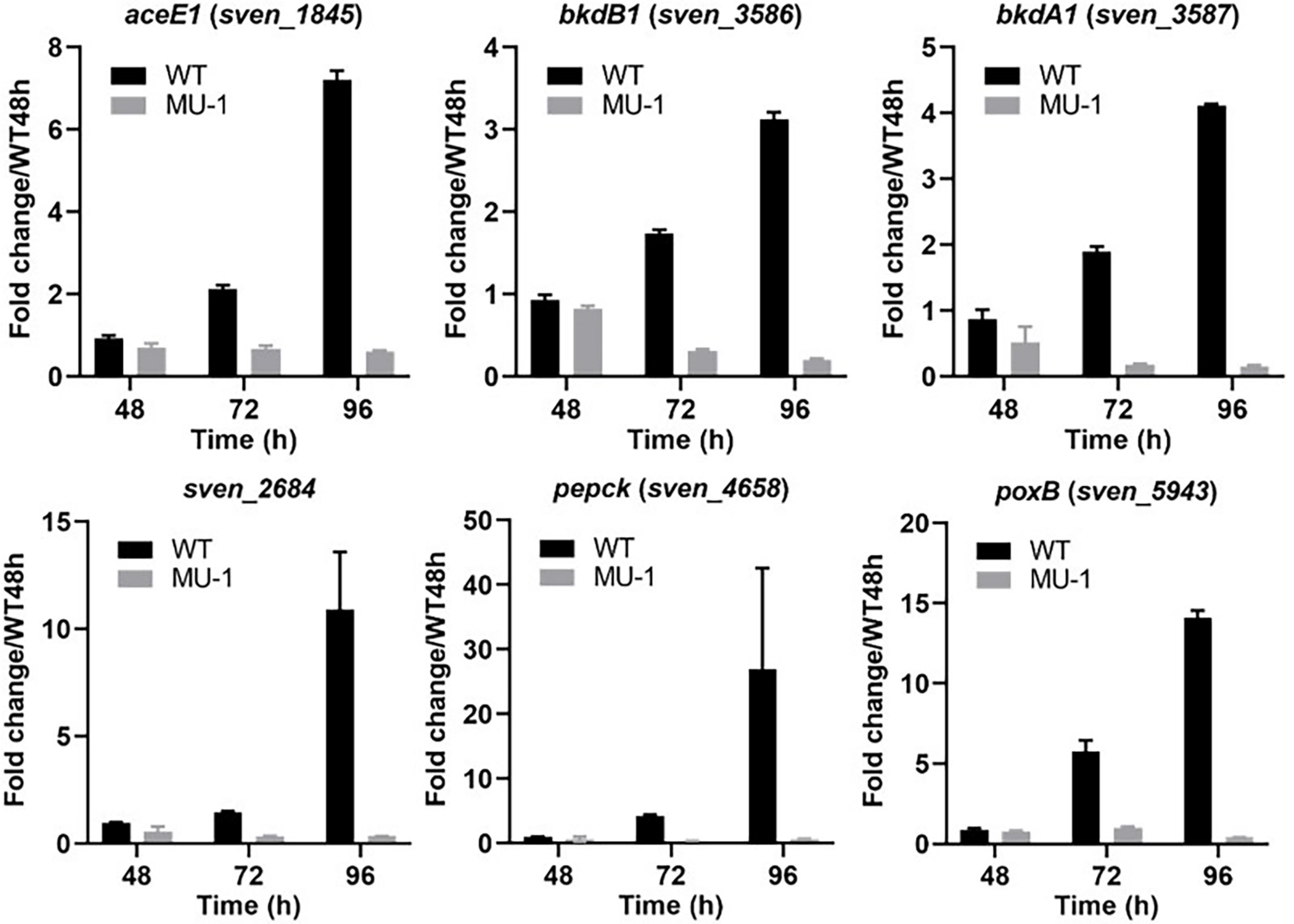

aceE1 (sven_1845)
bkdB1 (sven_3586)
bkdA1 (sven_3587)
sven_2684
pepck (sven_4658)
poxB (sven_5943)
WT
MU-1
Fold change/WT48h
Time (h)
48
72
96

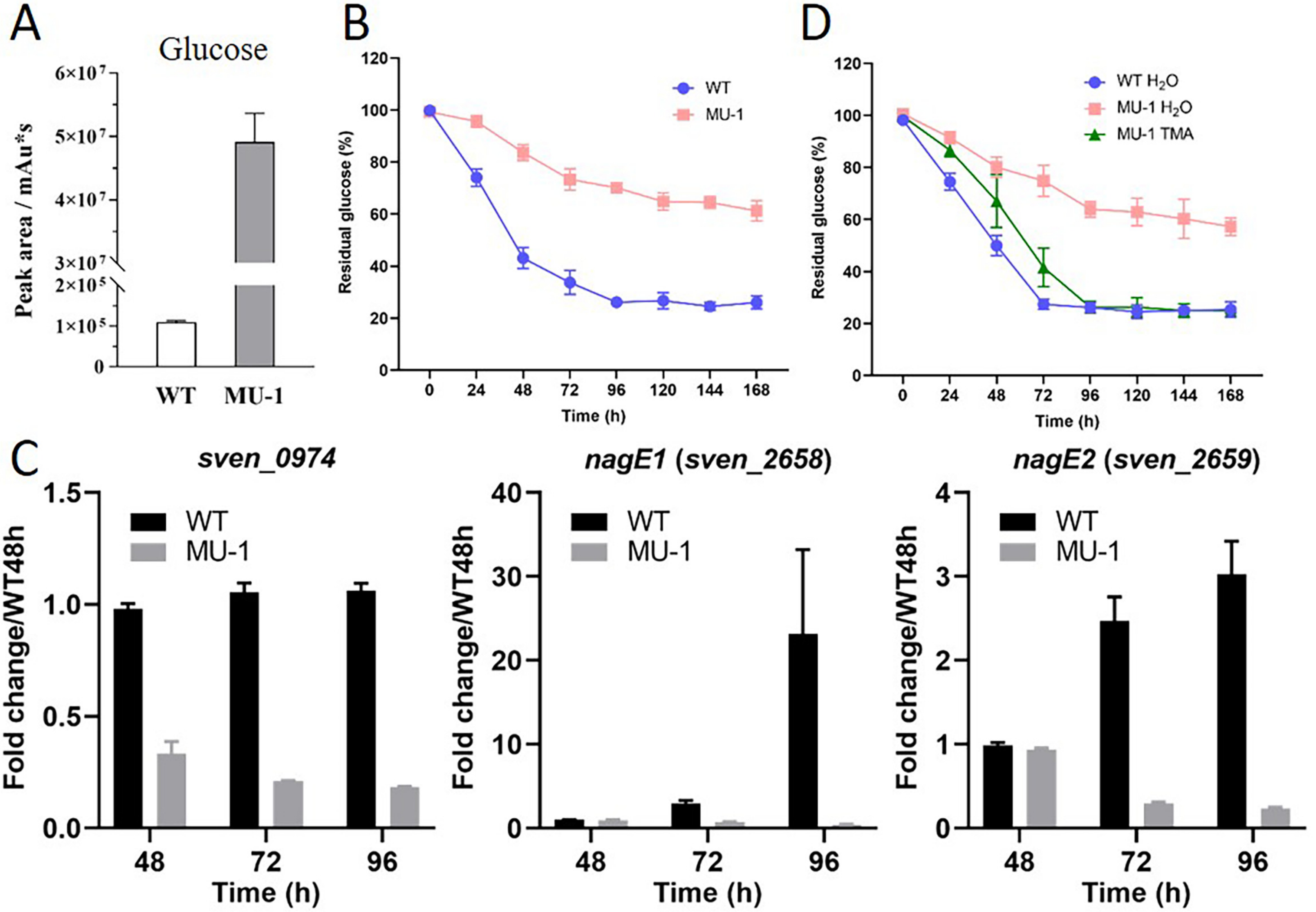

A
Glucose
Peak area / mAu*s
6×10^7
5×10^7
4×10^7
3×10^7
2×10^5
1×10^5
0
WT
MU-1
B
Residual glucose (%)
WT
MU-1
Time (h)
D
WT H2O
MU-1 H2O
MU-1 TMA
C
sven_0974
nagE1 (sven_2658)
nagE2 (sven_2659)
Fold change/WT48h
Time (h)

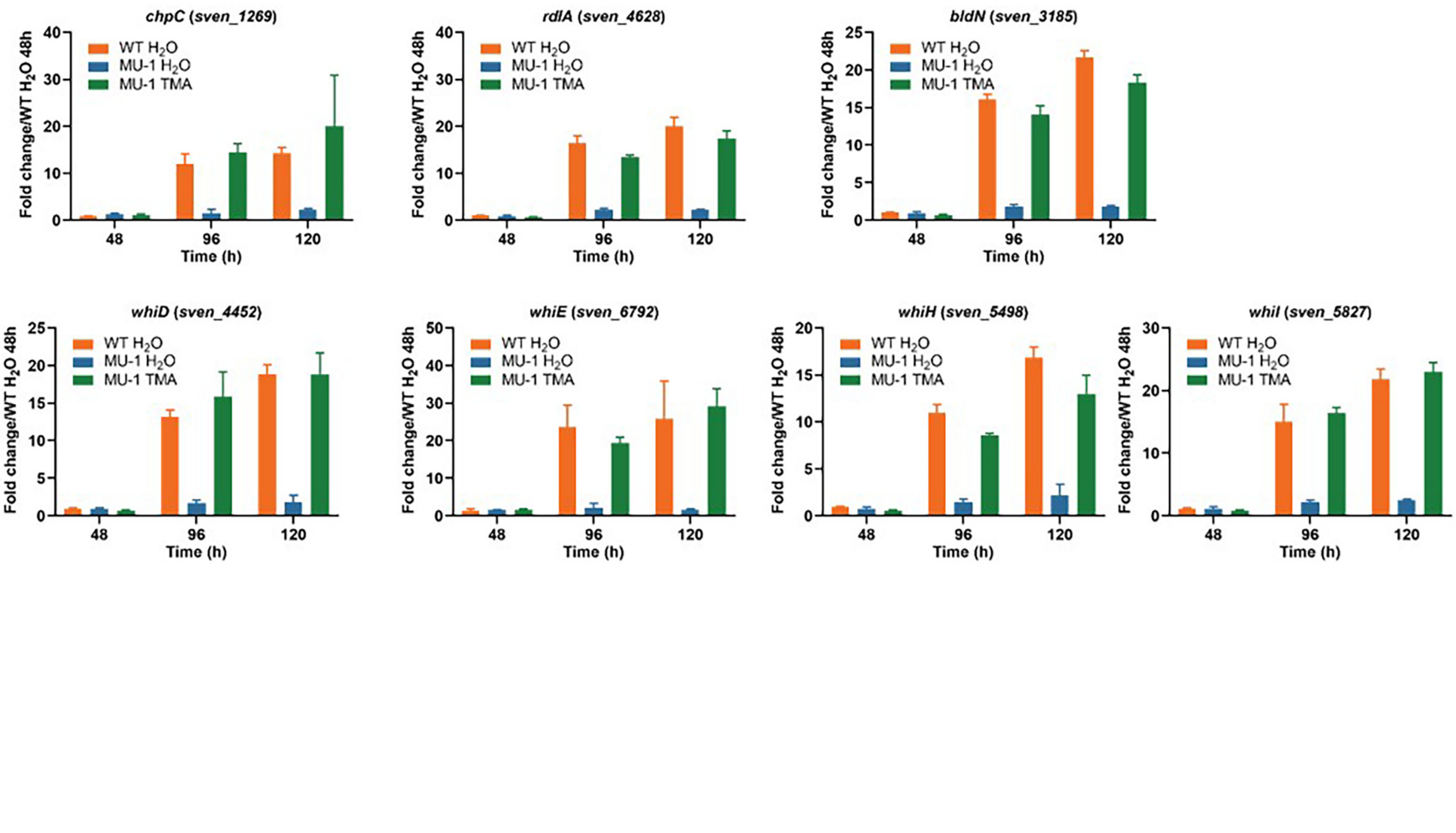

chpC (sven_1269)
rdlA (sven_4628)
bldN (sven_3185)
whiD (sven_4452)
whiE (sven_6792)
whiH (sven_5498)
whiI (sven_5827)
WT H_2O
MU-1 H_2O
MU-1 TMA
Fold change/WT H_2O 48h
Time (h)
48
96
120

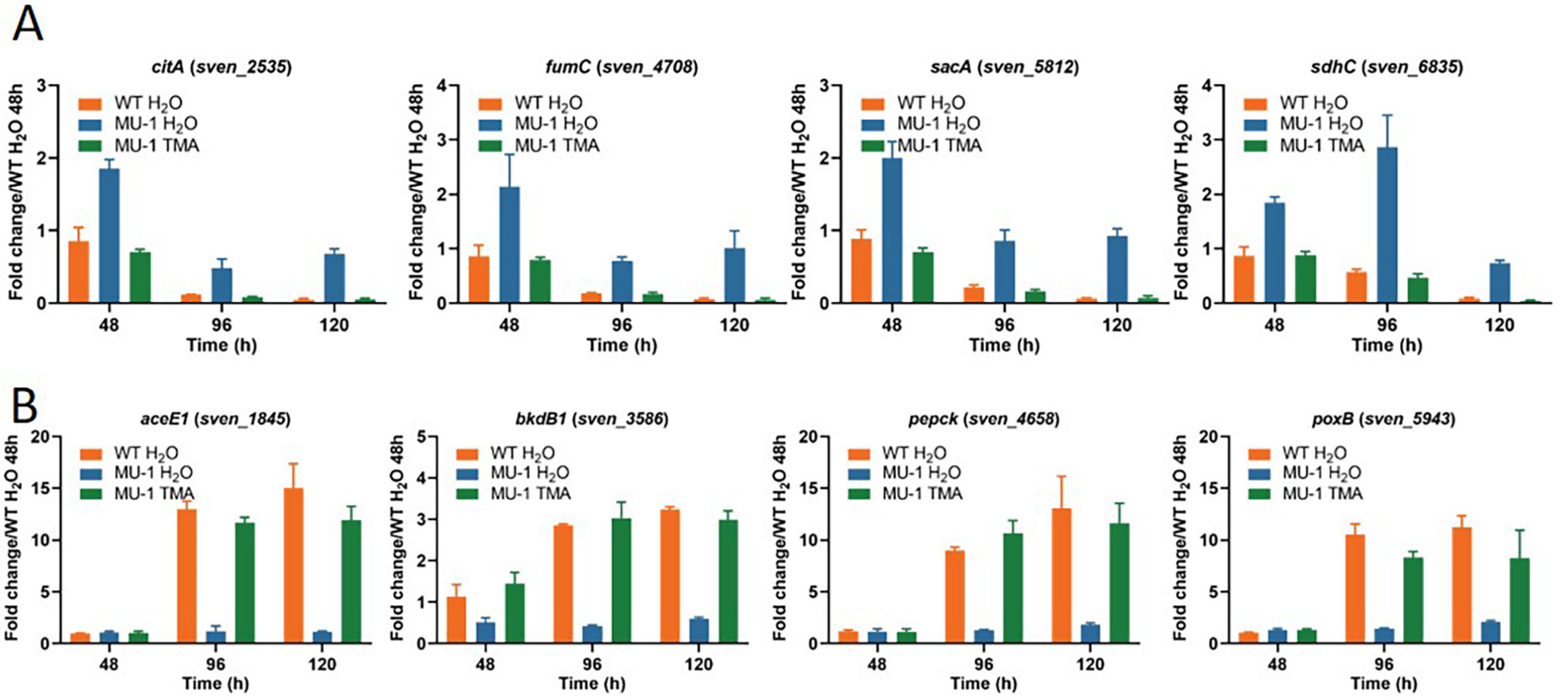
A
citA (sven_2535)
fumC (sven_4708)
sacA (sven_5812)
sdhC (sven_6835)
WT H2O
MU-1 H2O
MU-1 TMA
Fold change/WT H2O 48h
Time (h)
48
96
120
B
aceE1 (sven_1845)
bkdB1 (sven_3586)
pepck (sven_4658)
poxB (sven_5943)

Response to comments

We thank the reviewers for their helpful comments and have addressed their concerns below, including providing additional data and analyses.

Reviewer #1 (Comments for the Author):

Comment # 1: Some sentences are too complex or long. Shortening them would improve readability. Example: Lines 106-108: "In contrast, the growth medium of strain MU-1 became acidic, reaching pH 4.7 at 72 h and maintaining a level close to pH 5 even after prolonged incubation, under the same growth conditions."
Reply: Complex or long sentences were reduced, divided into two sentences, or reworded for clarity, as recommended (see, for example, lines 45-47, 92-93, 102, 107-109, 126-129, 174-176, 252-253, 304).

Comment # 2: Redundancies, some phrases repeat unnecessarily, making the text longer than needed. Example: The expression level of each gene at 48 h in one set of ISP5230 samples was arbitrarily set to one."
Reply: Repeated phrases were deleted from the main text, as recommended. However, some repetitive information was maintained in the figure captions to clarify how gene expression levels were determined in each experiment.

Comment # 3: Some results lack direct interpretation. Example: "Our data indicated that genes potentially involved in glucose transport were expressed at lower levels in MU-1 than in WT, suggesting reduced transport or uptake of glucose and consistent with the high level of residual glucose content in the growth medium of MU-1." Briefly explain why reduced glucose transport is significant for bacterial physiology.
Reply: A brief explanation was provided, as suggested (lines 278-280).

Comment #4: The authors presents changes in gene expression and metabolite levels but lacks statistical validation (p-values, fold-change cutoffs, FDR correction).
Reply: For most of our data, the differences in gene expression and metabolite levels are quite large, and we believe the trends are readily evident without statistical validation (p-values). However, since the levels of gene expression in Figure 4 and Figure 8A were low, we performed statistical analyses for these data, which showed that the results were mostly statistically significant, as described (lines 200-202, 313-314). Additionally, the information for the fold-change cutoffs for RNA-Seq was provided (lines 131-136, 540-543).

Comment # 5: More direct comparisons, Ex. In discussing gene expression changes, stating how these changes affect physiology would help.
Reply: The potential effects of gene expression changes on physiology were provided, as suggested (lines 389-395).

Comment # 6: In Methods Section: How were differential expression thresholds determined?
Reply: Information about how differential expression thresholds were determined was provided in lines 540-543.

Comment # 7: Discussion could be more concise. The discussion repeats some results rather than focusing on interpretation. Also, the paragraph on glucose diffusion (lines ~370-377) could be shortened.
Reply: We shortened several sentences and paragraphs in the discussion, including the paragraph on glucose diffusion (lines 390-394). We have also added more interpretation of our findings.

Comment #8: Some differentially expressed genes (DEGs) are reported without a functional interpretation. Authors should connect DEG changes to specific metabolic pathways or regulatory mechanisms. For Ex. If a TCA cycle gene is upregulated, discuss how this affects energy metabolism; If a stress response gene is induced, explain the potential impact on bacterial adaptation.
Reply: Most DEGs were connected to specific metabolic pathways or regulatory mechanisms (please refer to Tables S1-S9). However, the effect of the increased expression of TCA cycle genes in MU-1 was added, as suggested (lines 186-187).

Reviewer #2 (Comments for the Author):

Major comments: In order to make sense of the phenotype of MU-1 and the effects of TMA on this strain, it is important to know what chromosomal defects are present relative to the wild type. It is tantalizing to speculate that the MU-1 strain has a mutation in a gene needed for TMA synthesis; as the TMA biosynthetic pathway has yet to be identified in S. venezuelae, this would be a major finding/advance. It is possible to (re)sequence bacterial genomes for minimal costs these days, and consequently this is a critical experiment to do.
Reply: The genomes of the wild-type strain and MU-1 were sequenced, and a description of the sequence differences has been provided in the text (lines 357-374). The sequence differences are also listed in Table S10. As described in the results, multiple mutations were found in MU-1. Therefore, determining which mutation(s) is associated with TMA production is beyond the scope of fhis study, which focuses on the effect of TMA on physiology. However, such studies will be important in the future to elucidate the pathway associated with TMA synthesis.

Experimental comments:
1) For all bar graphs with error bars, please add in the associated data points.
Reply: We apologize, but we were not entirely clear whether the reviewer was asking for average numbers to be placed above each bar or something else. Although some papers in Microbiology Spectrum show average numbers in bar graphs, it is not a

standard format in the journal. Additionally, we believe the trends are readily visible without the numbers. However, we can add them if it would be considered helpful.

2) For the data presented in Figure 1B, the legend indicates that triplet samples were analyzed - it would be helpful to clarify whether these are independent/biological triplicates, or technical triplicates, with the former being the preferred option. It would also be helpful to include a schematic of the TCA cycle, and indicate which intermediates appear to be accumulating in strain MU-1. This schematic could also be very useful as a complement to the data presented in Figure 4, to help understand which genes are differentially impacted.
Reply: In Figure 1B, the analyzed samples were biological triplicates, and this information was added in the legend (line 664). A schematic of the TCA cycle was also added in Figure 1 (Fig. 1C), and accumulated intermediates were marked, as recommended.

3) Lines 148-151: The manuscript text indicates that there were no differences in expression of developmental genes at 48h in the RNA-seq data, but that the chp genes were significantly altered in expression in the wild type and mutant strains at 96 hours. But in the next paragraph, and in Figure 2, the RT-PCR experiments show there are significant differences at 48 h. Can the authors please explain this discrepancy?
Reply: Please note that the differences in expression of these developmental genes between WT and MU-1 were only minimal at 48 h in the real-time PCR results (Figure 2). The descriptions related to significant differences refer to the later time points (72 h and 96 h). Therefore, there is no notable difference between the RNA-Seq data and real-time PCR data at 48 h.

4) Fig S3: It would be helpful if a schematic of the biosynthetic cluster for both chloramphenicol and jadomycin could accompany the RT-PCR data presented, with the genes being assessed being indicated. This would help the reader to understand whether the increase in transcription is cluster-wide, or if it is largely confined to specific operons within the clusters.
Reply: Schematics of the biosynthetic clusters for chloramphenicol and jadomycin were added to Figs. S3, S4, S8, and S9, as recommended.

5) The transcriptional upregulation of two antibiotic biosynthetic gene clusters is interesting. It would be worth doing a more comprehensive analysis to determine whether other specialized metabolic biosynthetic gene clusters are also upregulated.
Reply: Further analysis of the RNA-Seq data revealed that three more gene clusters known to be involved or potentially involved in the biosynthesis of natural products were differentially expressed in MU-1. This new information was added in the text (lines 241-244) and in Tables S7 and S8.

6) The effect of TMA on the phenotype of strain MU-1, and on its gene expression, is interesting. What effect does it have on wild type? Does it further enhance/accelerate

development, and impact gene expression?

Reply: We have not explored the effect of excess TMA on the wild-type strain in comparison to normal levels. However, due to the very positive effect of TMA on the morphology of MU-1, we hypothesize that TMA could also enhance/accelerate development of the wild-type strain and have a positive impact on gene expression in this strain.

Editorial comments:

1) Line 81: There is strong evidence that geosmin can function in promoting Streptomyces spore dispersal through the recruitment of soil-dwelling arthropods (Becher et al., 2020, Nature Microbiology).

Reply: The function of geosmin was added, and the paper was cited, as recommended (lines 81-83).

2) There needs to be more information provided relating to RNA extraction and purification (lines 456-460).

Reply: More information was provided relating to RNA extraction and purification, as recommended (lines 514-524).

3) Figure S1: the lack of aerial hyphae/spore formation for the wild type vs MU-1 strains would likely be better presented if the strains were streaked for single colonies, as opposed to plating for a lawn. In general, the growth of the two strains does not look to be terribly robust. It may be worth growing both on MYM agar, where the wild type grows and sporulates robustly. It is worth noting as well that S. venezuelae is typically considered to make green spores, not grey spores, as is currently indicated in the legend.

Reply: It is very true that wild-type *S. venezuelae* produces green spores on agar medium such as MYM. However, it may produce grey spores on medium such as R2. The spores that it produced under the growth conditions used in this study were of a light greenish-grey color (Fig. S1A, revised). Normally, strain MU-1 only displays defects in morphology on the N-Evans-CA medium that we used in this study; N-Evans-CA is a relatively poor nutrient medium on which MU-1 forms very small colonies, and that is why we plated for lawn growth.

4) The pattern of expression of bldN would nicely explain the chp and rdl (and bldM) expression pattern, given that they are all direct targets for the BldN sigma factor. And the lack of expression of most of the whi genes is not surprising, given that most whi genes (and most chp and rdl) genes are upregulated in the aerial hyphae - if a strain does not raise aerial hyphae, the expression of these genes is likely to remain low. One exception to this is whiG, which has typically not been seen to have developmentally regulated expression, with its control instead coming more at the level of post-translational regulation. Some commentary on this could be appropriate within the discussion.

Reply: Related information was added in the discussion, as recommended (lines 417-428).

5) There is also a rich history of organic acids impacting development in other model Streptomyces species (Susstrunk et al., 1998; two papers in J. Bacteriol by Viollier et al. in 2001) that would be important to discuss.
Reply: The three previous studies were cited and discussed, as suggested (lines 400-416).

Re: Spectrum00318-25R1 (Impact of the alkaline volatile trimethylamine on the physiology of Streptomyces venezuelae: an integrated transcriptomic and metabolomics study)

Dear Dr. Xiuhua Pang:

Thank you for the privilege of reviewing your work. Below you will find my comments, instructions from the Spectrum editorial office, and the reviewer comments.

The authors have made a good effort to address all of the reviewers' comments in this revision. However, before the manuscript can be accepted all the sequencing data must be deposited in a public database. Therefore, the manuscript is being returned so that data can be deposited in a similar fashion to the RNAseq data and accession numbers included.

Please return the manuscript within 60 days; if you cannot complete the modification within this time period, please contact me. If you do not wish to modify the manuscript and prefer to submit it to another journal, notify me immediately so that the manuscript may be formally withdrawn from consideration by Spectrum.

Revision Guidelines
To submit your modified manuscript, log into the submission site at https://spectrum.msubmit.net/cgi-bin/main.plex. Go to Author Tasks and click the appropriate manuscript title to begin. The information you entered when you first submitted the paper will be displayed; update this as necessary. Note the following requirements:

• Upload point-by-point responses to the issues raised by the reviewers in a file named "Response to Reviewers," NOT in your cover letter.
• Upload a compare copy of the manuscript (without figures) as a "Marked-Up Manuscript" file.
• Upload a clean .DOC/.DOCX version of the revised manuscript and remove the previous version.
• Each figure must be uploaded as a separate, editable, high-resolution file (TIFF or EPS preferred), and any multipanel figures must be assembled into one file.
• Any supplemental material intended for posting by ASM should be uploaded with their legends separate from the main manuscript. You can combine all supplemental material into one file (preferred) or split it into a maximum of 10 files with all associated legends included.

For complete guidelines on revision requirements, see our Submission and Review Process webpage. Submission of a paper that does not conform to guidelines may delay acceptance of your manuscript.

Data availability: ASM policy requires that data be available to the public upon online posting of the article, so please verify all links to sequence records, if present, and make sure that each number retrieves the full record of the data. If a new accession number is not linked or a link is broken, provide Spectrum production staff with the correct URL for the record. If the accession numbers for new data are not publicly accessible before the expected online posting of the article, publication may be delayed; please contact production staff (Spectrum@asmusa.org) immediately with the expected release date.

Publication Fees: For information on publication fees and which article types are subject to charges, visit our website. If your manuscript is accepted for publication and any fees apply, you will be contacted separately about payment during the production process; please follow the instructions in that e-mail. Arrangements for payment must be made before your article is published.

ASM Membership: Corresponding authors may join or renew ASM membership to obtain discounts on publication fees. Need to upgrade your membership level? Please contact Customer Service at Service@asmusa.org.

The ASM Journals program strives for constant improvement in our submission and publication process. Please tell us how we can improve your experience by taking this quick Author Survey.

Thank you for submitting your paper to Spectrum.

Sincerely,
Jeffrey Gardner
Editor
Microbiology Spectrum

Response to comments

Dear Editor:

The sequencing data were already deposited in NCBI under the accession number PRJNA1252862, as noted in lines 571-572.

Re: Spectrum00318-25R2 (Impact of the alkaline volatile trimethylamine on the physiology of Streptomyces venezuelae: an integrated transcriptomic and metabolomics study)

Dear Dr. Xiuhua Pang:

Your manuscript has been accepted, and I am forwarding it to the ASM production staff for publication. Your paper will first be checked to make sure all elements meet the technical requirements. ASM staff will contact you if anything needs to be revised before copyediting and production can begin. Otherwise, you will be notified when your proofs are ready to be viewed.

Data Availability: ASM policy requires that data be available to the public upon online posting of the article, so please verify all links to sequence records, if present, and make sure that each number retrieves the full record of the data. If a new accession number is not linked or a link is broken, provide production staff with the correct URL for the record. If the accession numbers for new data are not publicly accessible before the expected online posting of the article, publication may be delayed; please contact ASM production staff immediately with the expected release date.

Publication Fees: For information on publication fees and which article types have charges, please visit our website. We have partnered with Copyright Clearance Center (CCC) to collect author charges. If fees apply to your paper, you will receive a message from no-reply@copyright.com with further instructions. For questions related to paying charges through RightsLink, please contact CCC at ASM_Support@copyright.com or toll free at +1-877-622-5543. CCC makes every attempt to respond to all emails within 24 hours.

ASM Membership: Corresponding authors may join or renew ASM membership to obtain discounts on publication fees. Need to upgrade your membership level? Please contact Customer Service at Service@asmusa.org.

PubMed Central: ASM deposits all Spectrum articles in PubMed Central and international PubMed Central-like repositories immediately after publication. Thus, your article is automatically in compliance with the NIH access mandate. If your work was supported by a funding agency that has public access requirements like those of the NIH (e.g., the Wellcome Trust), you may post your article in a similar public access site, but we ask that you specify that the release date be no earlier than the date of publication on the Spectrum website.

Embargo Policy: A press release may be issued as soon as the manuscript is posted on the Spectrum Latest Articles webpage. The corresponding author will receive an email with the subject line "ASM Journals Author Services Notification" when the article is available online.

The ASM Journals program strives for constant improvement in our submission and publication process. Please tell us how we can improve your experience by taking this quick Author Survey.

Thank you for submitting your paper to Spectrum.

Sincerely,
Jeffrey Gardner
Editor
Microbiology Spectrum
